# First-in-Human Study of IL15–Activated Cytokine-Induced Killer Cells After Allogeneic HCT Shows Durable Remission and Serotherapy-Associated Immune Reconstitution in Leukemia

**DOI:** 10.1200/JCO-25-01966

**Published:** 2026-04-06

**Authors:** Eva Rettinger, Dirk Heckl, Martin Hutter, Emilia Salzmann-Manrique, Marie Luedtke, Sabine Huenecke, Melanie Bremm, Claudia Cappel, Gesine Bug, Johann Greil, Roland Meisel, Eva Maria Wagner-Drouet, Hubert Serve, Tayfun Güngör, Jan-Henning Klusmann, Thomas Klingebiel, Peter Bader, Halvard Bonig

**Affiliations:** ^1^ Department of Pediatrics, Goethe University Frankfurt, Frankfurt, Germany; ^2^ Institute for Experimental Pediatric Hematology and Oncology (EPHO), Frankfurt, Germany; ^3^ Medical Clinical II, Goethe University Frankfurt, Frankfurt, Germany; ^4^ University Children's Hospital, Heidelberg, Germany; ^5^ Division of Pediatric Stem Cell Therapy, Department of Pediatric Oncology, Hematology and Clinical Immunology, Medical Faculty, Heinrich-Heine-University, Duesseldorf, Germany; ^6^ Johannes Gutenberg-University Mainz, III. Department of Medicine, Germany; ^7^ Department of Pediatric Stem Cell Transplantation and Hematology, Children's Research Center, University Children's Hospital Zurich, Zurich, Switzerland; ^8^ Institute of Transfusion Medicine and Immunohematology, and German Red Cross Blood Center Frankfurt, Goethe University Frankfurt, Frankfurt, Germany; ^9^ University of Washington, Department of Medicine, Division of Hematology, Seattle, WA

## Abstract

**PURPOSE:**

Patients with high-risk (HR) leukemia remain at substantial risk of early relapse, treatment-related toxicity, and poor survival, underscoring the need for effective relapse prevention therapies. To our knowledge, this first-in-human, disease burden–guided study evaluated the feasibility, safety, and efficacy of donor-derived allogeneic interleukin-15–activated cytokine-induced killer cells (IL15-CIK) combining T-cell and natural killer cell properties for post-transplant disease control.

**METHODS:**

In a prospective, multicenter phase I/II trial (EudraCT 2013-005446-11) and an identically designed pilot study, 53 adult and pediatric patients with HR leukemia received 56 courses of IL15-CIK monotherapy after human leukocyte antigen (HLA)–matched or HLA-mismatched transplantation. Treatment intent was categorized as consolidation (13%), preemptive (61%), or salvage (27%) with 169 infusions administered as a single dose (29%) or according to adaptable dose-escalation regimens (71%).

**RESULTS:**

Acute graft-versus-host disease (GVHD) grades 1-2 and grade 3 occurred in 27% and 4% of cases, respectively; no extensive chronic GVHD or treatment-related mortality was observed. IL15-CIK–associated adverse events were predominantly mild. Disease clearance, assessed by the cumulative incidence of complete molecular remission, peaked at day 700, reaching 74% in the preemptive and 13% in the salvage setting. The five-year progression-free survival was 50% overall and highest (69%) in pediatric acute myeloid leukemia. The five-year overall survival (OS) was 71% in the consolidation, 61% in the preemptive, and 20% in the salvage setting. Multivariable analysis demonstrated significantly lower relapse rates with Campath compared with ATG, superior OS in myeloid malignancies, and reduced IL15-CIK efficacy in advanced disease. The median follow-up was 7.3 years.

**CONCLUSION:**

IL15-CIK monotherapy is feasible and safe and demonstrates promising relapse-preventive activity after hematopoietic stem-cell transplantation. Clinical outcomes are strongly influenced by disease burden at treatment initiation and previous serotherapy, supporting optimized patient selection and timing in future post-transplant immunotherapeutic strategies.

## Graphical abstract

**Figure:**
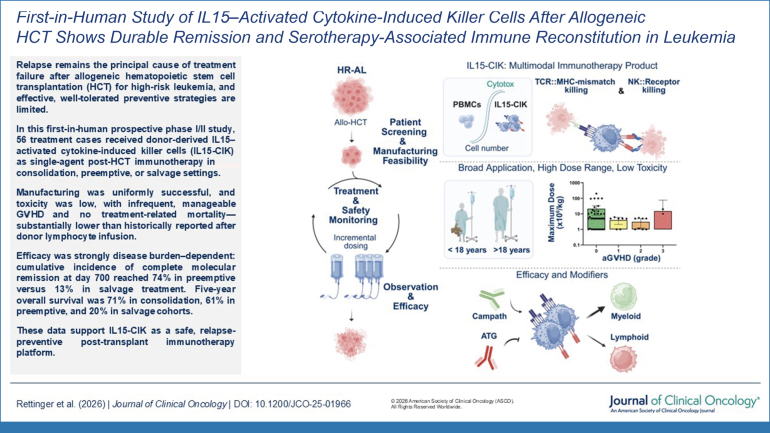

## INTRODUCTION

Allogeneic hematopoietic stem-cell transplantation (HCT) remains the cornerstone of curative-intent therapy for patients with high-risk (HR) leukemia.^1,2^ However, early post-HCT relapse is associated with poor outcomes, particularly in children and patients with high toxicity burden, for whom effective salvage options are often unapproved or unavailable.^3-5^ These limitations highlight the pressing need for preventive, well-tolerated, and accessible post-HCT immunotherapies.

**CONTEXT**

**Key Objective**
This is, to our knowledge, a first-in-human study evaluating the feasibility of IL15-CIK single-agent therapy, transplant-related factors influencing safety and response, and its efficacy as post–hematopoietic stem-cell transplantation immunotherapy in high-risk leukemia across consolidation, preemptive, and salvage settings.
**Knowledge Generated**
Therapy was feasible and well-tolerated and had no treatment-related mortality, mild adverse events (AEs), and a low incidence of severe graft-versus-host disease. At a median follow-up of 7.3 years, IL15-CIK achieved the 5-year overall survival of 71% in consolidation, 61% in preemptive, and 20% in relapse settings, reflecting increasing disease burden and favoring myeloid malignancies.
**Relevance *(C. Craddock)***
IL15-CIK cells demonstrate promising clinical activity in patients transplanted for high risk leukemia and in patients with disease relapse post-transplant. These data support future evaluation of IL15-Activated CIK both as a strategy to improve outcomes in patients allografted for high risk acute leukemia and as a novel salvage strategy in relapsed disease.*
*Relevance section written by *JCO* Associate Editor Charles Craddock, MD.

Donor lymphocyte infusion (DLI) represents the benchmark post-HCT intervention—supported by guidelines to which we contributed.^6,7^ In preemptive settings, DLI induces complete molecular remission (CMR) in approximately 50% of patients,^6^ and when used as a post-HCT consolidation, the 2- to 4-year survival approaches 70%.^8,9^ However, efficacy is limited in overt relapse, with the 5-year survival being around 20%.^10^ Moreover, substantial toxicity persists, including acute and chronic graft-versus-host disease (aGVHD: 28%-42%; cGVHD up to 59%) and up to 14% 1-year nonrelapse mortality (NRM).^11,12^

By contrast, cytokine-induced killer cells (CIK), an in vitro–expanded donor lymphocyte population with combined T-cell– and natural killer (NK)–like cytotoxicity,^13-15^ enable targeted antileukemic activity while limiting alloreactivity through NK-mediated self-recognition. However, clinical studies of interleukin (IL)-2–activated CIK therapy (IL2-CIK) have been limited to small adult cohorts with advanced disease and concomitant conventional DLI (cDLI).^16-22^

To overcome these limitations, we developed IL15-stimulated CIK therapy (IL15-CIK), which demonstrated superior expansion, persistence, and cytotoxicity in preclinical models.^23,24^ Building on feasibility data,^23-26^ a hospital exemption (PEI.A.11630.01.1) enabled clinical administration of IL15-CIK in a pilot study (PS) and a prospective multicenter phase I/II clinical trial (CT; EudraCT 2013-005446-11), evaluating safety and antileukemic activity in HR hematologic malignancies.^27^

Here, we report outcomes from 56 pediatric and adult cases with HR leukemia treated with IL15-CIK after HCT. These results support IL15-CIK as a safe and effective post-transplant alternative to DLI for relapse prevention.

## METHODS

### Patient Cohort (prescreening)

Details of the cohorts are provided in the Results section and the Data Supplement (online only). Written informed consent was obtained from all participants before the first IL15-CIK infusion, and the study was approved by the institutional ethics committee (approval No. 407/14).

### Eligibility Criteria (screening phase)

**FIG 1.**
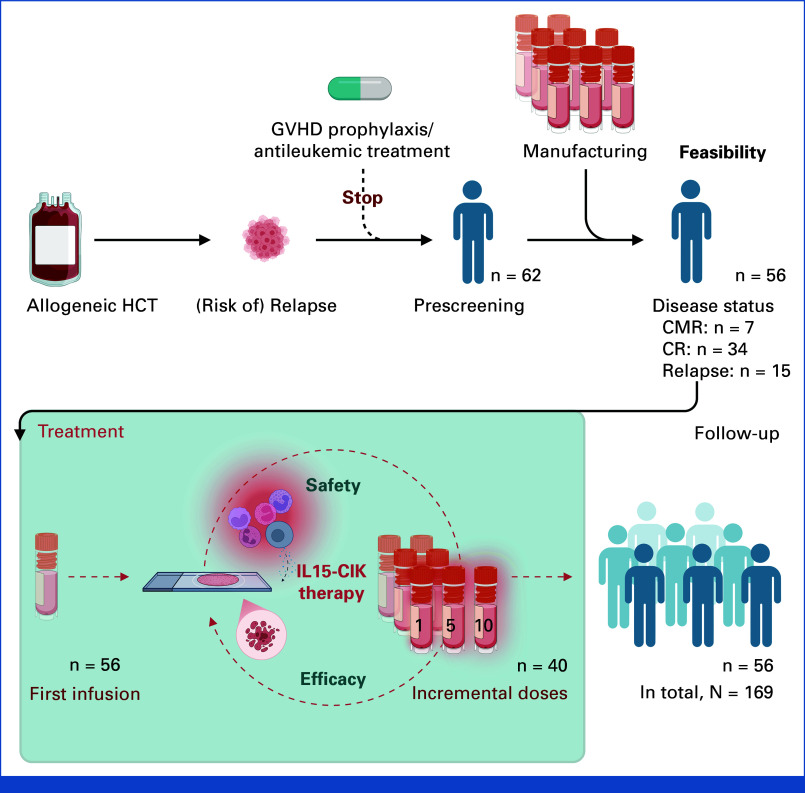
Treatment algorithm and patient flow for allogeneic IL15-CIK. Patients who had undergone HCT for high-risk leukemia and remained at risk of relapse were eligible for donor-derived IL15-CIK after verification of manufacturing. Patients were required to be off antileukemic therapy and immunosuppression and free of acute GVHD >grade 1 at the first infusion (baseline). IL15-CIK was given in CMR (n = 7), in CR (non-CMR; n = 34), or during relapse (>120 days post-transplant without successful reinduction; n = 15). Consolidation (CMR) and preemptive (CR/non-CMR) treatment was administered for relapse prevention. Salvage IL15-CIK was given for relapse management (≥5% BM blasts). Cases were monitored weekly for safety. Disease status was assessed monthly to guide additional dosing. Considering the individual relapse risk, IL15-CIK could be administered as a single infusion or in incremental doses (dose steps: 1.0, 5.0, 10.0, 100.0 × 10^6^ T cells/kg per infusion), with a minimum interval of 4 to 6 weeks between infusions, in the absence of dose-limiting toxicity or worsening GVHD. A total of 169 infusions were administered to 56 cases across five German centers, with a follow-up of at least 6 months after the final infusion. BM, bone marrow; CMR, complete molecular remission; CR, complete remission; GVHD, graft-versus-host disease; HCT, hematopoietic stem-cell transplantation.

Eligible cases were children and adults (>1 to <80 years) with impending relapse after HCT or overt relapse occurring >120 days post-transplant after completing induction therapy (Fig 1). Inclusion in the preemptive cohort required complete remission (CR/non-CMR) with evidence of impending relapse (recurrent disease-specific minimal/measurable residual disease [MRD], cytogenetic marker positivity, or declining donor chimerism). General eligibility criteria included the following: Karnofsky/Lansky score ≥50%, the absence of aGVHD >grade 1 or any cGVHD, and no concomitant immunosuppressive, immunomodulatory, corticosteroid, or antileukemic therapy, except tyrosine kinase inhibitors.

### IL15-CIK Treatment Protocol (treatment phase)

IL15-CIK was administered as a single dose or repeated doses at 4- to 6-week safety intervals, with halt/stop rules (Data Supplement), without preconditioning, lymphodepletion, graft-versus-host disease (GVHD) prophylaxis, or antileukemic therapy. Stepwise dose escalation of potentially alloreactive CD3^+^CD56^−^ T cells was performed with doses of 1.0, 5.0, 10.0, and 100.0 × 10^6^/kg body weight of the recipient per infusion, with a minimum interval of 4–6 weeks between infusions, which could be adapted on a case-by-case basis in consultation with the central study team.

Treatment was discontinued upon achievement of CMR, completion of 1 year of therapy, or after eight infusions, whichever occurred first. Cases with disease recurrence after an initial response and treatment discontinuation were eligible for additional IL15-CIK.

### IL15-CIK, an Approved Multifunctional DLI

Since 08/2011, IL15-CIK has been administered under individual compassionate-use approvals from the local regulatory authority (Regierungspräsidium Darmstadt, Germany).^27^ In 06/2014, IL15-CIK received authorization under the hospital exemption regulation for advanced medicinal products (ATMPs; PEI A.11630.01.1). The source material and IL15-CIK manufacturing have been described previously.^27^

### Primary Study End Points

Feasibility was assessed in the preemptive cohort by evaluating timelines from request to product release, including donor clearance, lymphocyte collection, ex vivo manufacturing success and duration, quality control, regulatory compliance, and administration rates.

Safety was evaluated across all cohorts before, during, and weekly after each IL15-CIK through clinical assessments, laboratory tests, and monitoring for adverse events (AEs) and dose-limiting toxicities (DLTs), including GVHD, cytokine release syndrome (CRS), and neurologic, renal, hepatic, and hematologic toxicities.

### Secondary Study End Points

Clinical outcomes were assessed at baseline, 4-6 weeks after each infusion, on day 100 after the first infusion, and throughout at least 6 months of follow-up. End points included overall survival (OS), progression-free survival (PFS), cumulative incidence of relapse (CIR), relapse-related mortality (RRM), and NRM. Objective responses were the highest cumulative incidence of CMR (CI-CMR) at day 400 or 700 in the preemptive and salvage cohorts.

### Statistical Analysis

Statistical analyses were performed using R. Demographic, clinical, and T-cell dose data were summarized descriptively. Group comparisons were performed using Fisher's exact, Pearson's chi-square, Wilcoxon rank-sum, or Kruskal-Wallis rank-sum tests. Median follow-up was estimated using the reverse Kaplan-Meier method. OS and PFS were estimated using the Kaplan-Meier method and compared using log-rank tests. Cumulative incidences were used to estimate CIR, CI-CMR, and NRM. Testing was performed using Gray's test. Salvage cases were excluded from PFS, CIR, and NRM analyses, and consolidation cases from CI-CMR analyses. Associations between aGvHD and outcomes were assessed using time-dependent Cox regression, whereas factors associated with OS and CIR were evaluated using multivariable Cox regression and semiparametric Fine-Gray competing risk modeling, respectively. Longitudinal immune parameters were analyzed using mixed-effects models with cubic B-splines and compared using paired Wilcoxon and Friedman tests. All tests were two-sided with α = .05; 95% CIs were reported.

The additional methodology is provided in the Data Supplement.

## RESULTS

### Disease Status Stratification of IL15-CIK Post-HCT

Between 2011 and 2020, IL15-CIK was administered in 53 patients. Three received a second course after repeat HCT from an alternative donor, resulting in 56 treatment cases (Fig 1 and Data Supplement, Fig S1). Cases were treated within a PS until March 2016 and were subsequently enrolled in a multicenter, prospective phase I/II trial. All treatments were delivered without concomitant antileukemic therapy under a harmonized protocol. Based on disease status at treatment initiation (baseline), cases were stratified into a consolidation cohort (seven cases in CMR with HR features), a preemptive cohort (34 cases in CR with impending relapse and <5% bone marrow [BM] blasts), and a salvage cohort (15 cases with overt relapse and ≥5% BM blasts). Across cohorts, we assessed safety, relapse prevention intent (consolidation plus preemptive cohorts; n = 41), and relapse management (salvage cohort).

### Human Leukocyte Antigen–Matched Versus HLA-Mismatched Donor Settings

The cohort constitutes a representative post-HCT HR leukemia population, with 21 cases (37.5%) receiving >1 transplant. Key transplant and IL15-CIK variables are summarized in Table 1. In the relapse prevention group, significant differences between ≥9/10 human leukocyte antigen (HLA)–matched donor (MD) and ≤8/10 HLA-mismatched donor (MMD) settings were observed for age (*P* = .023), remission status at HCT, number of previous transplants, stem-cell source, graft manipulation and composition, serotherapy type, IL15-CIK treatment intent, and time from HCT to first IL15-CIK (all *P* < .001).

**TABLE 1.** Case Characteristics: MD Versus MMD

Characteristic	Total (n = 56)	MD Relapse Prevention (n = 22)	MMD Relapse Prevention (n = 19)	*P*	Salvage (n = 15)
Sex, No. (%)				.754	
Female	22 (39)	7 (32)	7 (37)		8 (53)
Male	34 (61)	15 (68)	12 (63)		7 (47)
Age at first IL15-CIK, years				.657	
Median (range)	10.1 (0.6-71.9)	10.5 (1.3-71.9)	9.9 (2.0-17.6)		8.8 (0.6-58.7)
Age group at first IL15-CIK, No. (%)				**.023**	
<18 years	44 (79)	16 (73)	19 (100)		9 (60)
Median (range)	8.6 (0.6-17.6)	8.3 (1.3-15.7)	9.9 (2.0-17.6)		3.7 (0.6-16.0)
≥18 years	12 (21)	6 (27)	0 (0)		6 (40)
Median (range)	58.6 (20.0 -71.9)	69.5 (20.0-71.9)	—		54.3 (23.5-58.7)
Disease myeloid, No. (%)	32 (57)	12 (55)	8 (42)	.536	12 (80)
AML	29 (52)	10 (45)	7 (37)		12 (80)
CML	1 (2)	0 (0)	1 (5)		0 (0)
CMML	1 (2)	1 (5)	0 (0)		0 (0)
MRC	1 (2)	1 (5)	0 (0)		0 (0)
Lymphoblastic, No. (%)	24 (43)	10 (45)	11 (58)		3 (20)
Pre–/pro–B-ALL	15 (27)	7 (32)	7 (37)		1 (7)
Pre–T-ALL	9 (16)	3 (14)	4 (21)		2 (13)
Remission status at HCT, No. (%)				**<.001**	
1. CR	17 (30)	14 (64)	1 (5)		2 (13)
2. CR	18 (32)	5 (23)	7 (37)		6 (40)
≥3. CR	4 (7)	2 (9)	2 (11)		0 (0)
NR	17 (30)	1 (5)	9 (47)		7 (47)
No. of HCTs, No. (%)				**<.001**	
First	35 (63)	21 (95)	5 (26)		9 (60)
Second	18 (32)	1 (5)	13 (68)		4 (27)
≥Third	3 (5)	0 (0)	1 (5)		2 (13)
Donor, No. (%)				**—**	
MSD	2 (4)	2 (9)			0 (0)
MUD	26 (46)	20 (91)			6 (40)
MMFD	27 (48)		18 (95)		9 (60)
MMUD	1 (2)		1 (5)		0 (0)
Stem-cell source, No. (%)				**<.001**	
BM	16 (29)	13 (59)	1 (5)		2 (13)
PBSC	40 (71)	9 (41)	18 (95)		13 (87)
In vitro T-cell depletion, No. (%)				**<.001**	
No	28 (50)	20 (91)	1 (5)		7 (47)
Yes	28 (50)	2 (9)	18 (95)		8 (53)
Graft composition					
CD34^+^ × 10^6^/kg				**.001**	
Median (range)	8.3 (1.7-40.0)	6.4 (1.7-16.5)	12.4 (2.6-40.0)		11.3 (2.5-21.7)
CD3^+^ × 10^6^/kg				**<.001**	
Median (range)	1.0 (0.0-500.4)	45.8 (0.1-500.4)	0.1 (0.0-13.3)		1.3 (0.2-331.5)
Serotherapy, No. (%)				**<.001**	
No	4 (7)	2 (9)	0 (0)		2 (13)
ATG	30 (54)	17 (77)	4 (21)		9 (60)
Campath	21 (38)	3 (14)	14 (74)		4 (27)
OKT3	1 (2)	0 (0)	1 (5)		0 (0)
ATG dose, mg/kg				**.010**	
Median (range)	60.0 (22.2-120.0)	60.0 (40.0-83.6)	40.0 (27.7-40.0)		30.6 (22.2-120.0)
Campath dose, mg/kg				**.014**	
Median (range)	0.5 (0.3-17.6)	1.4 (1.2-2.0)	0.5 (0.3-17.6)		0.4 (0.3-0.8)
IL15-CIK therapy, treatment intent, No. (%)					
Consolidation	7 (13)	0 (0)	7 (37)		0 (0)
Preemptive	34 (61)	22 (100)	12 (63)		0 (0)
Salvage	15 (27)	0 (0)	0 (0)		15 (100)
IL15-CIK: study group, No. (%)					
PS	33 (59)	9 (41)	15 (79)		9 (60)
CT	23 (41)	13 (59)	4 (21)		6 (40)
Time from HCT to first IL15-CIK, months				**<.001**	
Median (range)	4.1 (0.7-43.9)	6.3 (2.4-17.8)	1.7 (0.7-31.9)		4.0 (1.0-43.9)
Time from HCT to first IL15-CIK, No. (%)				**<.001**	
<100 days	24 (43)	2 (9)	16 (84)		6 (40)
≥100 days	32 (57)	20 (91)	3 (16)		9 (60)
Previous treatment course, No. (%)				.091	
No	53 (95)	22 (100)	16 (84)		15 (100)
Yes	3 (5)	0 (0)	3 (16)		0 (0)

NOTE. Testing was performed for differences between ≥9/10-HLA–matched donors and ≤8/10-HLA–mismatched donors. The bold entries represent statistically significant *p*-values.

Abbreviations: ATG, antithymoglobulin; BM, bone marrow; cALL, common acute lymphoblastic leukemia; CML, chronic myeloid leukemia; CMML, chronic myelomonocytic leukemia; CR, complete remission; CT, clinical trial; HCT, hematopoietic stem-cell transplantation; MD, HLA-matched donor; MMD, HLA-mismatched donor; MMFD, HLA-mismatched family donor; MMUD, HLA-mismatched, unrelated donor; MRC, myelodysplasia-related changes; MSD, HLA-matched sibling donor; MUD, HLA-matched unrelated donor; NR, nonremission; PBSC, peripheral blood stem cells; Post-HCT IL15-CIK therapy, IL15-activated cytokine-induced killer cells; PS, pilot study; T-ALL, T-cell ALL.

Stem-cell grafts consisted of BM (n = 16, 29%) or peripheral blood stem cells (PBSC; n = 40, 71%) and were equally derived from MD and MMD donors (each n = 28). Ex vivo T-cell depletion was performed in all MMD PBSC grafts. Serotherapy included antithymocyte globulin (ATG; n = 30, 54%), Campath (n = 21, 38%), or OKT3 (n = 1, 2%). The median interval from HCT to first IL15-CIK was 4.1 months (range, 0.7-43.9).

### Feasibility of On-Demand Manufacturing With Adaptive Dosing

Timely availability of cellular immunotherapy is critical for patients at imminent relapse risk. Feasibility was confirmed in 23 preemptive cases, with a 100% manufacturing success rate. IL15-CIK batches were centrally manufactured in Frankfurt, met predefined release criteria,^27^ and were distributed to five centers across Germany. The median time from request to release was 21 days (range, 10-42).

A single dose was administrated in 16 cases; 40 required incremental dose adjustment for disease control. Of the 56 cases, 32 (57%) followed the stepwise dose-escalation scheme, 10 (18%) were treated with a reduced dose because of HLA disparity or GVHD, and 14 (25%) were treated with an increased dose based on relapse risk. Overall, cases received a median of two infusions (range, 1-10), administered at intervals of 4–6 weeks, with a median cumulative T-cell dose of 6.5 × 10^6^/kg (range, 1.0-721.4 × 10^6^/kg; Data Supplement, Table S1). Cumulative dosing did not differ between the donor type (*P* = .059) although the third infusion dose was significantly higher in MD recipients (*P* = .039; Data Supplement, Table S2). Overall, 118 IL15-CIK doses were administered for relapse prevention (MD n = 68; MMD n = 50), with no evidence of dose-dependent toxicity (Data Supplement, Fig S2) or efficacy.

### Safety and Predominantly Non–IL15-CIK-Attributed Toxicities

Given the known risks of allogeneic cell therapies, safety was closely monitored (Data Supplement, Tables S3A and S3B, S4). AEs after IL15-CIK are summarized in Table S3A, with no safety differences observed between the PS and the CT (PS *v* CT case characteristics: Data Supplement, Table S5).

Most AEs were mild (n = 57 of 78; 73%). Moderate events occurred in 15 cases (19%) and included viral infections/reactivations (n = 10), bacterial septicemia (n = 2), periproctic abscess (n = 1), osteomyelitis (n = 1), and grade 3 atopic eczema (n = 1). Severe adverse events (SAEs) were rare (n = 3; 4%) and limited to viral infections/reactivations. Three fatal events occurred during follow-up: undifferentiated progressive lung infiltration (relapse: day 26; death: 3.7 months), CNS aspergillosis (death: 3.6 months), and respiratory syncytial virus infection (death: 4.9 months). None were considered related to the study drug or infusion.

AEs possibly related to IL15-CIK were infrequent and predominantly mild (n = 17; Data Supplement, Table S3A), including flu-like symptoms (n = 6), fever (n = 4), grade 1 CRS (n = 1), allergic reactions (n = 1), and skin manifestations (dermatitis n = 2; exanthema and erythema n = 1 each). One moderate event, grade 3 atopic eczema, was considered IL15-CIK–related. All AEs resolved with standard treatment, except for the three fatal cases.

GVHD after IL15-CIK was rare and not associated with IL15-CIK dosing (Data Supplement, Fig S2; Data Supplement, Table S3B): grade 1 aGVHD occurred in seven cases (13%), grade 2 in eight (14%), and grade 3 in two cases (4%). Grade 3 aGVHD developed after escalating doses in one HLA-matched preemptive case (T-cell doses: 1.0, 5.0, 8.3, 10.0, and 10.0 [×10^6^/kg] per infusion) and one HLA-mismatched consolidation case (T-cell doses: 1.0, 10.0, and 20.0 [×10^6^/kg] per infusion), with infusions administered at 4–6-week intervals. GVHD occurred on days 158 and 71 after the first and on days 18 and 19 after the last infusion. Affected organs included skin and liver in the preemptive case and skin, liver, and GI tract in the consolidation case, which progressed to limited cGVHD. Both cases were manageable and reversible with standard treatment (calcineurin inhibitors, corticosteroids, and extracorporeal photopheresis in one case). No grade 4 aGVHD was observed. Limited cGVHD developed in four cases (n = 2 each in the MD and MMD settings).

DLTs were defined as grade 3-4 aGVHD, extensive cGVHD, or unmanageable or permanently harmful toxicity. At last follow-up, all patients with grade 3 aGVHD or cGVHD were alive in CMR and had discontinued immunosuppressive therapy and none were considered to have experienced DLTs.

NRM occurred after CMR in six heavily pretreated patients (Data Supplement, Table S4), because of infections (n = 3), thrombotic microangiopathy (n = 1), or transplant-associated organ toxicity (renal and cardiac failure; n = 2). A significant negative association between days from HCT to first IL15-CIK and NRM (hazard ratio of 0.05 [95% CI, 0 to 0.82]; *P* = .036; univariable Fine-Gray model) indicates that NRM was primarily related to transplant-associated factors rather than IL15-CIK.

Overall, IL15-CIK demonstrated a favorable safety profile, with therapy-related GVHD and AEs being infrequent, mild, and manageable.

### Outcomes at Day 100 and CI-CMR at Day 700 After First Infusion

**FIG 2.**
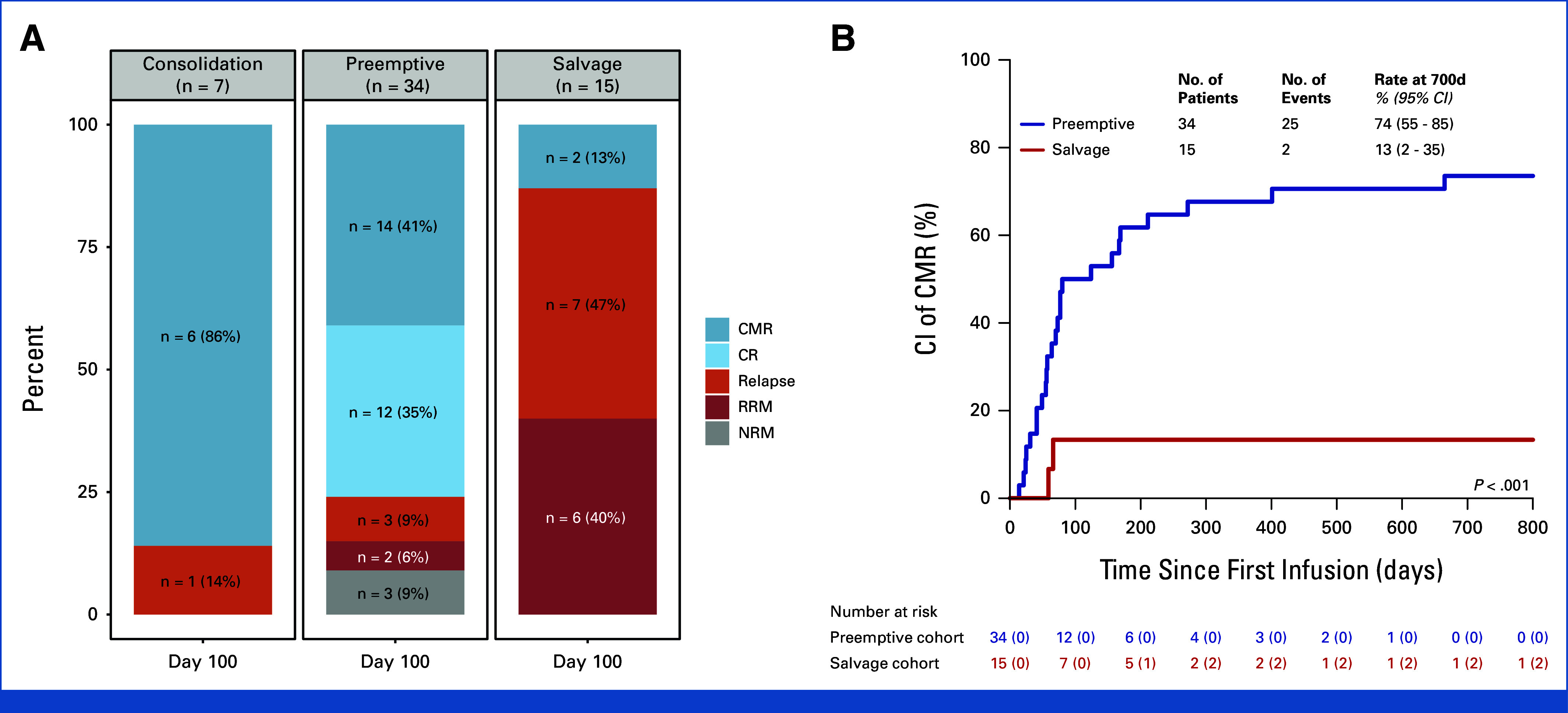
Clinical outcomes by IL15-CIK treatment intent. Based on treatment intent and disease status at baseline—CMR, CR/non-CMR, or overt relapse—cases were allocated to the consolidation, preemptive, or salvage cohorts. (A) Clinical outcomes, including disease status (CMR, CR/non-CMR, relapse) and death because of relapse (RRM) or toxicity (NRM), were assessed at day 100 after first infusion in the entire study population (N = 56). (B) To objectify treatment efficacy, the cumulative incidence of CMR (CI-CMR) was evaluated at day 700 after the first infusion in the overall cohort. CMR, complete molecular remission; CR, complete remission; NRM, nonrelapse mortality; RRM, relapse-related mortality.

At baseline, seven cases were in CMR (consolidation), and 34 in CR/non-CMR (preemptive), and 15 presented with relapse (salvage; Data Supplement, Fig S1A). By day 100, CMR was maintained in 6 of 7 consolidation cases (86%), with one relapse (14%). In the preemptive cases, 14 of 34 (41%) achieved CMR, 12 of 34 (35%) maintained CR (stable disease), and three relapses occurred (9%), resulting in RRM in two cases (6%). NRM was observed in three cases (9%). In the salvage cases, CMR was achieved in 2 of 15 (13%), whereas 7 of 15 (47%) remained in stable disease and 6 of 15 experienced RRM (40%; Fig 2A).

In univariable analysis, remission status at HCT (*P* = .041), serotherapy type (*P* = .004), and the interval from HCT to immune intervention (*P* = .023), but not graft composition, were associated with disease status at day 100 (Data Supplement, Fig S3).

The significantly lower response rate in the salvage cohort compared with the preemptive cohort (CI-CMR, day 700; *P* < .001) underscores the importance of relapse prevention after HCT in HR leukemia (Fig 2B). This effect was particularly pronounced in pediatric acute myeloid leukemia (pAML), which showed the greatest benefit from preventive therapy (Data Supplement, Fig S4; Data Supplement, Tables S6-S8).

### Serotherapy and Immune Modulation

Serotherapy targeting the lymphocyte compartment is an integral part of HCT to prevent GVHD but may affect subsequent lymphocyte-based immunotherapy. To note, IL15-CIK—generated from unstimulated leukaphereses—was neither intended to alter the T-cell–depleted PBSC graft composition nor to serve as an immune reconstitution modality.

**FIG 3.**
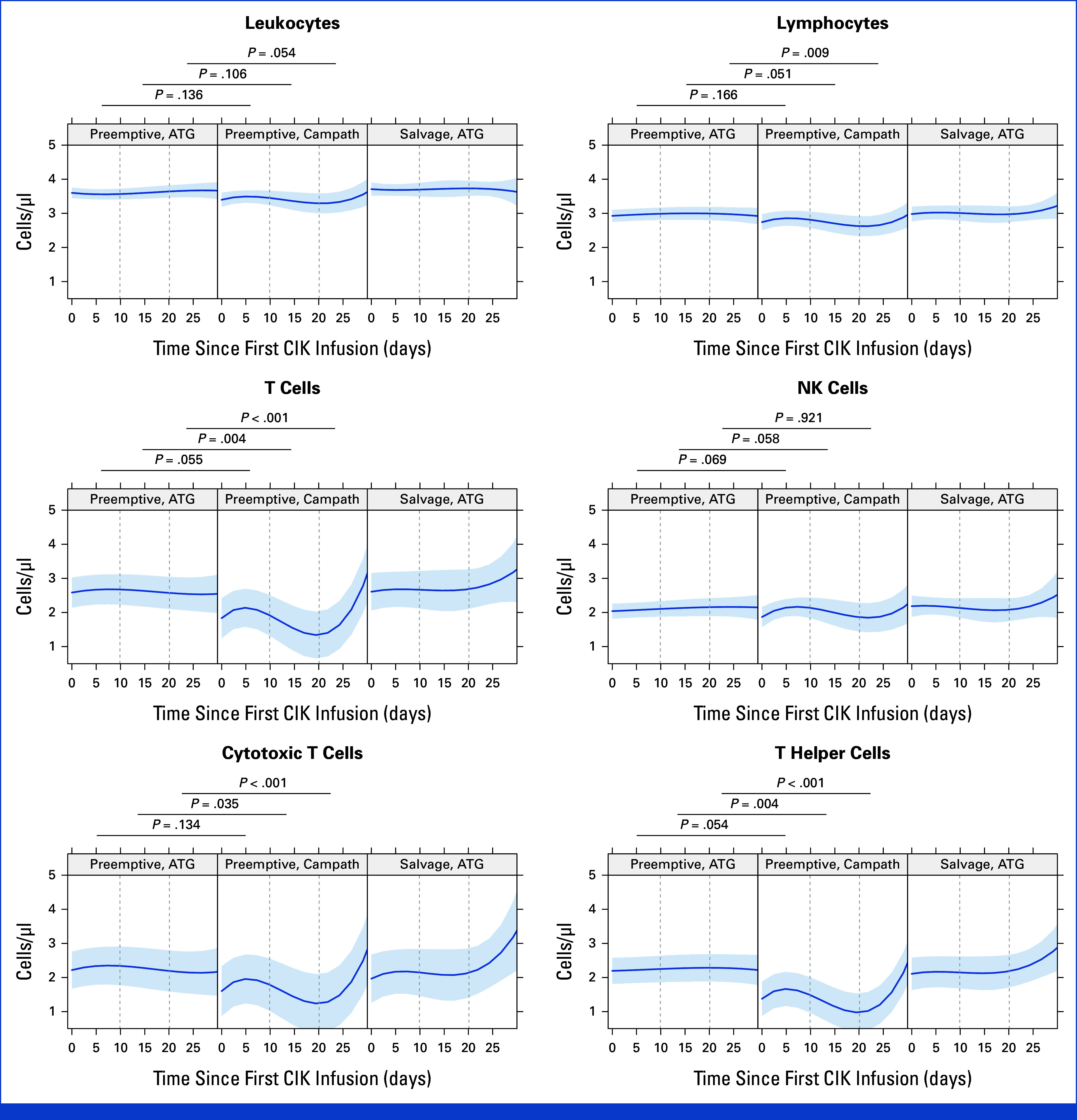
Immune cell dynamics after IL15-CIK. Leukocyte trajectories during the first month postinfusion are illustrated relative to preinfusion levels, stratified by previous serotherapy and treatment intent (preemptive [CR/non-CMR], salvage [relapse]), reflecting differences in disease burden. In the preemptive cohort, Campath pretreatment was associated with delayed yet substantial leukocyte expansion, diverging from the more attenuated and stable trajectory observed after ATG exposure in the preemptive and salvage cohorts. Corresponding changes in the lymphocyte compartment—highlighting enhanced reconstitution of cytotoxic T cells, T-helper cells, and NK cells—are shown in the adjacent panels. These patterns indicate that IL15-CIK can promote recovery of both innate and adaptive immunity, particularly when administered in the preemptive setting after Campath pretreatment. By contrast, the limited leukocyte expansion observed in salvage cases—long after transplantation and in the absence of residual serotherapy—likely reflects both the combined effects of high disease burden and impaired immune reserve. Besides appropriate serotherapy selection, this underscores the importance of administering immune cell interventions ideally during CR and lymphopenia phases. CMR, complete molecular remission; CR, complete remission; NK, natural killer.

In this study, serotherapy significantly influenced leukocyte kinetics—assessed weekly after IL15-CIK (Fig 3; Data Supplement, Fig S5). Preemptive cases pretreated with Campath (0.1 mg/kg/once daily for 4 consecutive days) compared with ATG (10-20 mg/kg/once daily for 3–4 consecutive days) showed earlier and more sustained leukocyte expansion after IL15-CIK.

To further characterize immune responses, cytokine secretion profiles and cytotoxic effector signatures were analyzed at baseline, 1 hour, 1 week, and 2-4 weeks after IL15-CIK (Data Supplement, Fig S6). IL15-CIK induced moderate increases in IL-2 and IL-10, a marked increase in interferon-γ (Data Supplement, Fig. S6A), and upregulation of tumor necrosis factor-α, FAS, FASL, perforin, granzyme A, and granulysin, indicating engagement of nonapoptotic and apoptotic signaling pathways (Data Supplement, Fig S6B).

Together, these findings highlight serotherapy as a potentially important determinant of post-HCT immune dynamics and demonstrate potent, multimodal immunologic and antileukemic activity induced by IL15-CIK.

### Leukemic Burden Predicts 5-Year Survival Post–IL15-CIK

The median follow-up of the cohort was 7.3 years (range, 1.2-11.7; n = 53 patients). At the 3-year follow-up, 27 patients were alive and 26 had died: six from NRM, 16 from RRM (including four lost to follow-up after relapse), and four from RRM or NRM after subsequent HCT outside the study (Data Supplement, Fig S1B).

**FIG 4.**
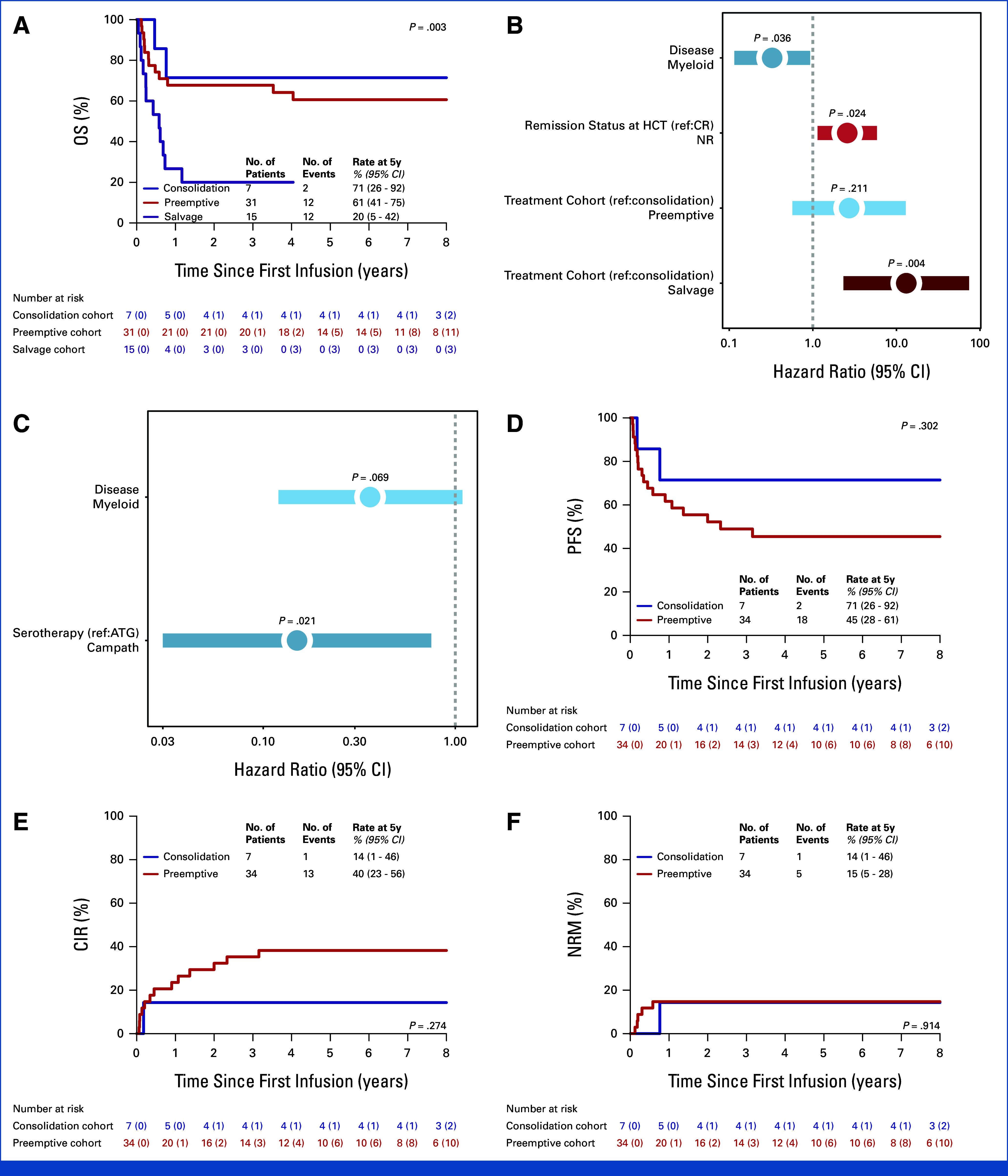
Survival outcomes in the overall cohort. (A) The 5-year OS estimates were 71% in the consolidation cohort and 61% in the preemptive cohort; in the salvage cohort, three cases achieved long-term survival, corresponding to a 5-year OS estimate of 20% (*P* = .003). Forest plots with hazard ratios and 95% CIs for the multivariable Cox regression of OS and for the semiparametric Fine-Gray competing risk model of CIR are shown in (B) and (C). Favorable OS was associated with myeloid disease (*P* = .036). Worse OS was associated with NR status at HCT compared with CR (*P* = .024) and with IL15-CIK treatment in the salvage setting compared with consolidation intent (*P* = .004). In the CIR analysis, serotherapy type emerged as a significant predictor: Campath, compared with ATG, was associated with a lower incidence of relapse (*P* = .021). Regarding relapse prevention intent, (D) the 5-year PFS, (E) the CIR estimates, and (F) NRM estimates were 71%, 14%, and 14% in the consolidation cohort and 45%, 40%, and 15% in the preemptive cohort. Notably, consolidation in CMR cases demonstrated low CIR and NRM rates, suggesting IL15-CIK as a viable post-HCT strategy for hematologic malignancies at high risk of relapse. The risk tables provide the numbers of censored patients in brackets. CIR, cumulative incidence of relapse; CMR, complete molecular remission; CR, complete remission; HCT, hematopoietic stem-cell transplantation; NR, nonremission; NRM, nonrelapse mortality; OS, overall survival; PFS, progression-free survival.

The 5-year OS estimates were 71%, 61%, and 20% in the consolidation, preemptive, and salvage cohorts (*P* = .003; Fig 4A; Data Supplement, Table S9).

Multivariable analysis revealed myeloid malignancy as independently associated with improved OS (*P* = .036), whereas high leukemic burden at first infusion (≥5% BM blasts; *P* = .004) and refractory disease at HCT (*P* = .024) were associated with inferior OS (Fig 4B). Serotherapy remained the only factor significantly associated with CIR (*P* = .021; Fig 4C), which will need further validation and exclusion of confounding factors in a larger cohort.

The 5-year PFS was 71% and 45% in the consolidation and preemptive cohort (Fig 4D), resulting in a combined PFS of 50% (Data Supplement, Fig S7A). At 5 years, both CIR and NRM were 14% in the consolidation cohort, compared with 40% and 15% in the preemptive cohort (Figs 4E and 4F). In the relapse prevention cohort, the CIR and NRM were 36% and 15% (all patients*; Data Supplement, Fig S7B and S7C). Preliminary subgroup analyses indicate increased responsiveness to IL15-CIK in selected subgroups (eg, pAML) and the presence of cohorts at increased risk of NRM, including heavily pretreated and relapse/refractory PS cases (Data Supplement, Figs S7-S9). Accordingly, OS was lower in the PS cohort, whereas PFS, CIR, and NRM did not differ between the PS and CT cohorts (Data Supplement, Fig S9; Data Supplement, Tables S4 and S5).

**FIG 5.**
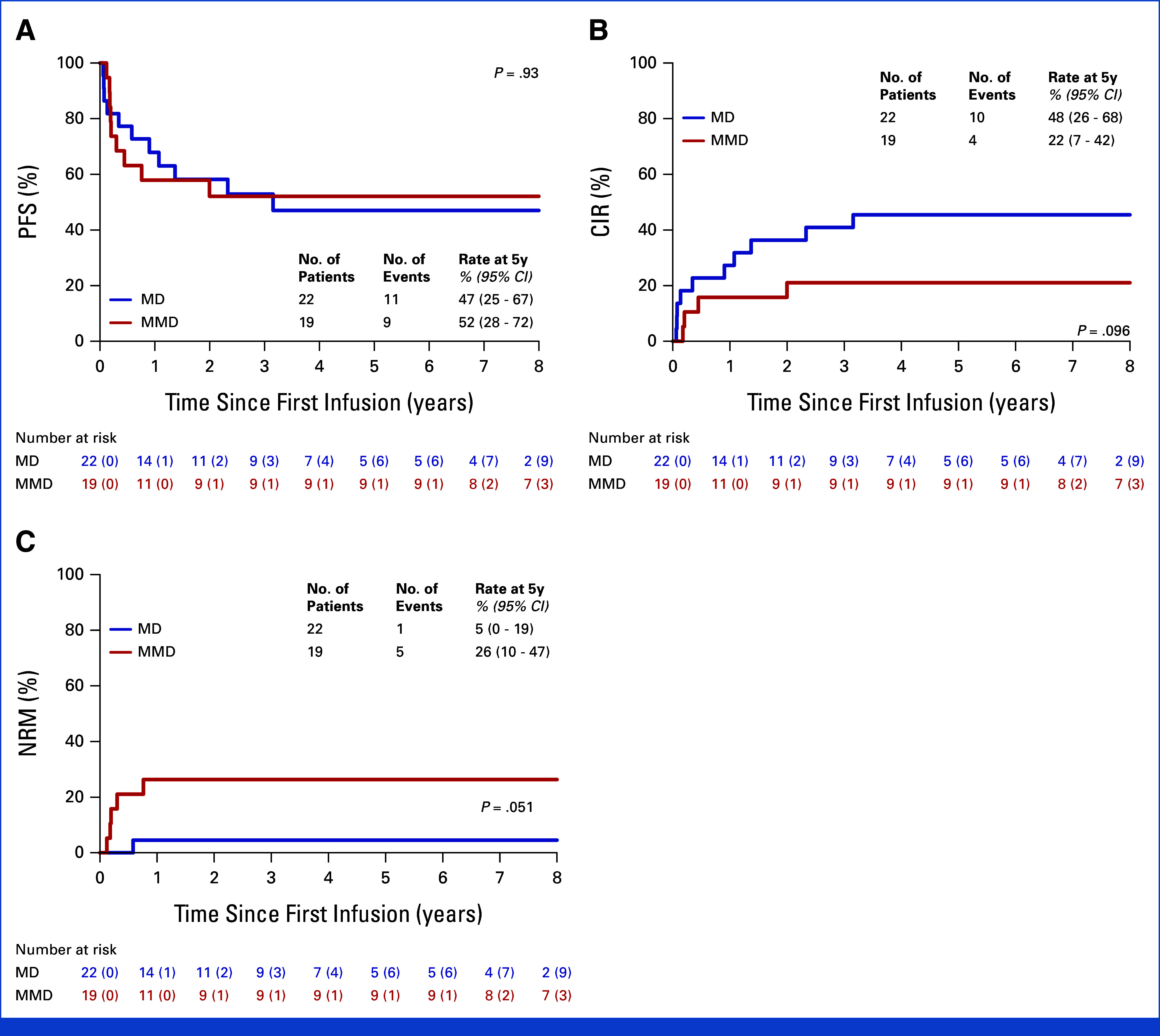
Survival outcomes according to the donor type. (A) PFS, (B) the CIR and (C) NRM at 5 years by donor type are shown. PFS, CIR, and NRM estimates were 47%, 48%, and 5% after HLA-MD and 52%, 22%, and 26% after HLA-MMD post-transplantation IL15-CIK therapy. The risk tables provide the numbers of censored patients in brackets. CIR, cumulative incidence of relapse; HLA, human leukocyte antigen; MD, matched donor; MMD, mismatched donor; NRM, nonrelapse mortality; PFS, progression-free survival.

Despite potential confounding by donor type (Fig 5) or other HCT-related variables, IL15-CIK appears to be broadly applicable across HR leukemia settings, as indicated by early disease assessment at day 100 (Data Supplement, Fig S10), with serotherapy, remission status at HCT, and disease burden potentially informing treatment planning.

## DISCUSSION

HCT is a key therapeutic option for HR, primary refractory, and relapsed leukemia, yet prognosis declines rapidly after post-HCT relapse,^28,29^ underscoring the need for broadly applicable and well-tolerated relapse prevention strategies. IL15-CIK, a multifunctional donor-derived immune cell product, represents one approach that has not previously been evaluated systematically as a single agent in a larger prospective clinical setting.

Here, we report combined results from a prospective CT and a parallel PS, comprising 56 HR leukemia cases treated with IL15-CIK monotherapy across uniform relapse preventive and relapse management indications after HCT. IL15-CIK demonstrated feasible on-demand manufacturing, favorable tolerability, and relapse prevention potential. Beyond early disease assessment at day 100, long-term outcomes—including 5-year OS and PFS influenced by CIR, RRM, and NRM—highlight serotherapy as a preliminary determinant of relapse risk, with potential relevance beyond this platform.

The cohort included pediatric and adult cases, MD and MMD donor settings, and both myeloid and lymphoblastic malignancies, representing the most comprehensive clinical evaluation of post-HCT CIK therapy to date.^16,17,20,21,30,31^ Despite this heterogeneity, IL15-CIK was administered under a harmonized protocol, enabling combined analyses across cohorts, and the differences in OS between the PS and CT cohorts are likely confounded by a coincidental higher proportion of heavily pretreated patients, multiple previous HCTs, and refractory disease associated with elevated NRM and RRM in the PS cohort.

Preemptive DLI, the closest clinical comparator, can reverse impending relapse and promote donor chimerism,^32^ yet its survival benefit remains uncertain because of the lack of prospective comparative trials^12^ and is offset by substantial toxicity. By contrast, IL15-CIK demonstrated a distinctly favorable safety profile. CRS was rare, and neutropenia linked to reduced efficacy—seen after CAR-T-cell therapy or DLI—was not observed.^33-35^ Although the incidence of grade 2-3 aGVHD (18%) and cGVHD (7%) was slightly higher than that in early IL2-CIK trials, it remained substantially lower than that reported after DLI (aGVHD: 28%-42%, cGVHD: up to 59%).^12,16,17,20,21^ Manageable GVHD not meeting DLT criteria after in vitro T-cell depletion supports the future extension of IL15-CIK to post-transplant cyclophosphamide-based MMD platforms, which are currently used in practice for DLI in adults.^36^

AEs directly attributed to IL15-CIK were infrequent and mild, consistent with previous clinical trials of allogeneic CIK therapy.^16-21,25,26^ SAEs did not exceed the expected toxicity burden associated with HCT alone.^22^ Importantly, NRM occurred exclusively in heavily pretreated patients with poor performance status and no clear association with IL15-CIK. Together, these findings indicate that donor-derived IL15-CIK can be administered safely without unexpected or excessive toxicity.

Beyond safety, efficacy outcomes further support the clinical relevance of IL15-CIK. Disease clearance, as indicated by CI-CMR rates of 74% and 13% at day 700 in the preemptive and salvage cohorts, respectively, together with 5-year OS rates of 71%, 61%, and 20% in the consolidation, preemptive, and salvage cohorts, underscores the importance of preventive intervention. These outcomes compare favorably with published results of CAR-T therapy^30,37-39^ and DLI in adults. Consolidative treatment with modified DLI plus methotrexate achieved a 2-year OS of 69%,^40^ whereas cDLI resulted in a 76% 1-year PFS rate^41^ and preemptive DLI in AML resulted in a 64% 2-year OS.^42^ In the only pediatric DLI study to date, a 49% event-free survival at 2-4 years was reported.^9^ Notably, several immunotherapy approaches had previously failed in a subset of our cohort (DLI, n = 13; antibody-targeted therapy, n = 4; CAR-T, n = 1).

Consistent with the overall cohort composition, pediatric patients predominated. Notably, the consolidation cohort comprised exclusively pediatric cases (Data Supplement, Table S10), representing a heavily pretreated population with limited therapeutic options and historically poor outcomes after post-HCT relapse, as reflected by reported OS rates of 22% at 3 years,^43^ 24%-33% at 2-5 years after second HCT,^2,29,44^ and 15% at 5 years for second-relapse AML.^45^

In the absence of concurrent antileukemic therapy, observed outcomes are most likely attributable to IL15-CIK although residual effects of previous HCT cannot be excluded without random assignment. To inform future risk-adapted strategies, we explored predictors of response. Disease subtype and leukemic burden behaved as expected and consistent with previous DLI studies.^46^ Serotherapy type and timing emerged as influential factors: Campath-pretreated cases showed superior relapse prevention, whereas previous ATG exposure was associated with high CIR. These effects appeared largely independent of other clinical variables and are biologically plausible, given reports of impaired DLI efficacy in the presence of residual ATG.^47,48^ Although exploratory because of limited sample size and the absence of ATG level measurements, these findings warrant prospective validation.^49^

In summary, this study represents, to our knowledge, the largest clinical evaluation of IL15-CIK monotherapy to date and provides a strong rationale for further clinical investigation. We propose a prospective multicenter phase II trial in pAML, a population with an unmet clinical need, focusing on relapse prevention, using standardized dosing, harmonized deep response assessment, and integrated serotherapy monitoring, with additional doses administered for persistent or recurrent MRD according to current consensus recommendations.^50,51^ Given the rarity of eligible patients, a randomized comparison with DLI will not be feasible, and a multicenter approach will still be essential. Beyond clinical validation, future efforts will focus on refined patient stratification, identification of predictive biomarkers, and development of IL15-CIK as a platform for genetic enhancement (eg, chimeric antigen receptor engineering).^52-54^ Collectively, these steps aim to position IL15-CIK as a viable alternative to DLI and a versatile platform for targeted post-HCT cellular immunotherapy.

## Data Availability

A data sharing statement provided by the authors is available with this article at DOI https://doi.org/10.1200/JCO-25-01966.
