# Supplementary material for: First-in-Human Study of IL15–Activated Cytokine-Induced Killer Cells After Allogeneic HCT Shows Durable Remission and Serotherapy-Associated Immune Reconstitution in Leukemia
Source: J Clin Oncol. 2026 Apr 6;44(14):1323–36. doi: 10.1200/JCO-25-01966 (PMC13166077; doi:10.1200/JCO-25-01966)
Supplement: Supplementary file 2 [file jco-44-1323-s002.pdf]

First-in-Human Study of IL15–Activated Cytokine-Induced Killer Cells After Allogeneic HCT Shows Durable Remission and Serotherapy-Associated Immune Reconstitution in Leukemia

Eva Rettinger, MD[1]*; Dirk Heckl, PhD[1,2]; Martin Hutter[1]; Emilia Salzmann-Manrique[1]; Marie Luedtke[1]; Sabine Huenecke, PhD[1]; Melanie Bremm, PhD[1]; Claudia Cappel, PhD[1]; Gesine Bug, MD[3]; Johann Greil, MD[4]; Roland Meisel, MD[5]; Eva Maria Wagner-Drouet, MD[6]; Hubert Serve, MD[3]; Tayfun Güngör, MD[7]; Jan-Henning Klusmann, MD[1]; Thomas Klingebiel, MD[1]; Peter Bader, MD[1], and Halvard Bonig, MD[8,9]

[1]Department of Pediatrics, Goethe University Frankfurt, Germany
[2]Institute for Experimental Pediatric Hematology and Oncology (EPHO), Frankfurt, Germany
[3]Medical Clinical II, Goethe University Frankfurt, Germany
[4]University Children's Hospital, Heidelberg, Germany
[5]Division of Pediatric Stem Cell Therapy, Department of Pediatric Oncology, Hematology and Clinical Immunology, Medical Faculty, Heinrich-Heine-University, Duesseldorf, Germany
[6]Johannes Gutenberg-University Mainz, III. Department of Medicine, Germany
[7]Department of Pediatric Stem Cell Transplantation and Hematology, Children's Research Center, University Children's Hospital Zurich, Zurich, Switzerland
[8]Institute of Transfusion Medicine and Immunohematology, and German Red Cross Blood Center Frankfurt, Goethe University Frankfurt, Germany
[9]University of Washington, Department of Medicine, Division of Hematology, Seattle, WA, USA

Acknowledgements
The authors thank the LOEWE Center for Cell and Gene Therapy Frankfurt, funded by: Hessian Ministry of Higher Education, Research and the Arts, funding reference number: III L 4- 518/17.004 (2013), and the Else Kröner-Fresenius-Stiftung (P75/08//A62/08 and 2014_A305) for funding of this study. This work was supported by the Mildred Scheel Career Center (MSNZ) (70113301, ER), by grants from the Parents' Association "*Hilfe für Krebskranke Kinder e.V*." as part of the C[3]OMBAT consortium (ER, DH, ES-M, PB, JHK). DH is supported by the Frankfurt Foundation for Children with Cancer.

***Correspondence**
Eva Rettinger, Department of Pediatrics, Goethe University Frankfurt, Theodor-Stern-Kai 7, 60590 Frankfurt/Main, Germany; Phone: +49-69-6301-7542, Fax: +49-69-6301-4202, email: eva.rettinger@icloud.com

Running head: IL15-activated CIK Therapy After HCT in High-Risk Leukemia

The study has not been published elsewhere.

Disclosure of Conflicts of Interest
MH acknowledges a travel grant from Neovii Pharmaceuticals not pertinent to this piece of work. JHK acknowledges advisory roles with Pfizer, Boehringer, and Jazz Pharmaceuticals. PB declares research grants from Neovii, Riemser, medac GmbH (to Institution); advisory board for Novartis, Cellgene, Amgen, medac (personal and to Institution); Speakers Bureau of Miltenyi, Jazz, Riemser, Novartis and Amgen (to Institution). Lincensing fees and royalties from medac. HB acknowledges research support from Miltenyi, Sandoz-Hexal (a Novartis Company), honoraria and speakers' fees from BMS/Celgene, Miltenyi, Terumo BCT, consultancy and advisory roles with Apriligen, Arensia, Boehringer Ingelheim Vetmed, Byondis, Editas, medac, NMDP, Provirex, Sandoz-Hexal, royalties/licensing fees from medac and stocks from Healthineers within the last three years, none of which are pertinent to this piece of work.
All other authors declare no conflicts of interest.

Supplementary Methods and Data

Methods

Patient Cohort (Pre-screening)

Patients who received IL15-CIK from August 11, 2011, onward, were prospectively enrolled in a longitudinal observational PS. After initiation of the CT on March 24, 2016, consecutive cases were enrolled in an identically designed multicenter, prospective phase I/II CT (Eudra-CT: 2013-005446-11).

The relapse-prevention cohort included cases with <5% leukemic BM blasts at treatment initiation. This cohort was subdivided into (i) consolidation cases with unfavorable cytogenetic/molecular risk features or relapsed/refractory disease prior to HCT (**Table S10**), and (ii) preemptive cases with impending relapse, defined by reappearance of MRD (PCR-based), cytogenetic abnormalities, or declining donor chimerism after transplantation.

In lymphoblastic malignancies, PCR-based MRD and cytogenetic markers, when applicable, served as the primary indicators for both preemptive immune intervention and disease assessment during follow-up; chimerism analysis was used as a secondary or supportive marker. In myeloid disease, decreases in donor chimerism, defined as previously described by Rettinger and collegeues[1], were considered in combination with cytogenetic or molecular high-risk markers (when available). This approach was applied in both adult and pediatric patients. Patients in the preemptive cohort (n=34) were either MRD-, cytogenetic marker-, or molecular marker-positive, had developed mixed donor chimerism indicating impending relapse, or had previously experienced overt relapse after transplantation but were in CR at the time of the first infusion without having reached CMR (2 of 34 cases).

Cases presenting with ≥5% leukemic BM blasts at treatment initiation were classified as the salvage cohort. Of the 15 salvages cases, 8 received no antileukemia treatment before CIK, 3 received chemotherapy only, 3 received chemotherapy plus DLI, and 1 received DLI alone.

At baseline, the underlying disease status was categorized as CMR, CR/non-CMR, or overt relapse aligning with the consolidation, preemptive, and salvage cohorts (**Fig.S1A**).

Written informed consent for treatment and data collection was obtained from all participants before administration of the first IL15-CIK infusion.

Eligibility Criteria (Screening Phase)

Eligible cases included both children and adults (aged >1 year and <80 years) with HR hematological malignancies who had previously undergone HCT and required post-transplant intervention for which no alternative targeted therapy was available (**Fig.1**). Within this heavily pretreated cohort with high relapse risk and limited alternative therapeutic options, the enrolled patients are representative of the intended target population. The inclusion and exclusion criteria did not introduce a notable bias within this subgroup.

Treatment intent was determined by relapse risk, impending relapse, or overt relapse occurring more than 120 days post-transplant. For relapse-prevention intent, cases with <5% leukemic BM blasts within one week prior to treatment initiation were eligible; this included cases who had presented with overt relapse but achieved sufficient response after reinduction chemotherapy.

Additional key inclusion criteria for all cases were:

- Karnofsky or Lansky performance status ≥50%

- Absence of aGVHD grade ≥ 1 and absence of cGVHD
- No concurrent use of immunosuppressive, immunomodulatory medications, or corticosteroids for at least 3-4 weeks
- No exposure to another investigational anti-leukemic agent -except for tyrosine kinase inhibitors- within the preceding 28 days or five drug half-lives
- Absence of severe or active infections

IL15-CIK Treatment Protocol (Treatment Phase)

Patients received IL15-CIK either under existing market authorization in the PS or through participation in the CT (**Fig. 1**).

Initial Dose: The starting T-cell dose of IL15-CIK was standardized at 1.0×10^6/kg, regardless of donor type or recipient age.

Individualized Dose Escalation: Subsequent dose escalations were guided by the patients' risk and clinical condition – specifically, the presence of MRD, cytogenetic abnormalities, or declining donor chimerism and the absence of new or deteriorating aGVHD. Escalation was permitted for both pediatric and adult cases, irrespective of donor type. The T-cell escalation schema was as follows:

- 5.0×10^6/kg per infusion
- 1.0×10^7/kg per infusion
- Up to a maximum dose of 1.0×10^8/kg per infusion (after consultation with the study team).

Dose escalation followed a stepwise approach, starting at 1×10^6, 5×10^6, and 1×10^7 T-cells/kg body weight per infusion in the clinical trial (CT) cohort, and up to a maximum of 1×10^8 T-cells/kg per infusion in the pilot study cohort. The dosing strategy also included a provision that dose escalation was permitted only after a mandatory

observation interval of 4–6 weeks between consecutive infusions. Dose escalation could, under certain circumstances, be determined by the individual assessment of patient safety and relapse risk: Treating physicians could discuss individual cases with the study team, and dose escalation was left to their discretion to ensure patient safety, resulting in more conservative dosing in MMD patients. Additional infusions at higher dose levels were allowed for the treatment of persistent or impending relapse after HCT, provided that no aGVHD higher than grade 1, treatment-related adverse events, or other contraindications had occurred. In one case without GVHD or other drug-related AEs, this approach resulted in escalation up to 1×10^8 T-cells/kg per infusion within the CT cohort.

Treatment Schedule: IL15-CIK were administered at intervals of 4–6 weeks, without the use of preconditioning, lymphodepletion, GVHD prophylaxis or concomitant antileukemic therapy.

Discontinuation Criteria / End of Study: Therapy was discontinued upon achievement of CMR, completion of one year of treatment, or after a total of eight infusions – whichever occurred first. Additionally, treatment was halted in cases of severe infection, any DLT, and particularly the development of aGVHD exceeding grade 1. Cases who responded but experienced reappearance of disease during the 6-month follow-up period could receive additional IL15-CIK infusions.

DLTs were evaluated on a per-patient basis. DLTs were defined as grade 3-4 aGVHD, extensive cGVHD, or unmanageable or permanently harmful toxicity.

IL15-CIK infusions in an individual case were administered only when acute GVHD > grade 1, any evidence of chronic GVHD, unmanageable active infection or organ toxicity, or a decline in clinical performance index below 50% had been confidently

excluded (DLT criteria in or setting). All patients underwent at weekly safety monitoring for potential adverse events. A mandatory safety interval of 4–6 weeks between infusions was required, and dose escalation was allowed only if no toxicity meeting DLT criteria occurred. In the event of any DLT, further infusions were withheld.

IL15-CIK as an Approved Multifunctional DLI

Our center is the only facility in Germany that holds both the required manufacturing license and the national marketing authorization, allowing us to offer IL15-CIK as a therapeutic option for the approved indication[2].

Since August 11, 2011, our product has received individual approvals for compassionate use from the local regulatory authority (Regierungspräsidium Darmstadt, Germany). Subsequently, on June 3, 2014, the Paul-Ehrlich-Institute (PEI) authorized the IL15-CIK product (PEI A.11630.01.1) under the Hospital Exemption regulation for ATMPs as specified in Section 4b, Paragraph 3, German Medicinal Products Act (AMG).

The generation of IL15-CIK was previously reported in some detail. Briefly, manufacturing follows current Good Manufacturing Practice (cGMP) standards under the pharmaceutical responsibility of German Red Cross Blood Donor Service Baden-Württemberg-Hesse in Frankfurt, Germany[2].

Immune Monitoring and Functional Immune Assessment

A comprehensive immune monitoring strategy was employed to assess the immunomodulatory effects and immune responses induced by IL15-CIK. Weekly flow cytometric analyses were performed to characterize modulation of immune cell subpopulations[3]. Supernatants collected prior to treatment and at 1 hour, 1 week, and 2–4 weeks after IL15-CIK infusion were analyzed using the Multi-Analyte Flow Assay

Kit LEGENDplex™ (Human CS8/NK Panel, 13-plex, Cat. No. 740267; BioLegend, San Diego, USA). Fluorescent signals were acquired on a BD FACS Canto™ flow cytometer, and analyte concentrations were calculated using LEGENDplex™ Data Analysis Software (Version 7.0; VigeneTech, Carlisle, USA).

Primary Study Endpoints

Feasibility: Key factors included cell collection, *ex vivo* manufacturing time, quality control, product release, regulatory compliance, and donor-related variables. Feasibility was assessed by evaluating the percentage of IL15-CIK products manufactured and released relative to required amounts in the preemptive setting.

Safety: Safety assessments were performed before and during each infusion, for 24 hours afterward, weekly during treatment, and every 3 months for 6 months of follow-up. Monitoring included vital signs, physical examinations, laboratory values, AEs, GVHD, CRS, immune effector cell-associated neurotoxicity syndrome (ICANS), acute kidney and liver toxicities, and hematotoxicities, enabling detection of any DLTs associated with IL15-CIK.

Secondary Study Endpoints

Clinical outcome at day 100 was defined as survival in CMR, CR, or relapse, or death due to RRM or NRM within treatment-intent cohorts (consolidation, preemptive, salvage). In preemptive and salvage cohorts, CI-CMR was assessed at day 400/700.

Endpoints included OS, PFS, CIR, CI-CMR and NRM, all starting from the first IL15-CIK infusion. OS was defined as the time to death from any cause. PFS was defined as the time to disease progression or death from any cause, whichever occurred first, CIR as the time to relapse with NRM as a competing event. CI-CMR as the time to achieving CMR with relapse, NRM and RRM as competing events, and NRM as death

from any cause without relapse. For PFS, CIR and NRM patients were censored at the date of consecutive HCT, which was the case for two preemptive patients developing secondary MDS followed by HCT at day 260 and day 615 after first CIK infusion. For all other patients, censoring was due to a loss of follow up with these patients being alive at the last date of follow up (for OS) and no change in disease status, with the exception of four patients noted in the main text (loss of follow up after relapse, RRM). For survival analyses, numeric covariates were transformed using a base 10-logarithm.

Statistical Analysis

Statistical analyses were performed using R. Demographic and clinical characteristics, as well as T-cell dose data were summarized descriptively. Group comparisons were performed using Fisher's exact test, Pearson's chi-square test, Wilcoxon rank-sum test, or Kruskal-Wallis rank-sum test. Median follow-up was estimated using the reverse Kaplan–Meier method. Probabilities for OS and PFS were estimated using Kaplan–Meier and compared using log-rank tests. Cumulative incidence was used to estimate CIR, CI-CMR and NRM. The univariable analysis of cumulative incidences was carried out with Gray's test. Patients in the salvage setting (i.e., already in relapse) were excluded from PFS and CIR analyses. Consolidation cases (i.e. already in CMR) were excluded from CI of CRM analysis. Time-dependent Cox regression evaluated associations between aGVHD and outcomes. A multivariable Cox regression model was fitted for OS. A semiparametric Fine-Gray competing risk model was utilized for the multivariable analysis of CIR. Variable selection used backward elimination with a removal threshold of $p > 0.05$. Mixed-effects models with patient-level random intercepts evaluated longitudinal trajectories of leukocyte subpopulations using cubic B-splines (internal knots at the 33rd and 67th percentiles). The paired Wilcoxon test compared leukocyte counts before and 1 week after IL15-CIK infusion. Cytokine levels

at baseline, at 1 hour, 1 week, and 2–4 weeks after infusion were analyzed using the Friedman test. Percentages are rounded and may not total 100%. All tests were two-sided with $\alpha = 0.05$ and 95% CIs.

Supplementary References

1. Rettinger E, Willasch AM, Kreyenberg H, et al: Preemptive immunotherapy in childhood acute myeloid leukemia for patients showing evidence of mixed chimerism after allogeneic stem cell transplantation. Blood 118:5681-8, 2011
2. Bremm M, Pfeffermann LM, Cappel C, et al: Improving Clinical Manufacturing of IL-15 Activated Cytokine-Induced Killer (CIK) Cells. Front Immunol 10:1218, 2019
3. Rettinger E, Huenecke S, Bonig H, et al: Interleukin-15-activated cytokine-induced killer cells may sustain remission in leukemia patients after allogeneic stem cell transplantation: feasibility, safety and first insights on efficacy. Haematologica 101:e153-6, 2016

Supplementary Figure Legends

Supplementary Figure 1. Schematic Overview of Outcomes Defining Survival

A total of 53 patients received IL15-CIK. Of these, 3 patients underwent a subsequent HCT ($^{+}$) followed again by IL15-CIK derived from a different donor, resulting in a total of 56 treatment cases administered across the 53 patients. Most cases were aligned to the preemptive (n=34), followed by the salvage (n=15), and consolidation cohorts (n=7) (**A**). The survival outcomes (*) were evaluated only for the final therapy period, which included patients who were alive in complete remission (CR), and those who died either due to relapse-related (RRM), or relapse-unrelated mortality (NRM) while in complete molecular remission (**B**). Among the 53 patients, 27 were alive, and 26 died: 6 patients died due to NRM, 16 died due to relapse (RRM) and 4 additional patients died due to RRM and/or NRM after undergoing a subsequent HCT. The median follow-up for the entire cohort was 7.3 years (range, 1.2–11.7 years).

Supplementary Figure 2. IL15-CIK dosing and aGVHD development

Neither maximum IL15-CIK dose (MD, p=0.158; MMD, p=0.494) (**A**) nor cumulative IL15-CIK dose (MD, p=0.278; MMD, p=0.429) (**B**) were significantly different between patients not developing acute graft-versus-host disease (aGVHD), developing grade 1 aGVHD, developing grade 2 aGVHD, or developing grade 3 aGVHD.

Each case was evaluated once with the highest grade of GVHD per case. Maximum dose and cumulative dose refer to the T-cell-equivalent dosing before developing the respective grade GVHD. (MD, matched donor; MMD, mismatched donor).

Supplementary Figure 3. Predictors of Outcome at Day 100 After First IL15-CIK

Univariable analyses evaluating the association between clinical and transplant-related variables and treatment outcome at day 100 showed that neither disease type (myeloid vs. lymphoblastic), patient age, number of prior HCTs, donor type (HLA-matched vs. HLA-mismatched), graft source (bone marrow (BM) vs. peripheral blood stem cells (PBSC)), nor graft content ($CD34^+$, $CD3^+$) or *ex vivo* graft manipulation were significantly associated with outcome. In contrast, remission status at HCT (**p=0.041**), time from HCT to immune intervention (**p=0.023**), and serotherapy type (**p=0.004**) were significantly associated with day 100 outcome in univariable analyses. These findings suggest a potential association between prior serotherapy choice and IL15-CIK efficacy, although this may also be influenced by donor-, graft-, or conditioning-related variables.

Supplementary Figure 4. Outcome and Treatment Response in Pediatric AML

Apparent superior outcome benefit at day 100 for the pediatric AML subgroup (n=19) is shown (**A**), with cumulative incidence of complete molecular remission (CI-CMR) assessed at day 400 (**B**). CI-CMR rates were analyzed for cases treated in the preemptive (impending relapse) and salvage (overt relapse) settings.

Supplementary Figure 5. Immune Cell Dynamics Following IL15-CIK

Panels (**A–C**) illustrate enhanced reconstitution of T-cell and NK cell compartments during the first week post-infusion compared with pre-infusion levels, while panel (**D**) shows expansion of monocytes – despite their absence in the IL15-CIK products – suggesting an immunomodulatory effect induced by IL15-CIK. The data represent matched-pair analyses comparing immune profiles before and one week after IL15-CIK.

Supplementary Figure 6. Longitudinal Cytokine Profiling

Longitudinal cytokine profiling demonstrated moderate decreases in IL-2 one week after IL15-CIK compared with pre-infusion levels, consistent with a Th1-skewed immune response (**A**). Elevated levels of FAS, and FASL suggest activation of non-apoptotic cytotoxic pathways, while perforin, granzyme A, and granulysin reflect granule-mediated apoptosis. Peripheral blood cytokine concentrations did not consistently mirror ongoing immune activity within leukemic niches (**B**).

Supplementary Figure 7. Survival in Cohorts with Relapse-Prevention Intent

The 5-year PFS, CIR, and NRM for the combined consolidation and preemptive cohort (relapse-prevention intent) are shown in panels **A-C**. In the corresponding pediatric AML subgroup, 5-year PFS, CIR, and NRM were 69%, 15%, and 15%, respectively. Although direct comparison with the entire relapse-prevention-intent cohort (indicated by an asterisk; all patients*) was not feasible, this figure shows a subgroup with substantial unmet clinical need and potentially high benefit from IL15-CIK therapy. The risk tables provide the numbers of censored patients in brackets.

Supplementary Figure 8. Survival according to Disease and Age

Progression-free survival (PFS), the cumulative incidence of relapse (CIR) and non-relapse mortality (NRM) at 5 years by disease (left column) and age group (right column) are shown. Subgroup analyses suggested higher PFS and lower CIR in myeloid compared with lymphoblastic disease, while NRM was comparable (**Fig.S8**, left column). Although limited by small patient numbers, adults appeared to have more favorable PFS and CIR than pediatric cases (**Fig.S8**, right column). The risk tables provide the numbers of censored patients in brackets.

Supplementary Figure 9. Survival of the Clinical Trial (CT) and the Pilot Study (PS) Cohorts

Across treatment cohorts, overall survival (OS) was significantly better in the CT group compared to the PS cohort (***p*****=0.041**) (**A**), whereas progression-free survival (PFS) (**B**), and cumulative incidence of relapse (CIR) (**C**), and NRM (**D**) were comparable between the two groups. The risk tables provide the numbers of censored patients in brackets.

Supplementary Figure 10. Mutational Landscape and High-Risk Cases

Genetic mutations identified in the myeloid (**A**) and lymphoblastic (**B**) cases included several alterations that defined high-risk features across cohorts. Day 100 outcomes associated with these mutations are illustrated for the myeloid (**C**) and lymphoblastic (**D**) cohorts.

Figure S1A

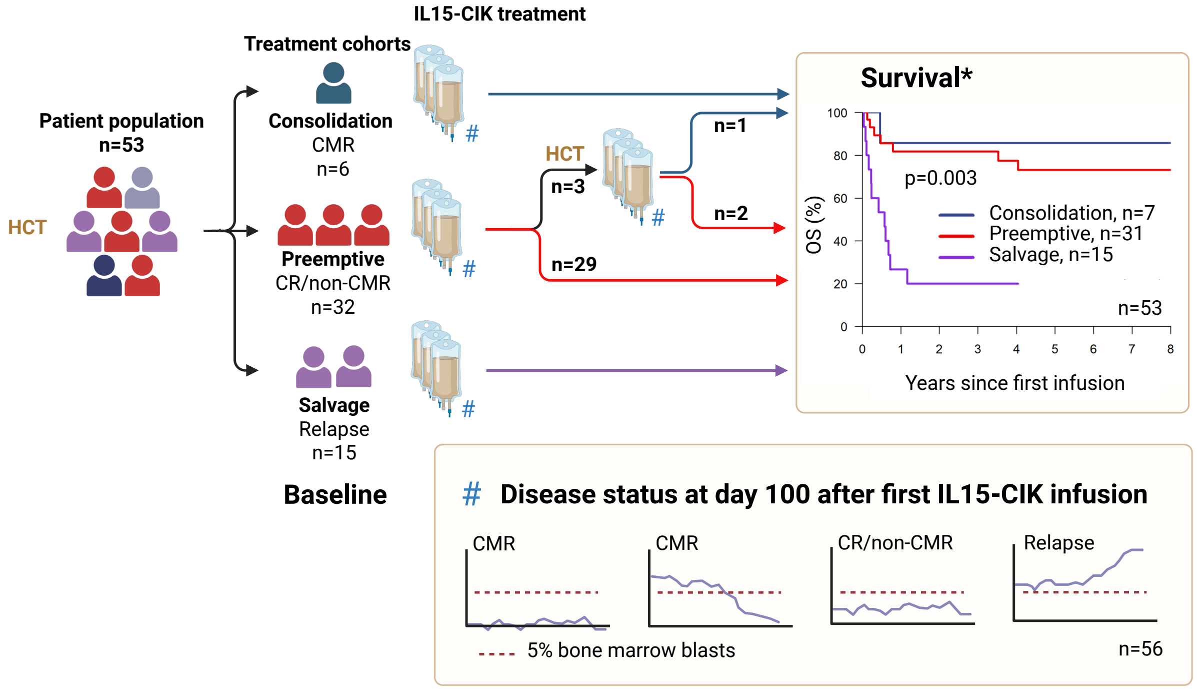

Figure S1B

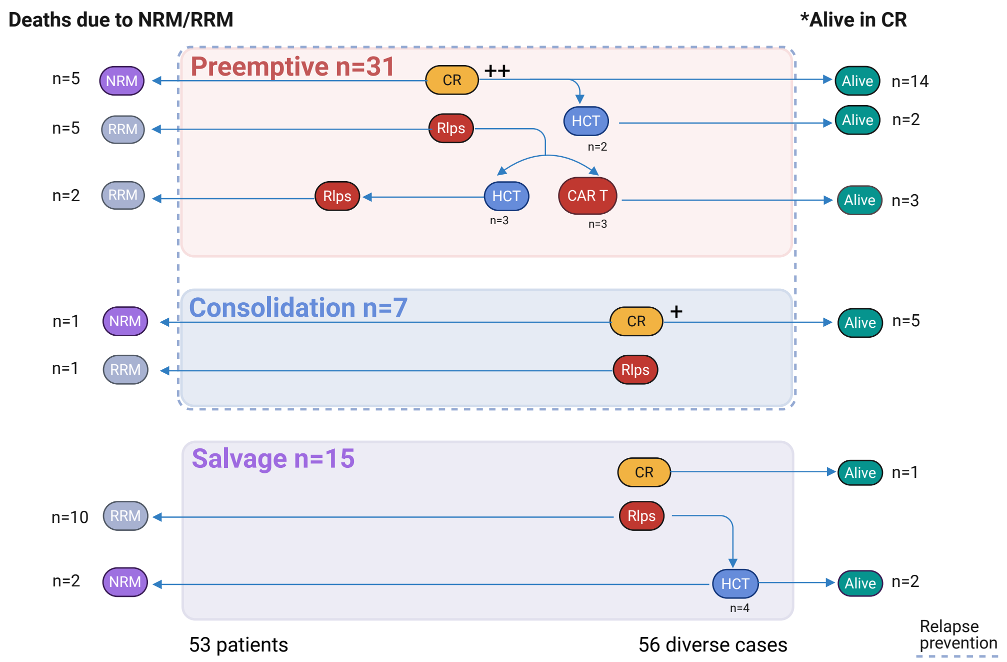

Figure S2

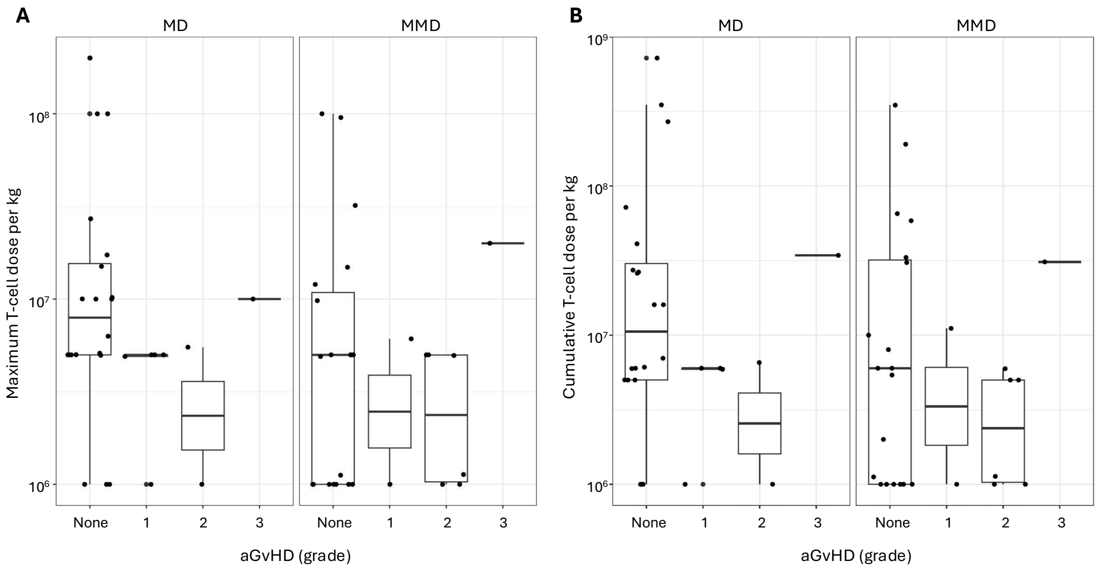

A
MD
MMD
Maximum T-cell dose per kg
10^8
10^7
10^6
None
1
2
3
aGvHD (grade)
B
Cumulative T-cell dose per kg
10^9

Figure S3

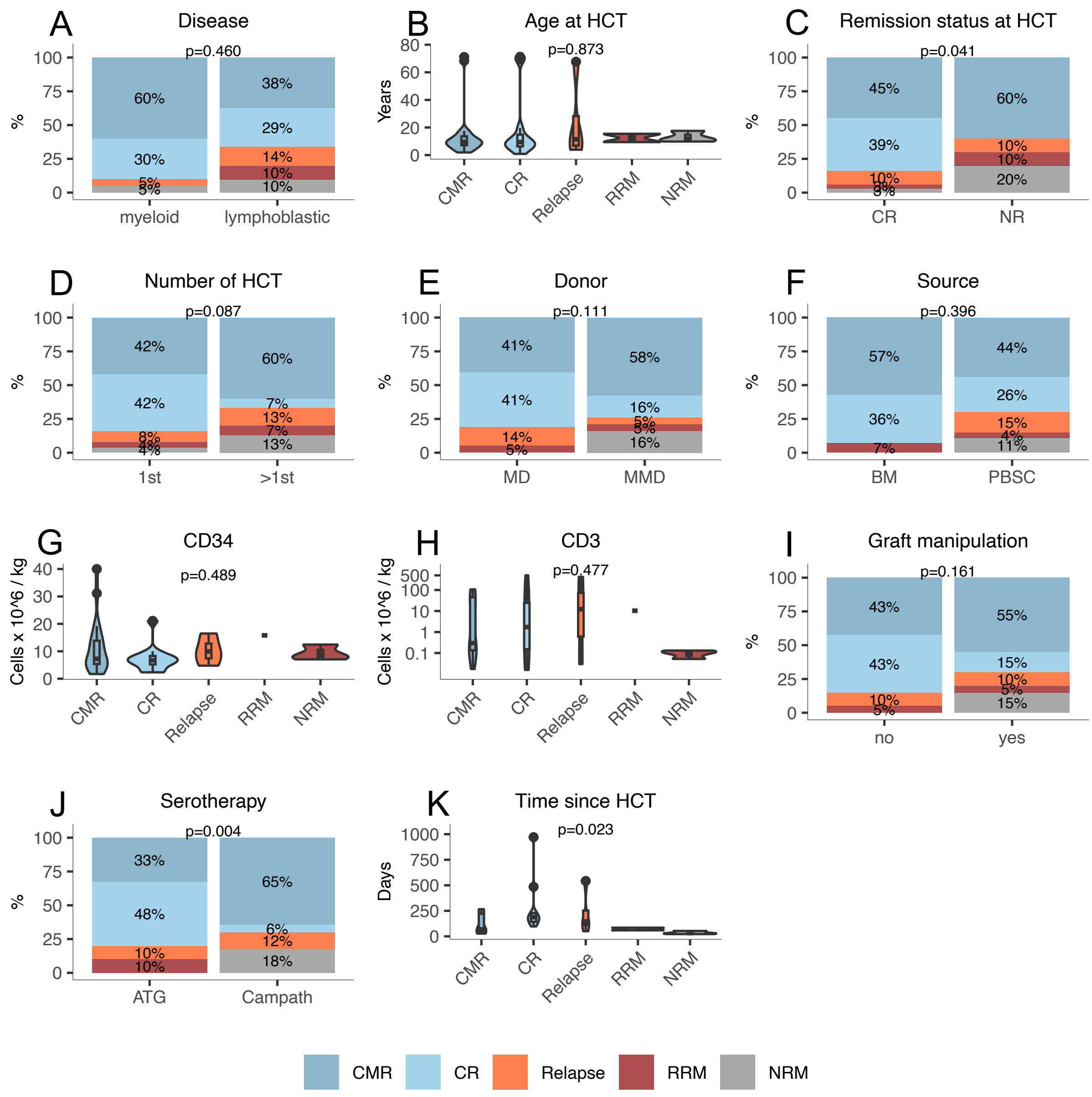

A Disease p=0.460
myeloid 60% 30% 5% 5%
lymphoblastic 38% 29% 14% 10% 10%
B Age at HCT p=0.873
Years
C Remission status at HCT p=0.041
CR 45% 39% 10% 3% 3%
NR 60% 10% 10% 20%
D Number of HCT p=0.087
1st 42% 42% 8% 4% 4%
>1st 60% 7% 13% 7% 13%
E Donor p=0.111
MD 41% 41% 14% 5%
MMD 58% 16% 5% 5% 16%
F Source p=0.396
BM 57% 36% 7%
PBSC 44% 26% 15% 4% 11%
G CD34 p=0.489
Cells x 10^6 / kg
H CD3 p=0.477
Cells x 10^6 / kg
I Graft manipulation p=0.161
no 43% 43% 10% 5%
yes 55% 15% 10% 5% 15%
J Serotherapy p=0.004
ATG 33% 48% 10% 10%
Campath 65% 6% 12% 18%
K Time since HCT p=0.023
Days
CMR CR Relapse RRM NRM

Figure S4

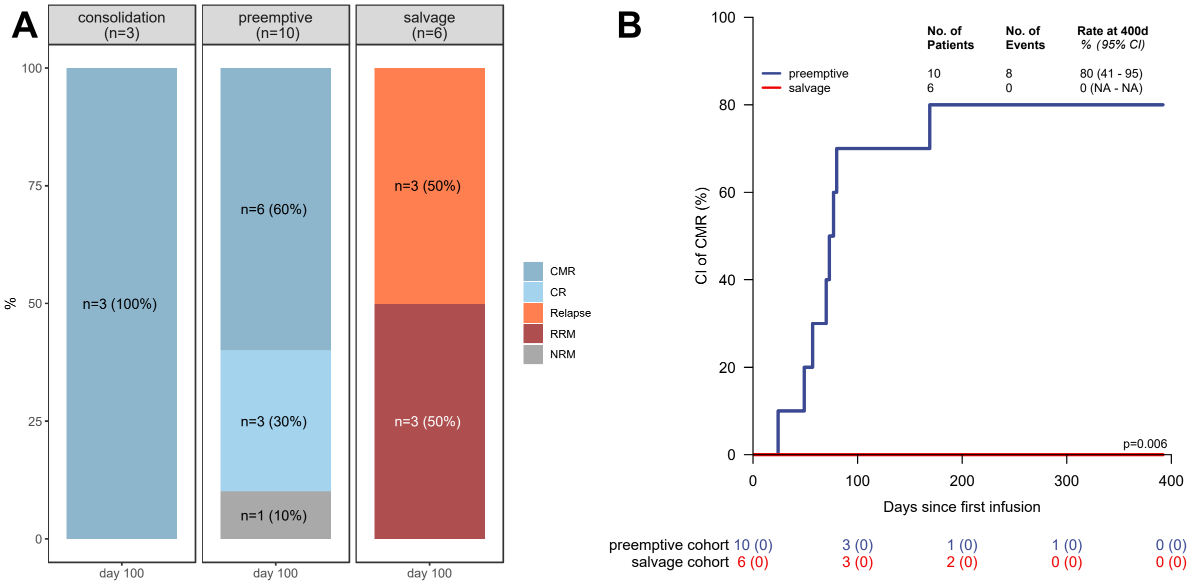
A
consolidation (n=3)
preemptive (n=10)
salvage (n=6)
%
100
75
50
25
0
n=3 (100%)
n=6 (60%)
n=3 (30%)
n=1 (10%)
n=3 (50%)
n=3 (50%)
day 100
day 100
day 100
CMR
CR
Relapse
RRM
NRM
B
CI of CMR (%)
No. of Patients
No. of Events
Rate at 400d % (95% CI)
preemptive 10 8 80 (41 - 95)
salvage 6 0 0 (NA - NA)
p=0.006
0
100
200
300
400
Days since first infusion
preemptive cohort 10 (0) 3 (0) 1 (0) 1 (0) 0 (0)
salvage cohort 6 (0) 3 (0) 2 (0) 0 (0) 0 (0)

Figure S5

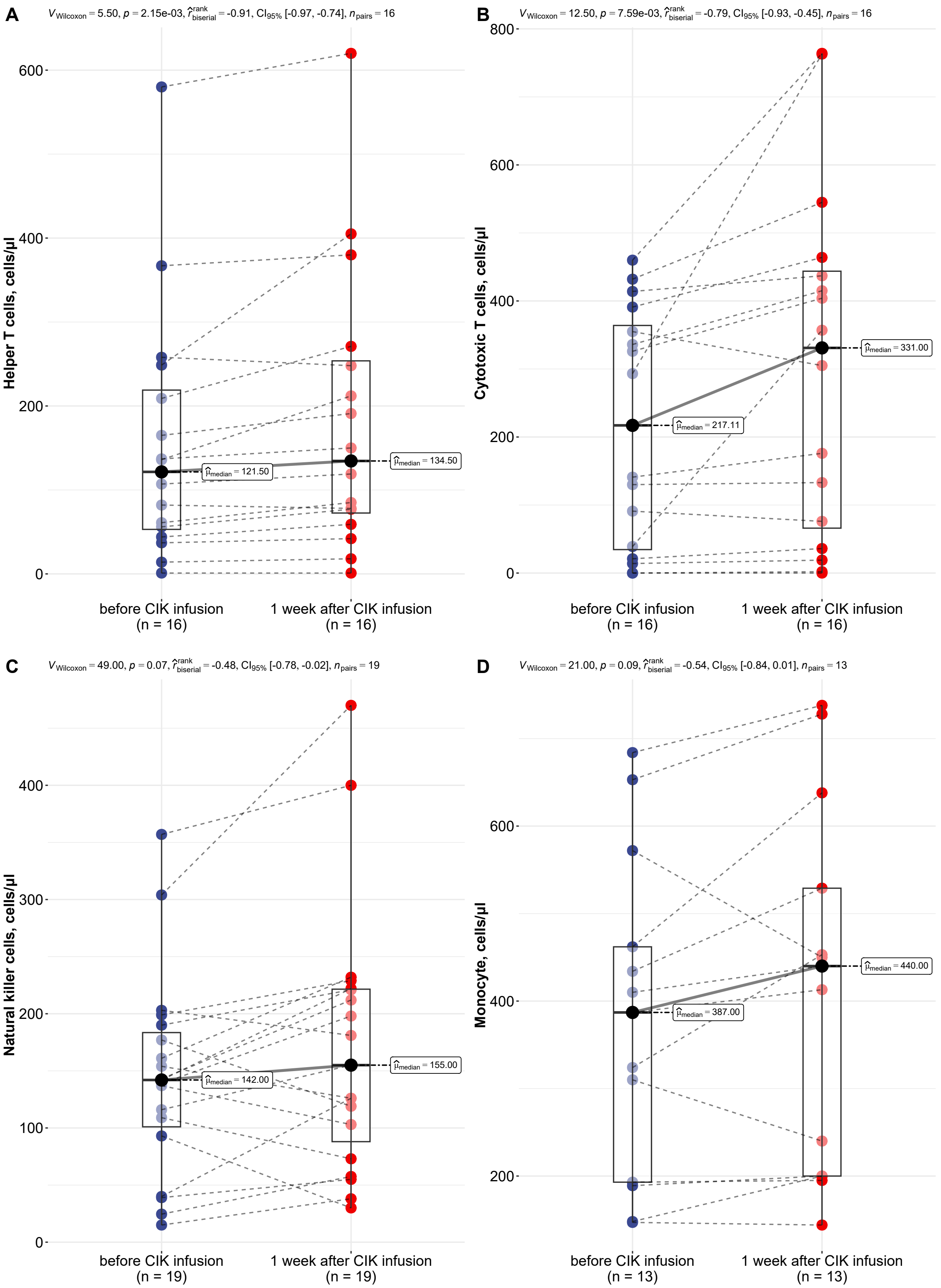
A
$V_{Wilcoxon}$ = 5.50, p = 2.15e-03, $\hat{r}^{rank}_{biserial}$ = -0.91, $CI_{95\%}$ [-0.97, -0.74], n_{pairs} = 16
Helper T cells, cells/µl
$\hat{\mu}_{median}$ = 121.50
$\hat{\mu}_{median}$ = 134.50
before CIK infusion (n = 16)
1 week after CIK infusion (n = 16)
B
$V_{Wilcoxon}$ = 12.50, p = 7.59e-03, $\hat{r}^{rank}_{biserial}$ = -0.79, $CI_{95\%}$ [-0.93, -0.45], n_{pairs} = 16
Cytotoxic T cells, cells/µl
$\hat{\mu}_{median}$ = 217.11
$\hat{\mu}_{median}$ = 331.00
before CIK infusion (n = 16)
1 week after CIK infusion (n = 16)
C
$V_{Wilcoxon}$ = 49.00, p = 0.07, $\hat{r}^{rank}_{biserial}$ = -0.48, $CI_{95\%}$ [-0.78, -0.02], n_{pairs} = 19
Natural killer cells, cells/µl
$\hat{\mu}_{median}$ = 142.00
$\hat{\mu}_{median}$ = 155.00
before CIK infusion (n = 19)
1 week after CIK infusion (n = 19)
D
$V_{Wilcoxon}$ = 21.00, p = 0.09, $\hat{r}^{rank}_{biserial}$ = -0.54, $CI_{95\%}$ [-0.84, 0.01], n_{pairs} = 13
Monocyte, cells/µl
$\hat{\mu}_{median}$ = 387.00
$\hat{\mu}_{median}$ = 440.00
before CIK infusion (n = 13)
1 week after CIK infusion (n = 13)

Figure S6

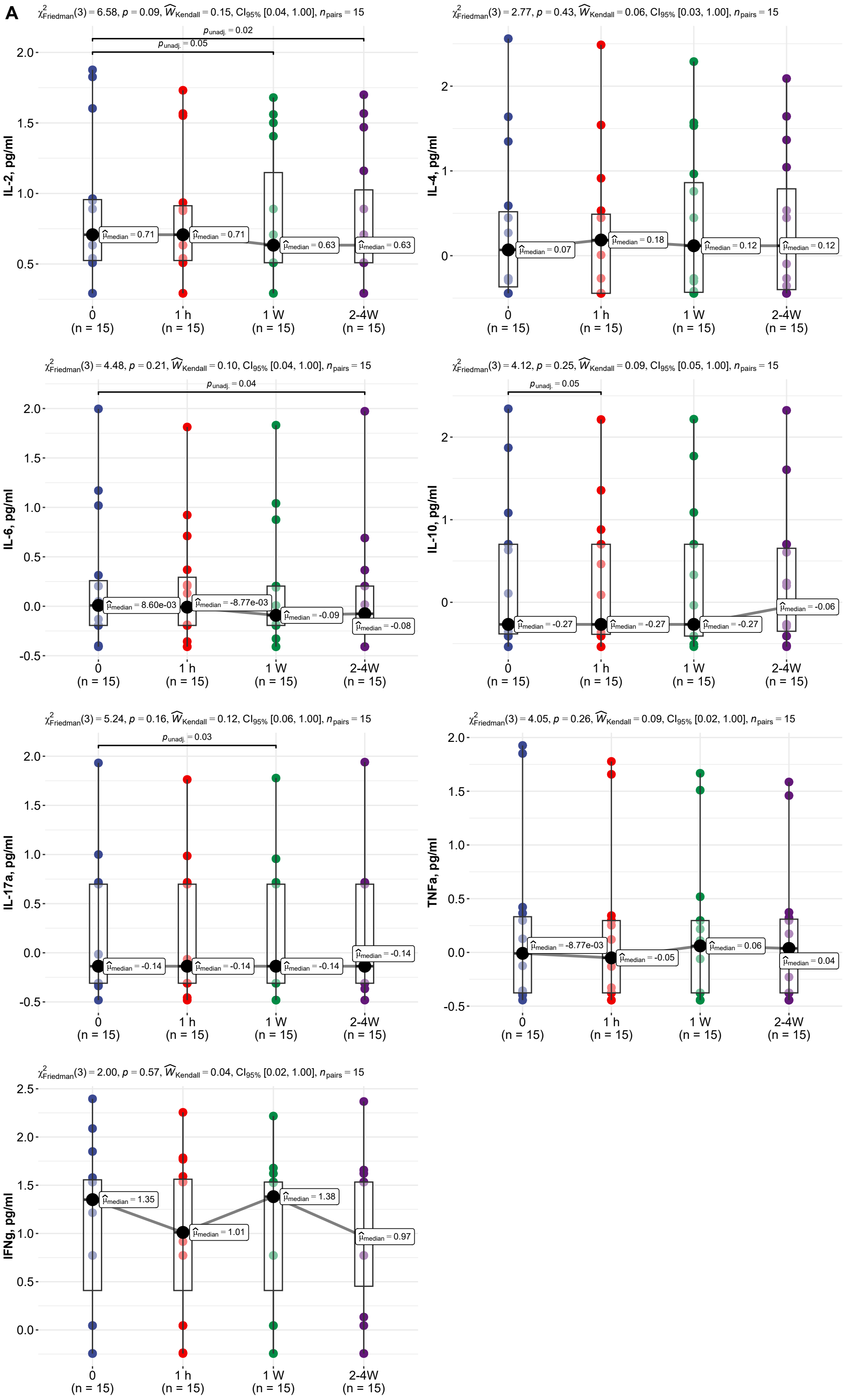

A
IL-2, pg/ml
IL-4, pg/ml
IL-6, pg/ml
IL-10, pg/ml
IL-17a, pg/ml
TNFa, pg/ml
IFNg, pg/ml
0
1 h
1 W
2-4W
(n = 15)

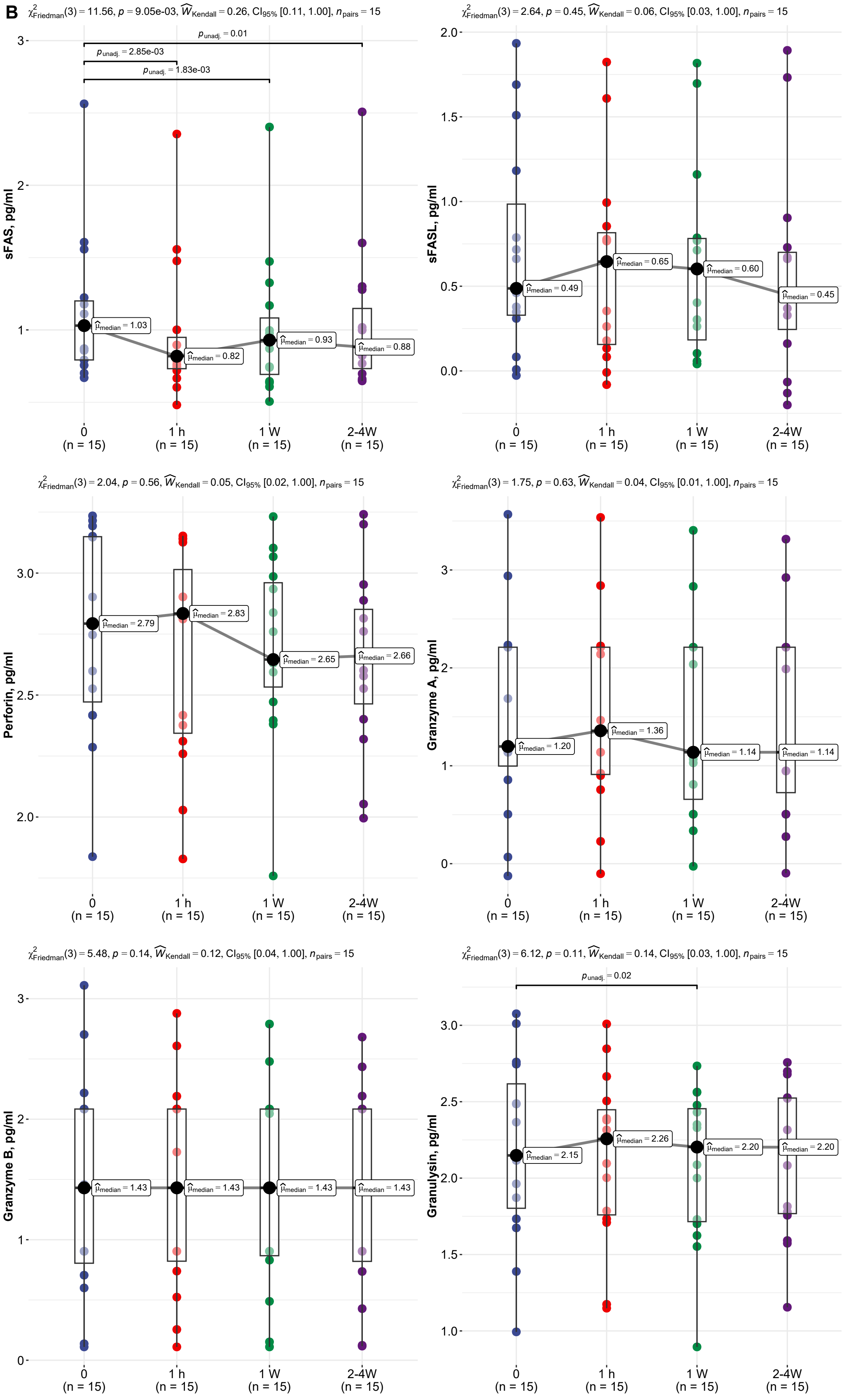
B
χ²Friedman(3) = 11.56, p = 9.05e-03, Ŵ Kendall = 0.26, CI95% [0.11, 1.00], npairs = 15
punadj. = 0.01
punadj. = 2.85e-03
punadj. = 1.83e-03
sFAS, pg/ml
μ̂median = 1.03
μ̂median = 0.82
μ̂median = 0.93
μ̂median = 0.88
χ²Friedman(3) = 2.64, p = 0.45, Ŵ Kendall = 0.06, CI95% [0.03, 1.00], npairs = 15
sFASL, pg/ml
μ̂median = 0.49
μ̂median = 0.65
μ̂median = 0.60
μ̂median = 0.45
χ²Friedman(3) = 2.04, p = 0.56, Ŵ Kendall = 0.05, CI95% [0.02, 1.00], npairs = 15
Perforin, pg/ml
μ̂median = 2.79
μ̂median = 2.83
μ̂median = 2.65
μ̂median = 2.66
χ²Friedman(3) = 1.75, p = 0.63, Ŵ Kendall = 0.04, CI95% [0.01, 1.00], npairs = 15
Granzyme A, pg/ml
μ̂median = 1.20
μ̂median = 1.36
μ̂median = 1.14
μ̂median = 1.14
χ²Friedman(3) = 5.48, p = 0.14, Ŵ Kendall = 0.12, CI95% [0.04, 1.00], npairs = 15
Granzyme B, pg/ml
μ̂median = 1.43
μ̂median = 1.43
μ̂median = 1.43
μ̂median = 1.43
χ²Friedman(3) = 6.12, p = 0.11, Ŵ Kendall = 0.14, CI95% [0.03, 1.00], npairs = 15
punadj. = 0.02
Granulysin, pg/ml
μ̂median = 2.15
μ̂median = 2.26
μ̂median = 2.20
μ̂median = 2.20
0
(n = 15)
1 h
(n = 15)
1 W
(n = 15)
2-4W
(n = 15)

Figure S7

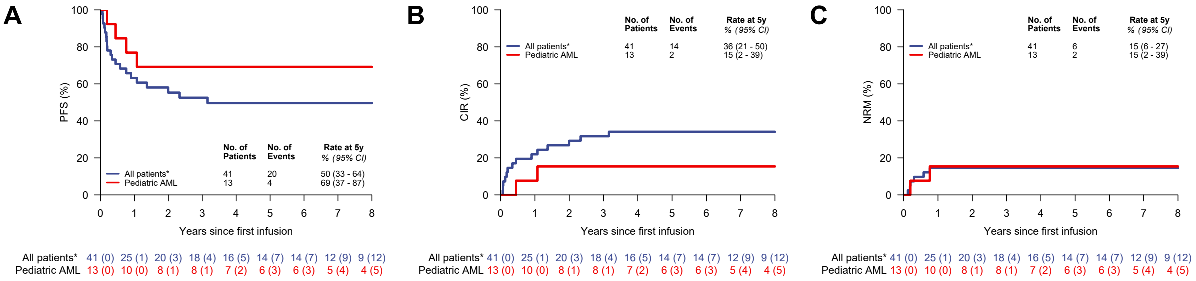
A
PFS (%)
Years since first infusion
No. of Patients
No. of Events
Rate at 5y % (95% CI)
All patients* 41 20 50 (33 - 64)
Pediatric AML 13 4 69 (37 - 87)
All patients* 41 (0) 25 (1) 20 (3) 18 (4) 16 (5) 14 (7) 14 (7) 12 (9) 9 (12)
Pediatric AML 13 (0) 10 (0) 8 (1) 8 (1) 7 (2) 6 (3) 6 (3) 5 (4) 4 (5)
B
CIR (%)
Years since first infusion
No. of Patients
No. of Events
Rate at 5y % (95% CI)
All patients* 41 14 36 (21 - 50)
Pediatric AML 13 2 15 (2 - 39)
All patients* 41 (0) 25 (1) 20 (3) 18 (4) 16 (5) 14 (7) 14 (7) 12 (9) 9 (12)
Pediatric AML 13 (0) 10 (0) 8 (1) 8 (1) 7 (2) 6 (3) 6 (3) 5 (4) 4 (5)
C
NRM (%)
Years since first infusion
No. of Patients
No. of Events
Rate at 5y % (95% CI)
All patients* 41 6 15 (6 - 27)
Pediatric AML 13 2 15 (2 - 39)
All patients* 41 (0) 25 (1) 20 (3) 18 (4) 16 (5) 14 (7) 14 (7) 12 (9) 9 (12)
Pediatric AML 13 (0) 10 (0) 8 (1) 8 (1) 7 (2) 6 (3) 6 (3) 5 (4) 4 (5)

Figure S8

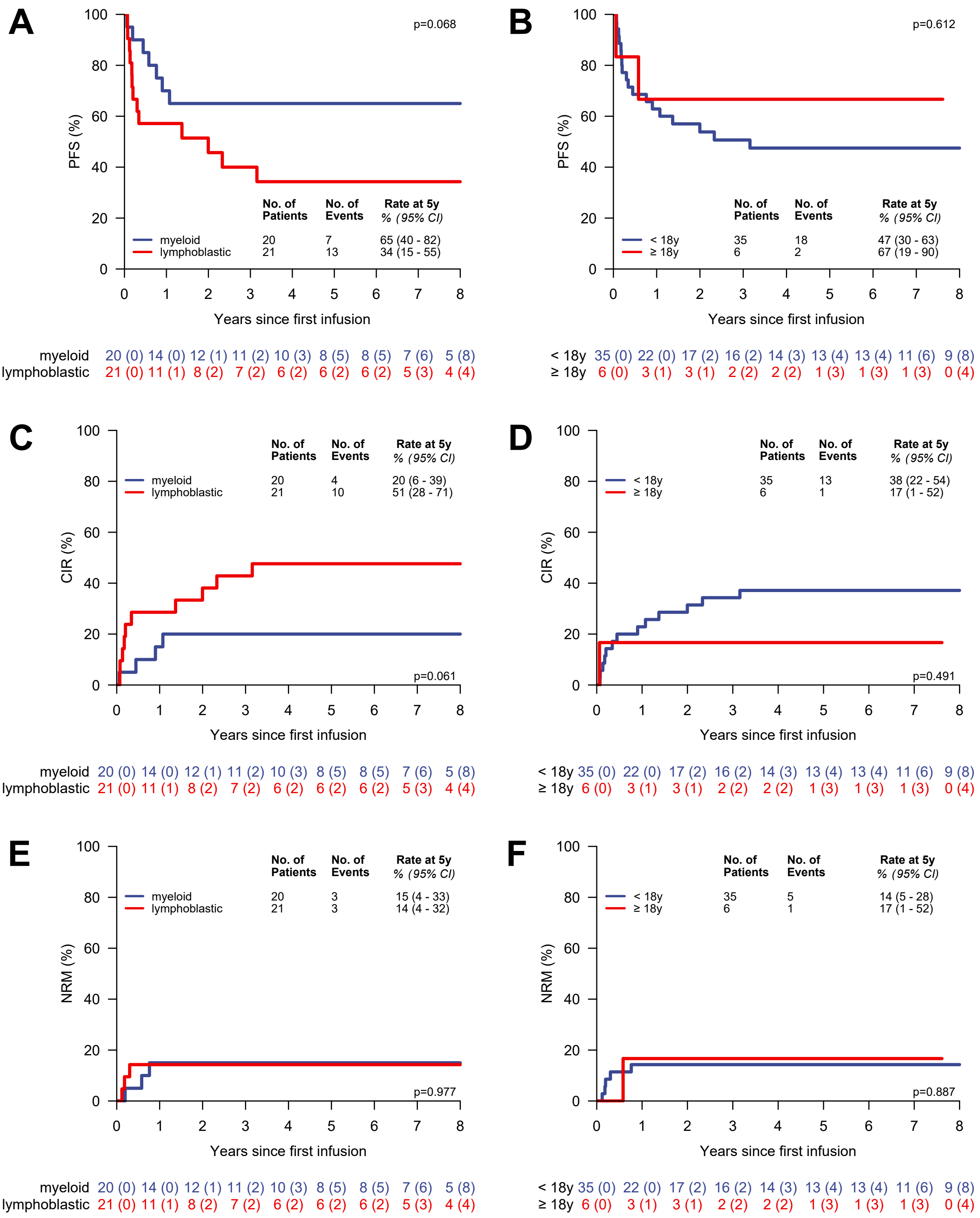
A
p=0.068
PFS (%)
Years since first infusion
No. of Patients
No. of Events
Rate at 5y % (95% CI)
myeloid 20 7 65 (40 - 82)
lymphoblastic 21 13 34 (15 - 55)
myeloid 20 (0) 14 (0) 12 (1) 11 (2) 10 (3) 8 (5) 8 (5) 7 (6) 5 (8)
lymphoblastic 21 (0) 11 (1) 8 (2) 7 (2) 6 (2) 6 (2) 6 (2) 5 (3) 4 (4)
B
p=0.612
PFS (%)
Years since first infusion
No. of Patients
No. of Events
Rate at 5y % (95% CI)
< 18y 35 18 47 (30 - 63)
≥ 18y 6 2 67 (19 - 90)
< 18y 35 (0) 22 (0) 17 (2) 16 (2) 14 (3) 13 (4) 13 (4) 11 (6) 9 (8)
≥ 18y 6 (0) 3 (1) 3 (1) 2 (2) 2 (2) 1 (3) 1 (3) 1 (3) 0 (4)
C
p=0.061
CIR (%)
Years since first infusion
No. of Patients
No. of Events
Rate at 5y % (95% CI)
myeloid 20 4 20 (6 - 39)
lymphoblastic 21 10 51 (28 - 71)
myeloid 20 (0) 14 (0) 12 (1) 11 (2) 10 (3) 8 (5) 8 (5) 7 (6) 5 (8)
lymphoblastic 21 (0) 11 (1) 8 (2) 7 (2) 6 (2) 6 (2) 6 (2) 5 (3) 4 (4)
D
p=0.491
CIR (%)
Years since first infusion
No. of Patients
No. of Events
Rate at 5y % (95% CI)
< 18y 35 13 38 (22 - 54)
≥ 18y 6 1 17 (1 - 52)
< 18y 35 (0) 22 (0) 17 (2) 16 (2) 14 (3) 13 (4) 13 (4) 11 (6) 9 (8)
≥ 18y 6 (0) 3 (1) 3 (1) 2 (2) 2 (2) 1 (3) 1 (3) 1 (3) 0 (4)
E
p=0.977
NRM (%)
Years since first infusion
No. of Patients
No. of Events
Rate at 5y % (95% CI)
myeloid 20 3 15 (4 - 33)
lymphoblastic 21 3 14 (4 - 32)
myeloid 20 (0) 14 (0) 12 (1) 11 (2) 10 (3) 8 (5) 8 (5) 7 (6) 5 (8)
lymphoblastic 21 (0) 11 (1) 8 (2) 7 (2) 6 (2) 6 (2) 6 (2) 5 (3) 4 (4)
F
p=0.887
NRM (%)
Years since first infusion
No. of Patients
No. of Events
Rate at 5y % (95% CI)
< 18y 35 5 14 (5 - 28)
≥ 18y 6 1 17 (1 - 52)
< 18y 35 (0) 22 (0) 17 (2) 16 (2) 14 (3) 13 (4) 13 (4) 11 (6) 9 (8)
≥ 18y 6 (0) 3 (1) 3 (1) 2 (2) 2 (2) 1 (3) 1 (3) 1 (3) 0 (4)

Figure S9

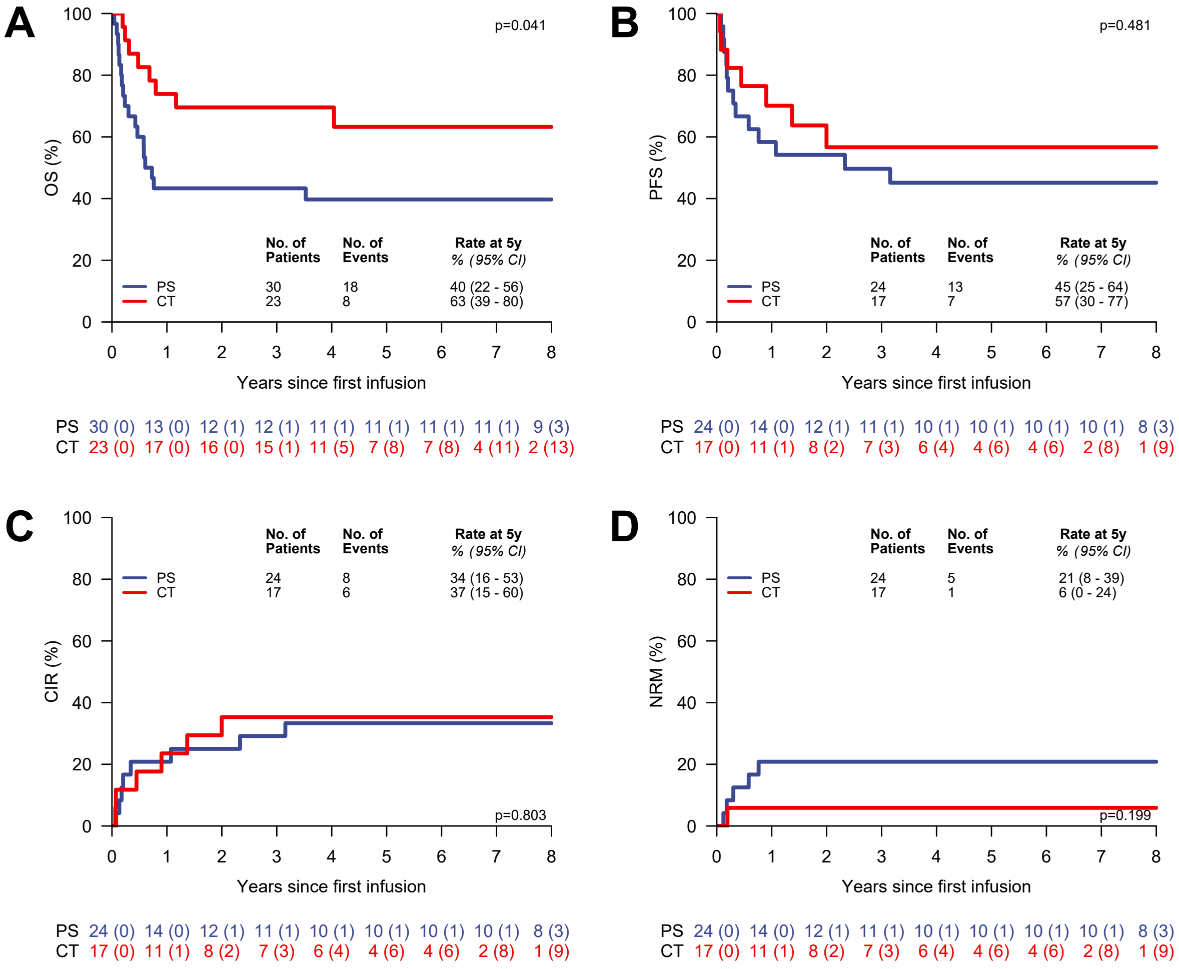

A
p=0.041
OS (%)
No. of Patients
No. of Events
Rate at 5y % (95% CI)
PS 30 18 40 (22 - 56)
CT 23 8 63 (39 - 80)
Years since first infusion
PS 30 (0) 13 (0) 12 (1) 12 (1) 11 (1) 11 (1) 11 (1) 11 (1) 9 (3)
CT 23 (0) 17 (0) 16 (0) 15 (1) 11 (5) 7 (8) 7 (8) 4 (11) 2 (13)
B
p=0.481
PFS (%)
No. of Patients
No. of Events
Rate at 5y % (95% CI)
PS 24 13 45 (25 - 64)
CT 17 7 57 (30 - 77)
Years since first infusion
PS 24 (0) 14 (0) 12 (1) 11 (1) 10 (1) 10 (1) 10 (1) 10 (1) 8 (3)
CT 17 (0) 11 (1) 8 (2) 7 (3) 6 (4) 4 (6) 4 (6) 2 (8) 1 (9)
C
CIR (%)
No. of Patients
No. of Events
Rate at 5y % (95% CI)
PS 24 8 34 (16 - 53)
CT 17 6 37 (15 - 60)
p=0.803
Years since first infusion
PS 24 (0) 14 (0) 12 (1) 11 (1) 10 (1) 10 (1) 10 (1) 10 (1) 8 (3)
CT 17 (0) 11 (1) 8 (2) 7 (3) 6 (4) 4 (6) 4 (6) 2 (8) 1 (9)
D
NRM (%)
No. of Patients
No. of Events
Rate at 5y % (95% CI)
PS 24 5 21 (8 - 39)
CT 17 1 6 (0 - 24)
p=0.199
Years since first infusion
PS 24 (0) 14 (0) 12 (1) 11 (1) 10 (1) 10 (1) 10 (1) 10 (1) 8 (3)
CT 17 (0) 11 (1) 8 (2) 7 (3) 6 (4) 4 (6) 4 (6) 2 (8) 1 (9)

Figure S10

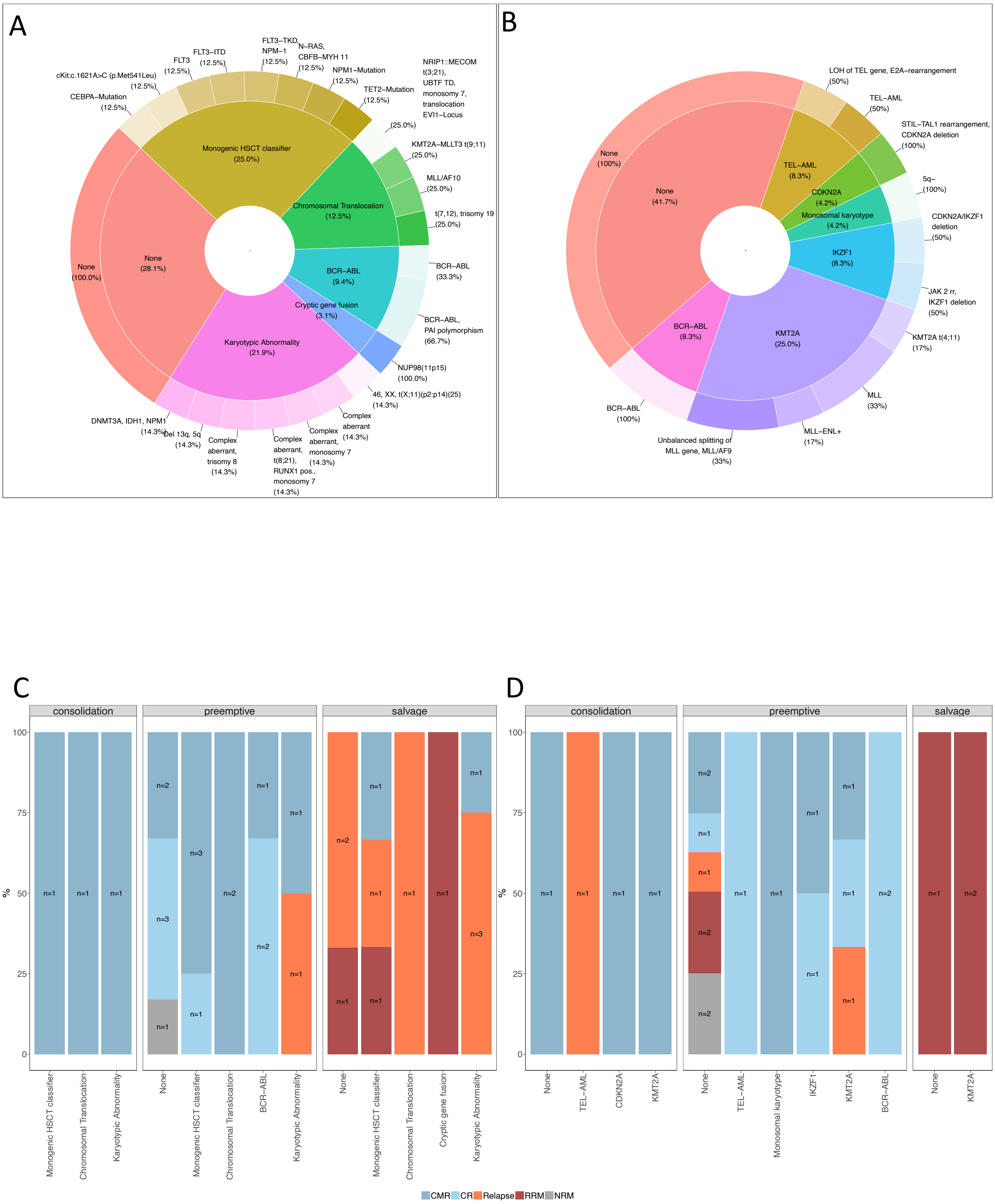
A
B
C
D
Monogenic HSCT classifier (25.0%)
Chromosomal Translocation (12.5%)
BCR–ABL (9.4%)
Cryptic gene fusion (3.1%)
Karyotypic Abnormality (21.9%)
None (28.1%)
None (100.0%)
None (41.7%)
TEL–AML (8.3%)
CDKN2A (4.2%)
Monosomal karyotype (4.2%)
IKZF1 (8.3%)
KMT2A (25.0%)
BCR–ABL (8.3%)
None (100%)
consolidation
preemptive
salvage
CMR CR Relapse RRM NRM

Supplementary Table 1

T-Cell Doses (x10^6/kg) and Number of Infusions

Characteristic	Total (n=41; %)	MD Relapse prevention (n=22; %)	MMD Relapse prevention (n=19; %)	P value	Salvage (n=15; %)
**Number of infusions**				.143	
Median	2.0	2.5	2.0		2.0
1st Qu, 3rd Qu	1.0, 4.0	2.0, 4.0	1.0, 3.0		2.0, 4.0
Min-Max	1 - 10	1 - 9	1 - 10		1 - 9
1	16 (29)	4 (18)	9 (47)		3 (20)
2	16 (29)	7 (32)	4 (21)		5 (33)
3	7 (13)	3 (14)	2 (11)		2 (13)
4	7 (13)	5 (23)	0 (0)		2 (13)
5	2 (4)	1 (5)	1 (5)		0 (0)
6	2 (4)	0 (0)	2 (11)		0 (0)
7	2 (4)	1 (5)	0 (0)		1 (7)
8	1 (2)	0 (0)	0 (0)		1 (7)
9	2 (4)	1 (5)	0 (0)		1 (7)
10	1 (2)	0 (0)	1 (5)		0 (0)
**Cumulative T-cell dose**				.059	
Median, (T-cells x10^6//kg)	6.5	16.9	5.0		10.0
1st Qu, 3rd Qu	4.9, 30.7	6.0, 27.1	1.0, 20.9		3.7, 44.9
Min-Max	1.0 - 721.4	1.0 - 721.4	1.0 - 381.7		1.0 – 351.0
Mean ± SD	50.9 ± 125.5	61.3 ± 157.8	30.9 ± 86.4		61.0 ± 119.0

IL15-CIK therapy: CIK infusions contained a heterogeneous lymphocyte population composed mainly of $CD3^+CD56^-$ T-cells, $CD3^+CD56^+$ T-NK cells, and $CD3^-CD56^+$ natural killer (NK) cells. The cell dose was calculated based on the potentially alloreactive T-cell subpopulation within this bulk population. Incremental dosing was performed at 4–6-week intervals. Cases of the consolidation and preemptive cohort are shown; MD, HLA-matched donor; HLA-MMD, mismatched donor.
Statistical testing was performed for differences between ≥9/10-HLA-matched donors and ≤8/10-HLA-mismatched donors.

Supplementary Table 2

T-Cell Dose ($x10^6$/kg) per Infusion

Dose	Total (N=56)	MD Relapse prevention (n=22)	MMD Relapse prevention (n=19)	P value	Salvage (n=15)
**1st T-cell dose, No.**	**56**	**22**	**19**	.536	**15**
Median, (T-cells $x10^6$/kg.)	1.0	1.0	1.0		1.0
1st Qu, 3rd Qu	1.0, 1.0	1.0, 3.6	1.0, 1.0		1.0, 1.0
Min-Max	0.7 - 100.0	0.7 - 100.0	1.0 - 5.0		0.1 - 9.4
Mean ± SD	5.3 ± 18.5	10.9 ± 28.9	1.6 ± 1.5		1.7 ± 2.4
**2nd T-cell dose, No.**	**40**	**18**	**10**	.060	**12**
Median, (T-cells $x10^6$/kg.)	5.0	5.1	5.0		5.0
1st Qu, 3rd Qu	5.0, 5.6	5.0, 9.1	5.0, 5.0		4.9, 5.0
Min-Max	1.0 - 100.0	4.9 - 100.0	3.7 - 10.0		1.0 - 53.4
Mean ± SD	12.0 ± 20.3	15.5 ± 26.1	5.5 ± 1.7		12.1 ± 18.6
**3rd T-cell dose, No.**	**24**	**11**	**6**	**.039**	**7**
Median, (T-cells $x10^6$/kg.)	10.0	10.2	5.0		10.0
1st Qu, 3rd Qu	5.0, 16.2	9.9, 21.1	4.3, 8.8		7.4, 52.7
Min-Max	2.0 - 200.0	5.0 - 100.0	2.0 - 20.0		5.0 - 200.0
Mean ± SD	28.7 ± 47.5	27.9 ± 36.1	7.7 ± 6.6		47.9 ± 74.6
**4th T-cell dose, No.**	**17**	**8**	**4**	.130	**5**
Median, (T-cells $x10^6$/kg.)	10.0	10.0	9.9		14.8
1st Qu, 3rd Qu	10.0, 10.9	10.0, 10.1	8.7, 10.0		10.9, 94.9
Min-Max	5.3 - 100.0	9.8 - 100.0	5.3 - 10.0		10.0- 100.0
Mean ± SD	25.6 ± 34.7	21.2 ± 31.8	8.8 ± 2.3		46.1 ± 46.9
**5th T-cell dose, No.**	**10**	**3**	**4**		**3**
Median, (T-cells $x10^6$/kg.)	9.9	10.0	9.8		10.0
1st Qu, 3rd Qu	8.2, 10.0	8.8, 25.0	9.0, 9.9		8.2, 32.8
Min-Max	6.5 - 55.6	7.7 - 40.0	6.5 - 10.0		6.5 - 55.6
Mean ± SD	16.6 ± 16.9	19.2 ± 18.0	9.0 ± 1.7		24.0 ± 27.4
**6th T-cell dose, No.**	**8**	**2**	**3**		**3**
Median, (T-cells $x10^6$/kg.)	9.2	32.5	8.3		10.0
1st Qu, 3rd Qu	7.5, 15.5	20.1, 45.0	7.7, 20.1		8.1, 10.0
Min-Max	6.1 - 57.4	7.7 - 57.4	7.1 - 32.0		6.1 - 10.0
Mean ± SD	17.3 ± 18.3	32.5 ± 35.2	15.8 ± 14.0		8.7 ± 2.3
**7th T-cell dose, No.**	**6**	**2**	**1**		**3**
Median, (T-cells $x10^6$/kg.)	20.0	37.1	46.4		10.0
1st Qu, 3rd Qu	8.0, 42.3	21.1, 53.2			8.6, 20.0
Min-Max	5.0 - 69.3	5.0 - 69.3			7.3 - 30.0
Mean ± SD	28.0 ± 25.8	37.1 ± 45.4			15.8 ± 12.4
**8th T-cell dos, No.**	**4**	**1**	**1**		**2**
Median, (T-cells $x10^6$/kg.)	42.9	74.3	100.0		9.8
1st Qu, 3rd Qu	10.6, 80.7				8.9, 10.6
Min-Max	8.1 - 100.0				8.1 - 11.4
Mean ± SD	48.5 ± 45.9				9.8 ± 2.3
**9th T-cell dose, No.**	**3**	**1**	**1**		**1**
Median, (T-cells $x10^6$/kg.)	80.4	80.4	98.0		12.0
1st Qu, 3rd Qu	46.2, 89.2				
Min-Max	12.0 - 98.0				
Mean ± SD	63.5 ± 45.4				
**10th T-cell dose, No.**	**1**	**0**	**1**		**0**
Median, (T-cells $x10^6$/kg.)	93.0		93.0		
1st Qu, 3rd Qu					
Min-Max					
Mean ± SD					

Post-HCT IL15-CIK infusions: IL15-CIK infusions contained a heterogeneous lymphocyte population composed mainly of $CD3^+CD56^-$ T-cells, $CD3^+CD56^+$ T-NK cells, and $CD3^-CD56^+$ natural killer (NK) cells. The cell dose was calculated based on the potentially alloreactive T-cell subpopulation within this bulk population. Incremental dosing was performed at 4–6-week intervals. Cases of the consolidation and preemptive cohort are shown for comparison between≥9/10-HLA-matched donors and ≤8/10-HLA-mismatched donors.

Supplementary Table 3A

Reported Adverse Events After Post-Transplant IL–15 CIK Therapy

Site / Adverse Event	Description	Mild	Moderate	Severe	Life-threatening	Fatal	Resolved
**CNS**							
	no						
**Head and Neck**							
	Conjunctivitis	2					2/2
	Otitis media	1					1/1
	Upper airway infection	1					1/1
**Lung**							
	Cough	2					2/2
	Pneumonia	1					1/1
	Resp. infection	1					1/1
	Progressive lung infiltration					1	0/1
**Cardio-vascular**							
	Catheter infection		1				1/1
**GIT, Liver**							
	Nausea/Vomiting	4					4/4
	Abdominal Pain	1					1/1
	Gastritis	1					1/1
	Gastroenteritis	1					1/1
	Diarrhea	3					3/3
	Obstipation	2					2/2
	Gastroenteritis	1					1/1
	Periproctic abscess		1				1/1
**Kidney, Bladder**							
	Cystitis	2					2/2
**Extremities**							
	Pain (legs)	5					5/5
	Bursitis	1					1/1
	Osteomyelitis		1				1/1
**Infection/ Reactivation**							
	CMV Reactivation		5	1			6/6
	AdV Reactivation		3	2			5/5
	EBV Reactivation	2					2/2
	RSV	1				1	1/2
	hMPV	1					1/1
	Influenzas	4					4/4
	Parainfluenza	1					1/1
	JC-Virus	2	1				3/3
	Aspergillus		1			1	1/2
	VZV	1					1/1
	Bacteremia		1				1/1
**Inflammation***							
	Flu-like symptoms	6					6/6
	CRS	1					1/1
	Fever	4					4/4
	Allergic reaction	1					1/1
**Skin***							
	Dermatitis	2					2/2
	Atopic eczema		1				1/1
	Exanthem	1					1/1
	Erythema (facial)	1					1/1

Summary of reported adverse events (AEs) and serious adverse events (SAEs) observed after IL15-CIK infusion in HCT cases is shown. Severity was classified according to CTCAE v5.0, and events potentially related to IL15-CIK therapy are indicated by* and shaded in gray. AE, adverse event; SAE, serious adverse event; CNS, central nervous system; GIT, gastrointestinal tract; CMV, cytomegalovirus; AdV, adenovirus; EBV, Epstein Barr virus; RSV; respiratory syncytial virus; hMPV; human metapneumovirus; JC, John Cunningham virus, VZV, varicella zoster virus; CRS, cytokine release syndrome.

Supplementary Table 3B

Acute and Chronic GVHD: Incidence and Severity

Overall Grade	Total (N=56; %)	MD (n=22; %)	MMD (n=19; %)	Salvage (n=15; %)
**aGVHD**				
**No**	39 (70)	15 (68)	10 (53)	14 (93)
**1**	7 (13)	4 (18)	2 (11)	1 (7)
**2**	8 (14)	2 (9)	6 (32)	0 (0)
**3**	2 (4)	1 (5)	1 (5)	0 (0)
**4**	0 (0)	0 (0)	0 (0)	0 (0)
**cGVHD**				
**No**	52 (93)	20 (91)	17 (89)	15 (100)
**Limited**	4 (7)	2 (9)	2 (11)	0 (0)
**Extensive**	0 (0)	0 (0)	0 (0)	0 (0)

Post-HCT IL15-CIK infusions contained a potentially alloreactive T-cell subpopulation within the bulk population. GVHD was assessed weekly during treatment. Cases from the consolidation and preemptive cohorts are presented together to illustrate differences between ≥9/10 HLA-matched donors and ≤8/10 HLA-mismatched donors in the context of preventive treatment intent. The overview summarizes acute and chronic GVHD occurrence following IL15-CIK infusion, including grade distribution, and dose-limiting toxicities (DLTs; ≥ grade 3 aGVHD and extensive cGVHD). Timing and more details are shown in Supplementary Table S4. GVHD, graft-versus-host disease; a, acute; c, chronic; MD, HLA-matched donor; MMD, HLA-mismatched donor.

Supplementary Table 4, Patient Cohort with Non-Relapse Mortality (NRM) & acute GVHD (aGVHD) ≥ Grade 2

Age / Gender P-Index	Disease Status At HCT	Conditioning regimen	Donor	Graft Manipulation $CD34^+$/kg	Sero-therapy	Trigger for 1st CIK	Age at 1st IL15-CIK P-Index	Maximum single dose T-cells/kg ($x10^6$)	Total Number of IL15-CIK	Cumulative T-cells/kg ($x10^6$)	GVHD (Overall) Grade	Onset 1st IL15-CIK to GVHD (days)	Onset last IL15-CIK to GVHD (days)	AE	NRM	Best Response to IL15-CIK	Subsequent Treatment	LFU after last IL15-CIK
**Non-Relapse Mortality**																		
11, f<br>PI: 100%	AML,<br>1st HCT,<br>CR2	Clo, Cy, Eto, Flu, Thio, Mel	MMFD	PBSC<br>3/19depl.<br>8.6 Mio.	Campath	BM: MC,<br>CD33/34<br>Subpop. 1-5%	12<br>PI: 90%	1	2	4.7	Skin, 2	28	28	CMV reactivation<br>Prior to CIK<br>CRS grade 1	Multiorgan failure,<br>ECMO	CMR		Died,<br>21 days
7, m<br>PI: 70%	AML M2,<br>complex karyotype<br>2nd relapse,<br>4th HCT, NR (Aplasia)	Flu	MMFD	PBSC<br>3/19depl.<br>3.8 Mio.	Campath	Consolidation	7<br>PI: 70%	5	3	8	no			Local infection<br>(Staph hemolyt.<br>Gastro tube)<br>cardiac, renal, hepatic dysfunction	Fungal Infection<br>Orbital		No	Died,<br>6.4 months
10, m<br>PI: 70%	Pre-T-ALL, 5q-,<br>2nd HCT, CR2	Clo, Cy, Eto, Flu, Thio, Mel	MMFD	PBSC<br>3/19depl.<br>12.5 Mio.	Campath	MRD<br>PI: 100%	10<br>PI: 100%	1	1	1	Skin, GIT, 2	28	28	CMV, AdV<br>EBV, JC<br>VZV, Asp.	TMA	CMR	No	Died,<br>3.6 months
17, m<br>PI: 40%	cALL,<br>2nd Relapse<br>2nd HCT, NR	Clo, Thio, Mel	MMFD	PBSC<br>3/19depl.<br>7.0 Mio.	Campath	MRD,<br>2,2 x 10^{-3}	17<br>PI: 70%	6.1	2	11.1	Skin, 1	35	15	JC Virus	Cardiac Failure	CMR	No	Died,<br>1.5 months
9, m<br>PI: 90%	T-ALL,<br>very early relapse,<br>2nd HCT, NR	Clo, Cy, Eto, Flu, Thio, Mel	MMFD	PBSC<br>3/19depl.<br>12.4 Mio.	Campath	MC 10-20%,<br>MRD 1.0 x 10^{-6}	9<br>PI: 70%	5	1	5	Skin, GIT, 2	21	21	AdV, BK-Virus<br>Prior to CIK	TMA	CMR	No	Died,<br>1.4 months
68, f<br>n.n.	Secondary AML,<br>1st HCT, CR1	n.n.	MUD	PBSC<br>No, 7.4 Mio.	ATG	PB: MC,<br>CD34 Subpop.<br>1-5%	68	10	3	16	no			RSV	RSV	CMR	No	Died,<br>4.9 months
**acute GVHD ≥ Grade 2**																		
7, m	cALL, JAK 2 rr, del IKZF1 Gen<br>3rd relapse,<br>2nd HCT, CR4	n.n.	MMFD	PBSC<br>TCRa/bdepl.<br>40 Mio.	Campath	Cytogenetic relapse.	7	1.13	1	1.13	Skin, GIT, 2	18	18	-	-	CMR	No	Alive,<br>6.8 years
12, m	AML, M5<br>1st HCT, CR1	n.n.	MSD	BM<br>No<br>3.6 Mio.	No	BM: MC, CD34 Subpop. 1-5%	12	5.5	3	17.75	Skin, 2	47	18	-	-	CMR	2nd CIK cycle	Alive,<br>2.7 years
69, m	CMML,<br>TET2-Mutation<br>1st HCT, CR1	n.n.	MUD	PBSC<br>No<br>7.4 Mio.	ATG	BM: MC, CD34 Subpop. 1-5%	69	10	5	34.3	Skin, liver, 3	158	18	-	-	CMR	No	Alive,<br>7.0 years
71, m	Sek AML<br>NPM1 mutation<br>1st HCT, CR1	n.n.	MUD	BM<br>No<br>1.7 Mio.	Campath	PB: MC, 35%	71	1	1	1	GIT, 2	1	1	Influenza	No	CMR	No	Alive.<br>2.9 years
9, m<br>PI: 100%	cALL,<br>3rd relapse,<br>2nd HCT NR	Clo, Daunoxome, Ara-C, Flu, Thio, Mel	MMFD	PBSC<br>3/19depl.<br>18.6 Mio.	Campath	Consolidation	9<br>PI: 100%	20	3	31	Skin, GIT, Liver, 3, limited cGVHD	71	19		No		No	Alive,<br>10 years
1, m<br>PI: 100%	Pro-B-ALL, MLL,<br>2nd HCT, CR2	Clo, Cy, Eto, Flu, Thio, Mel	MMFD	PBSC<br>3/19depl.<br>31.1 Mio.	Campath	Consolidation	2<br>PI: 100%	5	1	5	Skin, GIT, 2	27	27	Catheter infection<br>(bacteremia)	No		No	Alive,<br>10 years
10, m<br>PI: 100%	AML M5, Ph^+,<br>2nd HCT, NR	Clo, Cy, Eto, Flu, Thio, Mel	MMFD	PBSC<br>3/19depl.<br>19.1 Mio.	Campath	BM: MC 1%,	10<br>PI: 100%	4.96	2	5.96	Skin, GIT, 2	52	5		No	CMR	No	Alive,<br>9.6 years

*One IL15-CIK infusion each was administered before the occurrence and after the resolution of grade 2 aGVHD.

P-Index, performance status; HCT, hematopoietic stem cell transplantation; CMML, chronic myelomonocytic leukemia; AML, acute myeloid leukemia; ALL, acute lymphoblastic leukemia; CR, complete remission; CMR, complete molecular remission; NR, not in remission; Clo, Clofarabine; Daunoxome, Daunorubicin liposomal; Ara-C, Cytarabine; Flu; Fludarabine; Thio, Thiotepa; Mel, Melphalan; Cy, Cyclophosphamide; Eto, Etoposide; MMFD, haploidentical family donor; MUD, matched unrelated donor; MSD, matched sibling donor; PBSC, peripheral blood stem cells; BM, bone marrow; ATG, anti-thymocyte globulin; MC, mixed chimerism, MRD, minimal/measurable disease; IL15-CIK, IL15-activated cytokine-induced killer cells; a/cGVHD, acute/chronic graft-versus-host disease; GIT, gastrointestinal tract; AE, adverse event; NRM, non-relapse mortality, treatment-related toxicity; ECMO, extracorporeal membrane oxygenation; TMA, thrombotic microangiopathy; RSV, respiratory synthetical virus. White and reddish background: clinical trial and pilot study population.

Supplementary Table 5

Relapse-Preventive Case Characteristics: Pilot Study (PS) vs. Clinical Trial (CT)

Characteristic	Total (n=41; %)	PS (n=24; %)	CT (n=17; %)	P value
**Gender, No. (%)**				1.000
Female	14 (34)	8 (33)	6 (35)	
Male	27 (66)	16 (67)	11 (65)	
**Age at 1st IL15-CIK, years**				.874
Median (range)	10.3 (1.3 – 71.9)	10.4 (1.3 – 69.0)	8.3 (3.6 – 71.9)	
**Age group at 1st IL15-CIK, No. (%)**				.066
< 18 years	35 (85)	23 (96)	12 (71)	
Median (range)	9.6 (1.3 – 17.6)	10.3 (1.3 – 17.6)	7.4 (3.6 – 12.7)	
≥ 18 years	6 (15)	1 (4)	5 (29)	
Median (range)	69 (20.0 -71.9)	69.0	69.9 (20.0 – 71.9)	
**Disease**				**.028**
**Myeloid, No. (%)**	20 (49)	8 (33)	12 (71)	
AML	17 (41)	7 (29)	10 (59)	
CML	1 (2)	1 (4)	0 (0)	
CMML	1 (2)	0 (0)	1 (6)	
MRC	1 (2)	0 (0)	1 (6)	
**Lymphoblastic, No. (%)**	21 (51)	16 (67)	5 (29)	
(pre/pro-) B-ALL	14 (34)	9 (38)	5 (29)	
(pre-) T-ALL	7 (17)	7 (29)	0 (0)	
**Remission status at HCT, No. (%)**				.093
1. CR	15 (37)	7 (29)	8 (47)	
2. CR	12 (29)	7 (29)	5 (29)	
≥ 3. CR	4 (10)	1 (4)	3 (18)	
NR	10 (24)	9 (38)	1 (6)	
**No. of HCT, No. (%)**				**.033**
1st	26 (63)	12 (50)	14 (82)	
2nd	14 (34)	11 (46)	3 (18)	
≥ 3rd	1 (2)	1 (4)	0 (0)	
**Donor, No. (%)**				**.025**
**Matched donor (MD)**	22 (54)	9 (38)	13 (76)	
MSD	2 (5)	1 (4)	1 (6)	
MUD	20 (49)	8 (33)	12 (71)	
**Mismatched donor (MMD)**	19 (46)	15 (63)	4 (24)	
MMFD	18 (44)	15 (63)	3 (18)	
MMUD	1 (2)	0 (0)	1 (6)	
**Stem cell source, No. (%)**				**.048**
BM	14 (34)	5 (21)	9 (53)	
PBSC	27 (66)	19 (79)	8 (47)	
***In vitro* T-cell depletion, No. (%)**				**.011**
No	21 (51)	8 (33)	13 (76)	
Yes	20 (49)	16 (67)	4 (24)	
**Graft composition**				
**CD34$^+$**x10^6/kg				.098
Median	7.4	11.6	7.1	
(range)	(1.7 - 40.0)	(2.3 - 31.1)	(1.7 - 40.0)	
**CD3$^+$**x10^6/kg				.061
Median	0.3	0.2	29.4	
(range)	(0.0 - 500.4)	(0.0 - 500.4)	(0.0 - 107.3)	
**Serotherapy, No. (%)**				.432
No	2 (5)	1 (4)	1 (6)	
ATG	21 (51)	10 (42)	11 (65)	
Campath	17 (41)	12 (50)	5 (29)	
OKT3	1 (2)	1 (4)	0 (0)	
**ATG dose (mg/kg)**				.127
Median	60.0	60.0	60.9	
(range)	(27.7 - 83.6)	(40.0 - 60.0)	(27.7 - 83.6)	
**Campath dose (mg/kg)**				.141
Median	0.5	0.5	1.3	
(range)	(0.3 - 17.6)	(0.3 - 17.6)	(0.4 - 2.0)	
**IL15-CIK: Treatment intent cohort, No. (%)**				**.016**
Consolidation	7 (17)	7 (29)	0 (0)	
Preemptive	34 (83)	17 (71)	17 (100)	
**Time from HCT to 1st IL15-CIK**				**.010**

**months**				
Median (range)	4.1 (0.7 – 31.9)	2.1 (0.7 – 31.9)	6.2 (1.4 – 17.8)	
**Time from HCT to 1st IL15-CIK, No. (%)**				.054
< 100 days	18 (44)	14 (58)	4 (24)	
≥ 100 days	23 (56)	10 (42)	13 (76)	
**Previous treatment course, No. (%)**				.254
No	38 (93%)	21 (88%)	17 (100%)	
Yes	3 (7%)	3 (12%)	0 (0%)	

Post-HCT IL15-CIK therapy (shaded), IL15-activated cytokine-induced killer cells; HCT, hematopoietic stem cell transplantation; AML, acute myeloid leukemia; CML, chronic myeloid leukemia; CMML, chronic myelomonocytic leukemia; MRC, myelodysplasia-related changes; cALL, common acute lymphoblastic leukemia; T-ALL, T-cell ALL, CR, complete remission; NR, not in remission; MD, HLA-matched donor; MMD, HLA-mismatched donor; MSD, matched sibling donor; MUD, HLA-matched unrelated donor; MMFD, HLA-mismatched family donor; MMUD, HLA-mismatched, unrelated donor; BM, bone marrow; PBSC, peripheral blood stem cells; ATG, anti-thymoglobulin; CT, clinical trial; PS, pilot study. Statistical testing was performed to assess differences between PS and CT cases with relapse-preventive intent.

Supplementary Table 6

Relapse-Preventive Case Characteristics in the Pediatric AML Subcohort

Characteristic	Total (n=13; %)	MD (n=6; %)	MMD (n=7; %)	P value
**Gender, No. (%)**				.266
Female	5 (38)	1 (17)	4 (57)	
Male	8 (62)	5 (83)	3 (43)	
**Age at 1st IL15-CIK, years**				.063
Median (range)	9.2 (3.6 – 17.2)	6.5 (3.6 – 12.7)	10.5 (6.9 – 17.2	
**Remission status at HCT, No. (%)**				**.004**
1. CR	5 (38)	5 (83)	0 (0)	
2. CR	6 (46)	1 (17)	5 (71)	
≥ 3. CR	0 (0)	0 (0)	0 (0)	
NR	2 (15)	0 (0)	2 (29)	
**No. of HCT, No. (%)**				**.035**
1st	9 (69)	6 (100)	3 (43)	
2nd	3 (23)	0 (0)	3 (43)	
≥ 3rd	1 (8)	0 (0)	1 (14)	
**Donor, No. (%)**				
MSD	1 (8)	1 (17)		
MUD	5 (38)	5 (83)		
MMFD	7 (54)		7 (100)	
MMUD	0 (0)		0 (0)	
**Stem cell source, No. (%)**				**<.001**
BM	6 (46)	6 (100)	0 (0)	
PBSC	7 (54)	0 (0)	7 (100)	
***In vitro* T-cell depletion, No. (%)**				**<.001**
No	6 (46)	6 (100)	0 (0)	
Yes	7 (54)	0 (0)	7 (100)	
**Graft composition**				
**$CD34^+$**$x10^6$/kg				.063
Median	7.1	6.7	12.1	
(range)	(3.6 - 21.1)	(3.6 - 7.4)	(3.9 - 21.1)	
**$CD3^+$**$x10^6$/kg				**.042**
Median	0.2	61.5	0.1	
(range)	(0.0 - 107.3)	(0.1 - 107.3)	(0.0 - 0.3)	
**Serotherapy, No. (%)**				**.008**
No	1 (8)	1 (17)	0 (0)	
ATG	6 (46)	5 (83)	1 (14)	
Campath	6 (46)	0 (0)	6 (86)	
OKT3	0 (0)	0 (0)	0 (0)	
**ATG dose (mg/kg)**				.137
Median	60.6	61.3	27.7	
(range)	(27.7 - 83.6)	(60.0 - 83.6)		
**Campath dose (mg/kg)**				
Median	0.5	NA	0.5	
(range)	(0.3 - 17.6)		(0.3 - 17.6)	
**IL15-CIK: Treatment intent cohort, No. (%)**				.079
Consolidation	3 (23)	0 (0)	3 (43)	
Preemptive	10 (77)	6 (100)	4 (57)	
**IL15-CIK: Study group, No. (%)**				.103
PS	6 (46)	1 (17)	5 (71)	
CT	7 (54)	5 (83)	2 (29)	
**Time from HCT to 1st IL15-CIK, months**				**.007**
Median (range)	3.2 (1.0 – 8.7)	5.2 (2.4 – 8.7)	1.9 (1.0 – 3.4)	
**Time from HCT to 1st IL15-CIK, No. (%)**				**.029**
< 100 days	7 (54)	1 (17)	6 (86)	
≥ 100 days	6 (46)	5 (83)	1 (14)	
**Previous treatment course, No. (%)**				1.000
No	12 (92%)	6 (100%)	6 (86%)	
Yes	1 (8%)	0 (0%)	1 (14%)	

Post-HCT IL15-CIK (shaded), IL15-activated cytokine-induced killer cells; HCT, hematopoietic stem cell transplantation; AML, acute myeloid leukemia; CML, chronic myeloid leukemia; CMML, chronic myelomonocytic leukemia; MRC, myelodysplasia-related changes; cALL, common acute lymphoblastic leukemia; T-ALL, T-cell ALL, CR, complete remission; NR, not in remission; rlps., relapse; MD, matched donor; MMD, mismatched donor; MSD, matched sibling donor; MUD, matched unrelated donor; MMFD, mismatched family donor; MMUD, mismatched, unrelated donor; BM, bone marrow; PBSC, peripheral stem cells; ATG, anti-thymoglobulin; CT, clinical

trial; PS, pilot study. Testing was performed for differences between matched donors and mismatched donors. Statistical testing was performed for differences between ≤8/10-HLA-matched donors and ≥9/10-HLA-mismatched donors.

Supplementary Table 7

T-Cell Doses (x10^6/kg) and Numbers of IL15-CIK Infusions: Pediatric AML

Characteristic	Total (n=13; %)	MD (n=6; %)	MMD (n=7; %)	P value
**Number of infusions**				.093
Median	2.0	3.5	2.0	
1st Qu, 3rd Qu	2.0, 4.0	2.2, 4.0	1.0, 2.5	
Min-Max	1.0 - 9.0	2.0 - 9.0	1.0 - 5.0	
1	3 (23)	0 (0)	3 (43)	
2	4 (31)	2 (33)	2 (29)	
3	2 (15)	1 (17)	1 (14)	
4	2 (15)	2 (33)	0 (0)	
5	1 (8)	0 (0)	1 (14)	
6	0 (0)	0 (0)	0 (0)	
7	0 (0)	0 (0)	0 (0)	
8	0 (0)	0 (0)	0 (0)	
9	1 (8)	1 (17)	0 (0)	
10	0 (0)	0 (0)	0 (0)	
**Cumulative T-cell dose**				**.044**
Median (T-cells x 10^6/kg)	6.1	21.6	4.7	
1st Qu, 3rd Qu	4.7, 25.5	9.0, 25.9	1.0, 7.0	
Min-Max	1.0 - 721.4	6.0 - 721.4	1.0 - 30.6	
Mean ± SD	65.8 ± 197.2	133.8 ± 288.0	7.5 ± 10.6	

IL15-CIK therapy: IL15-CIK infusions contained a heterogeneous lymphocyte population composed mainly of $CD3^+CD56^-$ T-cells, $CD3^+CD56^+$ T-NK cells, and $CD3^-CD56^+$ natural killer (NK) cells. The cell dose was calculated based on the potentially alloreactive T-cell subpopulation within this bulk population. Incremental dosing was performed at 4–6-week intervals. Cases of the consolidation and preemptive cohort are shown; MD, HLA-matched donor; HLA-MMD, mismatched donor.
Statistical testing was performed for differences between ≥9/10-HLA-matched donors and ≤8/10-HLA-mismatched donors.

Supplementary Table 8

T-Cell Dose ($x10^6$/kg) per Infusion: Pediatric AML

Dose	Total (n=13)	MD (n=6)	MMD (n=7)	P value
**1st T-cell dose, No.**	**13**	**6**	**7**	.112
Median, (T-cells $x10^6$/kg.)	1.0	1.0	1.0	
1st Qu, 3rd Qu	1.0, 1.0	1.0, 1.0	1.0, 1.0	
Min-Max	1.0 - 100.0	1.0 - 100.0	1.0 - 1.0	
Mean ± SD	8.6 ± 27.5	17.5 ± 40.4	1.0 ± 0.0	
**2nd T-cell dose, No.**	**10**	**6**	**4**	.066
Median, (T-cells $x10^6$/kg.)	5.0	5.0	4.9	
1st Qu, 3rd Qu	4.9, 5.1	5.0, 5.4	4.6, 5.0	
Min-Max	3.7 - 100.0	4.9 - 100.0	3.7 - 5.0	
Mean ± SD	14.4 ± 30.1	20.9 ± 38.7	4.6 ± 0.6	
**3rd T-cell dose, No.**	**6**	**4**	**2**	
Median, (T-cells $x10^6$/kg.)	9.9	10.6	3.5	
1st Qu, 3rd Qu	6.2, 10.9	10.0, 33.4	2.8, 4.2	
Min-Max	2.0 - 100.0	9.8 - 100.0	2.0 - 5.0	
Mean ± SD	23.0 ± 37.9	32.8 ± 44.8	3.5 ± 2.1	
**4th T-cell dose, No.**	**4**	**3**	**1**	
Median, (T-cells $x10^6$/kg.)	9.9	10.0	9.9	
1st Qu, 3rd Qu	9.9, 32.5	9.9, 55.0		
Min-Max	9.8 - 100.0	9.8 - 100.0		
Mean ± SD	32.4 ± 45.1	39.9 ± 52.0		
**5th T-cell dose, No.**	**2**	**1**	**1**	
Median, (T-cells $x10^6$/kg.)	24.9	40.0	9.9	
1st Qu, 3rd Qu	17.4, 32.5			
Min-Max	9.9 - 40.0			
Mean ± SD	24.9 ± 21.3			
**6th T-cell dose, No.**	**1**	**1**	**0**	
Median, (T-cells $x10^6$/kg.)	57.4	57.4		
1st Qu, 3rd Qu				
Min-Max				
Mean ± SD				
**7th T-cell dose, No.**	**1**	**1**	**0**	
Median, (T cells $x10^6$/kg.)	69.3	69.3		
1st Qu, 3rd Qu				
Min-Max				
Mean ± SD				
**8th T-cell dos, No.**	**1**	**1**	**0**	
Median, (T-cells $x10^6$/kg.)	74.3	74.3		
1st Qu, 3rd Qu				
Min-Max				
Mean ± SD				
**9th T-cell dose, No.**	**1**	**1**	**0**	
Median, (T-cells $x10^6$/kg.)	80.4	80.4		
1st Qu, 3rd Qu				
Min-Max				
Mean ± SD				
**10th T-cell dose, No.**	**0**	**0**	**0**	
Median, (T-cells $x10^6$/kg.)				
1st Qu, 3rd Qu				
Min-Max				
Mean ± SD				

Post-HCT IL15-CIK: IL15-CIK infusions contained a heterogeneous lymphocyte population composed mainly of $CD3^+CD56^-$ T-cells, $CD3^+CD56^+$ T-NK cells, and $CD3^-CD56^+$ natural killer (NK) cells. The cell dose was calculated based on the potentially alloreactive T-cell subpopulation within this bulk population. Incremental dosing was performed at 4–6-week intervals. Cases of the consolidation and preemptive cohort are shown. IL15-activated cytokine induced killer cell infusions, MD, HLA-matched donor; MMD, HLA-mismatched donor.

Supplementary Table 9
Case Characteristics: Overall Survival

Characteristic	Total (n=53; %)	MD (n=25; %)	MMD (n=28; %)	P value
**Gender, No. (%)**				1.000
Female	21 (40)	10 (40)	11 (39)	
Male	32 (60)	15 (60)	17 (61)	
**Age at 1st IL15-CIK, years**				.057
Median (range)	10.5 (0.6 - 71.9)	13.6 (3.2 - 71.9)	9.6 (0.6 - 58.7)	
**Age group at 1st IL15-CIK, No. (%)**				**.007**
< 18 years	41 (77)	15 (60)	26 (93)	
Median (range)	8.8 (0.6 – 17.6)	8.3 (3.2 – 15.7)	9.6 (0.6 – 17.6)	
≥ 18 years	12 (23)	10 (40)	2 (7)	
Median (range)	58.6 (20.0 – 71.9)	63.7 (20.0 – 71.9)	41.1 (23.5 – 58.7)	
**Disease**				.265
**Myeloid, No. (%)**	31 (58)	17 (68)	14 (50)	
AML	28 (53)	15 (60)	13 (46)	
CML	1 (2)	0 (0)	1 (4)	
CMML	1 (2)	1 (4)	0 (0)	
MRC	1 (2)	1 (4)	0 (0)	
**Lymphoblastic, No. (%)**	22 (42)	8 (32)	14 (50)	
(pre/pro-) B-ALL	14 (26)	6 (24)	8 (29)	
(pre-) T-ALL	8 (15)	2 (8)	6 (21)	
**Remission status at HCT, No. (%)**				**.005**
1. CR	14 (26)	12 (48)	2 (7)	
2. CR	18 (34)	7 (28)	11 (39)	
≥ 3. CR	4 (8)	2 (8)	2 (7)	
NR	17 (32)	4 (16)	13 (46)	
**No. of HCT, No. (%)**				**<.001**
1st	32 (60)	23 (92)	9 (32)	
2nd	18 (34)	1 (4)	17 (61)	
≥ 3rd	3 (6)	1 (4)	2 (7)	
**Donor, No. (%)**				
MSD	2 (4)	2 (8)		
MUD	23 (43)	23 (92)		
MMFD	27 (51)		27 (96)	
MMUD	1 (2)		1 (4)	
**Stem cell source, No. (%)**				**<.001**
BM	15 (28)	13 (52)	2 (7)	
PBSC	38 (72)	12 (48)	26 (93)	
***In vitro* T-cell depletion, No. (%)**				**<.001**
No	26 (49)	24 (96)	2 (7)	
Yes	27 (51)	1 (4)	26 (93)	
**Graft composition**				
**CD34$^+$**x10^6/kg				**<.001**
Median	8.3	7.1	12.4	
(range)	(1.7 - 40.0)	(1.7 - 14.7)	(2.5 - 40.0)	
**CD3$^+$**x10^6/kg				**<.001**
Median	1.0	46.8	0.2	
(range)	(0.0 - 500.4)	(10.0 - 500.4)	(0.0 - 13.3)	
**Serotherapy, No. (%)**				**<.001**
No	4 (8)	4 (16)	0 (0)	
ATG	27 (51)	18 (72)	9 (32)	
Campath	21 (40)	3 (12)	18 (64)	
OKT 3	1 (2)	0 (0)	1 (4)	
**ATG dose (mg/kg)**				.478
Median	60.0	60.0	40.0	
(range)	(22.2 - 120.0)	(22.2 - 83.6)	(27.7 - 120.0)	
**Campath dose (mg/kg)**				**.011**
Median	0.5	1.5	0.5	
(range)	(0.3 - 17.6)	(1.2 - 2.0)	(0.3 - 17.6)	
**IL15-CIK: Treatment intent cohort, No. (%)**				.442
Consolidation	7 (13)	0 (0)	7 (25)	
Preemptive	31 (58)	19 (76)	12 (43)	
Salvage	15 (28)	6 (24)	9 (32)	
**IL15-CIK: Study group, No. (%)**				**<.001**
PS	30 (57)	8 (32)	22 (79)	
CT	23 (43)	17 (68)	6 (21)	
**Time from HCT to 1st IL15-CIK, months**				**<.001**
Median (range)	3.8 (0.7 - 43.9)	6.3 (2.4 – 43.9)	1.7 (0.7 – 31.9)	
**Time from HCT to 1st IL15-CIK, No. (%)**				**<.001**
< 100 days	24 (45)	2 (8)	22 (79)	
≥ 100 days	29 (55)	23 (92)	6 (21)	

Characteristic	Total (n=53; %)	MD (n=25; %)	MMD (n=28; %)	P value
**Previous treatment course, No. (%)**				.238
No	50 (94%)	25 (100%)	25 (89%)	
Yes	3 (6%)	0 (0%)	3 (11%)	

Post-HCT IL15-CIK (shaded), IL15-activated cytokine-induced killer cells; HCT, hematopoietic stem cell transplantation; AML, acute myeloid leukemia; CML, chronic myeloid leukemia; CMML, chronic myelomonocytic leukemia; MRC, myelodysplasia-related changes; cALL, common acute lymphoblastic leukemia; T-ALL, T-cell ALL, CR, complete remission; NR, not in remission; MD, matched donor; MMD, mismatched donor; MSD, matched sibling donor; MUD, matched unrelated donor; MMFD, mismatched family donor; MMUD, mismatched, unrelated donor; BM, bone marrow; PBSC, peripheral stem cells; ATG, anti-thymoglobulin; CT, clinical trial; PS, pilot study. Testing was performed for differences between ≤8/10-HLA-matched donors and ≥9/10-HLA-mismatched donor IL15-activated cytokine-induced killer cells.

Supplementary Table 10
Case Characteristics of the Consolidation Cohort

Age (years) / Gender P-Index (%)	Disease status at HCT	HCT Number / Remission status	Time from diagnosis to HCT (years)	Conditioning regimen	Donor HLA-match	Female donor to male patient	Graft Manipulation Composition $CD34^+$/kg	Serotherapy	Time from HCT to 1st IL15-CIK (days)	Age (years) P-Index % at 1st IL15-CIK
17, f 100%	AML M5, MLL/AF10, EMD: vitreous body infiltration	2nd HCT, CR2	1.2	Clo, Cy, Eto, Flu, Thio, Mel	MMFD	No	PBSC 3/19depl. 7.1 Mio.	Campath	64	17, 80%
7, m 70%	AML M2, Complex karyotype 2nd relapse	4th HCT, NR (aplasia)	2.0	Flu	MMFD	No	PBSC 3/19depl. 3.8 Mio.	Campath	56	7, 70%
13, f 100%	AML, FLT3-ITD, EMD: CNS infiltration	1st HCT, CR2	12.5	Clo, Cy, Eto, Flu, Thio, Mel	MMFD	No	PBSC 3/19depl. 12.1 Mio.	Campath	103	14, 90%
9, m 100%	cALL, 3rd relapse	2nd HCT, NR	7.8	Clo, Daunoxome, Ara-C, Flu, Thio, Mel	MMFD	No	PBSC 3/19depl. 18.6 Mio.	Campath	34	9, 100%
1, m 100%	Pro-B-ALL, KMT2A (former MLL)	2nd HCT, CR2	1.5	Clo, Cy, Eto, Flu, Thio, Mel	MMFD	No	PBSC 3/19depl. 31.1 Mio.	Campath	44	2, 100%
15, m 100%	ALL, TEL-AML, 2nd relapse	2nd HCT, NR	12.9	Clo, Cy, Eto, Flu, Thio, Mel	MMFD	Yes	PBSC 3/19depl. 11.6 Mio.	Campath	50	15, 90%
17, m	ALL, STIL-TAL1rr CDKN2A del, 1st relapse	1st HCT, NR	0.7	Flu, Thio, Mel	MMFD	No	PBSC, TCR α/β/ CD19dep, 11.9 Mio.	ATG	27	17

Heavily pretreated cases who received IL15-CIK therapy as post-remission consolidation treatment, i.e., while in complete molecular remission (CMR) after allogeneic hematopoietic cell transplantation (HCT), with the intent to strengthen disease control are shown.

P-Index, performance status; HCT, hematopoietic stem cell transplantation; AML, acute myeloid leukemia; ALL, acute lymphoblastic leukemia; EMD, extramedullary disease; CR, complete remission; NR, not in remission; Clo, Clofarabine; Daunoxome, Daunorubicin liposomal; Ara-C, Cytarabine; Flu; Fludarabine; Thio, Thiotepa; Mel, Melphalan; Cy, Cyclophosphamide; Eto, Etoposide; HLA, human leukocyte antigen; MMFD, haploidentical family donor; PBSC, peripheral blood stem cells; ATG, anti-thymocyte globulin; IL15-CIK, IL-15-activated cytokine-induced killer cells.
