# Supplementary material for: First-in-Human Study of IL15–Activated Cytokine-Induced Killer Cells After Allogeneic HCT Shows Durable Remission and Serotherapy-Associated Immune Reconstitution in Leukemia
Source: J Clin Oncol. 2026 Apr 6;44(14):1323–36. doi: 10.1200/JCO-25-01966 (PMC13166077; doi:10.1200/JCO-25-01966)
Supplement: Supplementary file 3 [file jco-44-1323-s003.pdf]

Study protocol

Title of the Study:

A prospective phase I/II study to investigate the feasibility, safety and efficacy of IL-15 activated cytokine induced killer (CIK) cells in relapsing patients with acute leukemia or myelodysplastic syndromes after allogeneic stem cell transplantation

Protocol Code Number: **FFM - CIK-Cell Study 01**

Protocol Version: **2.0**

Protocol Version Date: **01.02.2015**

EudraCT Nummer: **2013-005446-11**

Sponsor:
Goethe-University
Represented by the President
Grüneburgplatz 1, 60323 Frankfurt am Main
Phone: +49 (69) 798-11103
Fax: +49 (69) 798-11109
Email: burtscheidt@pvw.uni-frankfurt.de

Coordinating Study Center:

University Hospital Frankfurt, Goethe University
Department for Children and Adolescents
Director Prof. Dr. med. Thomas Klingebiel
Division for Stem Cell Transplantation and Immunology
Head Prof. Dr. med. Peter Bader
Theodor-Stern-Kai 7, 60590 Frankfurt am Main, Germany
Phone: +49 (69) 6301-7542
Fax: +49 (69) 6301-4202

Principal Investigator:
Prof. Dr. med. Peter Bader
University Hospital Frankfurt, Goethe University
Department for Children and Adolescents
Division for Stem Cell Transplantation and Immunology
Theodor-Stern-Kai 7, 60590 Frankfurt am Main, Germany
Phone: +49 (0) 696301-7542
FAX: +49 (0) 696301-4202
E-mail: peter.bader@kgu.de

Study Centers:

University Hospital Frankfurt, Goethe University
Department for Children and Adolescents
Director: Prof. Dr. med. Thomas Klingebiel
Division for Stem Cell Transplantation and Immunology,
Head: Prof. Dr. med. Peter Bader
Theodor-Stern-Kai 7, 60590 Frankfurt am Main, Germany
Phone: +49(0)696301-7542
Fax: +49(0)696301-4202

Internal Medicine II,
Department of Hematology, Oncology, Rheumatology and Infectious Diseases, Goethe-University Frankfurt/Main,
Theodor-Stern-Kai 7, Frankfurt/Main, Germany,
Director: Prof. Dr. med. Hubert Serve
Theodor-Stern-Kai 7, 60590 Frankfurt am
Main, Germany
Phone: +49(0)69 6301-5194
Fax: +49(0)69 63017326

Internal Medicine III,
Department of Hematology and Oncology, Johannes Gutenberg University,
Director: Prof. Dr. med. Matthias Theobald,
Langenbeckstr. 1
55101 Mainz, Germany
Phone: +49(0)6131 175 047
Fax: +49(0)6131 393 3364

Cell Processing:

Institute for Transfusion Medicine and Immunhematology
German Red Cross Blood Donation Center Baden-Württemberg-Hessen
Goethe University, Frankfurt am Main, Germany
Head: Prof. Dr. med. E. Seifried
Division for Cell Processing
Head: Prof. Dr. med. H. Bönig
Sandhofstr. 1, 60528 Frankfurt am Main, Germany

Laboratories:

University Hospital Frankfurt, Goethe University
Department for Children and Adolescents
Division for Stem Cell Transplantation and Immunology
Stem Cell Laboratory (House 32D)
Theodor-Stern-Kai 7, 60590 Frankfurt am Main, Germany
Phone: +49 (0) 696301-4918
Fax: +49 (0) 696301-83168

University Hospital Frankfurt, Goethe University
Department for Children and Adolescents
Division for Stem Cell Transplantation and Immunology
Chimerism Laboratory (House 32A)
Theodor-Stern-Kai 7, 60590 Frankfurt am Main, Germany
Phone: +49 (0) 696301-6449
Fax: +49 (0) 696301-83937

Sponsor: Goethe-University
Represented by the President
Grüneburgplatz 1, 60323 Frankfurt am Main
Phone: +49 (69) 798-11103
Fax: +49 (69) 798-11109
Email: burtscheidt@pvw.uni-frankfurt.de

Statistician: Emilia Salzmann-Manrique

Coordinating physician: Dr. med. Eva Rettinger

Signatures

The following persons agree with the content of the clinical study and confirm the protocol latest version.

Sponsor
Goethe-University, represented by the President

Date Signature

Principal Investigator
Prof. Dr. med. Peter Bader, University Hospital Frankfurt, Goethe University, Department for Children and Adolescents, Division for Stem Cell Transplantation and Immunology

Date Signature

Statistics
Emilia Salzmann-Manrique, University Hospital Frankfurt, Goethe University, Department for Children and Adolescents

Date Signature

Director
Prof. Dr. med. Thomas Klingebiel, Director University Hospital Frankfurt, Goethe University, Department for Children and Adolescents

Date Signature

Investigator's Protocol Approval Signature

By my signature, I confirm to have read the study protocol completely and I ensure the conduct of the clinical study in compliance with the protocol, ICH-GCP and the applicable national and European regulations covering the conduct of clinical studies.

Investigator

Name (print):

____

Study Center:

____

____

____

Date Signature

Notice of confidentiality

This study protocol is property of the University Hospital Frankfurt, Goethe University, Department for Children and Adolescents, Division for Stem Cell Transplantation and Immunology.

All information is confidential and cannot be disclosed without previous written authorization of the principal investigator.

Table of Contents

Synopsis ... 9
Glossary of Abbreviations ... 15
1 Introduction ... 17
1.1 Allogeneic Stem Cell Transplantation and Cellular Therapy ... 17
1.2 Differences between adult and pediatric patients with acute and chronic leukemia and with MDS ... 18
1.3 Cytokine Induced Killer (CIK) Cells ... 18
1.4 Pre-clinical Data ... 19
1.5 Clinical Data ... 19
2 Aims of the Study ... 21
2.1 Primary Objectives ... 21
2.2 Secondary Objectives ... 21
2.3 Primary Endpoints ... 21
2.4 Secondary Endpoints ... 22
2.5 Further Analysis ... 22
3 Study Design ... 22
3.1 Study Procedure ... 23
3.2 Patient Evaluation - Assessments ... 24
3.3 Study Duration ... 29
3.4 Number of Patients ... 29
4 Study Population ... 29
4.1 Inclusion Criteria ... 30
4.2 Exclusion Criteria ... 30
4.3 Allowed/prohibited concomitant therapies ... 31
5 Investigational Medicinal Poduct - CIK cells ... 31
5.1 CIK Cell Donors ... 31
5.2 Donor Exclusion Criteria ... 31
5.3 CIK Cell Generation ... 32
5.4 CIK Cell Recipient ... 32
5.5 Benefit-Risk-Assessment ... 32
6 Safety Issues ... 32
6.1 Definitions ... 32

6.2 Documentation and Reporting of serious adverse events 34
6.3 Documentation and reporting of suspected unexpected serious adverse reactions (SUSARS) 34
6.4 Annual safety reports 35
7 Ethics and General Study Administration 35
7.1 Ethical Aspects and Independent Ethics Committee (IEC) 35
7.2 National Competent Authority (NCA) 35
7.3 Notification of the Study 36
7.4 Insurance 36
7.5 Financing 36
7.6 Consent procedure 36
7.7 Amendments 37
7.8 Data Protection 37
8 Discontinuation or Early Termination of the Study 37
8.1 Criteria for Discontinuation of the Treatment or Premature Withdrawal of the Patient 37
8.2 Criteria for Discontinuation of Termination of the Study 38
9 Publications Rules and Results 38
10 Monitoring 39
11 Statistics 40
12 Literature 45
13 Appendices 47

Synopsis

**Title of study**	A prospective phase I/II study to investigate the feasibility, safety and efficacy of IL-15 activated cytokine induced killer (CIK) cells in relapsing patients with acute leukemia or myelodysplastic syndromes after allogeneic stem cell transplantation
**Protocol short title /acronym**	CIK cell intervention after allogeneic stem cell transplantation
**Protocol code number:**	FFM-CIK-Cell Study 01
**EudraCT number:**	2013-005446-11
**Coordinating investigator**	Prof. Dr. med. Peter Bader<br>University Hospital Frankfurt, Goethe University<br>Department for Children and Adolescents<br>Division for Stem Cell Transplantation and Immunology, Germany
**Planned number of sites**	3 sites in Germany
**Design**	Multi-site, non-randomized Phase I/II study involving children and adults.<br>This is a phase I/II multicenter-study to investigate the feasibility safety and efficacy of interleukin (IL)-15 activated CIK cells in patients with acute leukemia or myelodysplastic syndrome (MDS) showing evidence of relapse after allogeneic stem cell transplantation (SCT). CIK cell infusions will be given with an interval of 4-6 weeks according to a dose escalation schedule in patients with impending relapse after allogeneic SCT. In presence of acute graft versus host disease (aGvHD) ≥ grade II, the next scheduled infusion will not be administered.
**Medical condition or disease under investigation**	In this phase I/II study, increasing doses of CIK cell infusions will be offered to adult and pediatric patients with acute leukemia or myelodysplastic syndrome (MDS) showing evidence of molecular relapse after allogeneic SCT.
**Objectives**<br>**Primary objectives**	To assess the safety and feasibility of increasing cell doses of CIK cell transfusions in adult and pediatric leukemia and MDS patients with molecular relapse after allogeneic SCT. The occurrence of grade III or IV aGvHD and extensive chronic GvHD (cGvHD) will be judged being related to the CIK cell intervention. Grading and staging will be performed using the Glucksberg scale (GSC).
**Secondary objectives**	To assess the efficacy of CIK cell treatment based on:<br>• Efficacy will be analyzed by progression free survival and overall survival.<br>• Reduction or disappearance of minimal residual disease (MRD),<br>• Achievement of complete donor chimerism (CC),

	• Rate of and time to hematological relapse in patients enrolled based on MRD and/or mixed chimerism (MC), and<br>• Rate and duration of complete and partial molecular response following CIK cell infusion.
**Study endpoints**<br>**Primary endpoints**	• The dose-limiting toxicity based on grade III or IV aGvHD, and<br>• Extensive cGvHD
**Secondary endpoints**	Assessment after CIK cell infusion:<br>• Molecular response will be assessed as efficacy end point.<br>• Progression free survival will be assessed 1 year after initiation and the end of CIK cell therapy<br>• Overall survival will be assessed 1 year after initiation and the end of CIK cell therapy.<br>• Immune reconstitution will be assessed by screening for CD3-, CD4-, CD8-, CD56-, CD14-positive cells.<br>Assessment during CIK cell infusion:<br>• Cytokine secretion will be assessed during CIK cell infusion and thereafter.<br>• Tumor escape mechanisms including detection of soluble ligands of NKG2D receptor will be assessed following CIK cell infusions.
**Number of patients**	40 patients (20 pediatric and 20 adult patients)
**Study population**	Acute leukemia and MDS patients of both gender aged > 0 and < 80 years experiencing molecular relapse after allogeneic SCT.
**Inclusion criteria**	• Acute leukemia and MDS patients with molecular relapse in peripheral blood (PB) or bone marrow (BM) samples obtained during monitoring for relapse after allogeneic SCT.<br>1. MRD detected by Ig/TCR gene rearrangements testing or<br>2. confirmed mixed chimerism (MC) ≥ 1%, or<br>3. levels ≥ 10^{-4} of BCR-ABL/ABL ratio will trigger CIK cell interventions<br>• Respecting MC, MC ≥ 1% of autologous signals in PB samples confirmed by another PB or BM sample within one week. Patients with MC ≥ 1% of autologous signals in $CD33^+$ and/or $CD34^+$ subpopulations in PB samples confirmed by BM analyses within one week. Acute leukemia and MDS patients with MC ≥ 1% of autologous signals including signals in $CD33^+$ and/or $CD34^+$ subpopulations in BM samples.<br>• Patients without immunosuppressive agents and steroids.<br>• Patients without chemo- or immune therapy, except patients with thyrosine-kinase inhibitors (TKI) for treatment of BCR-ABL positive leukemias.

	• Patients with < grade II GvHD.<br>• Patients with Karnowsky or Lansky performance status ≥ 50%.<br>• Patients and/or his/her legal representative having reviewed the patient information/informed consent form and have had their questions answered and have given written informed consent.
**Exclusion criteria**	• Acute leukemia and MDS patients with hematologic relapse ≤ day 120 after allogeneic stem cell transplantation<br>• Patients with more than 5% malignant cells in bone marrow analyses<br>• Patients with immunosuppressive agents or steroids.<br>• Patients with chemo- or immune therapy, except patients with thyrosine-kinase inhibitors (TKI) for BCR-ABL positive leukemias<br>• Patients with ≥ grade II GvHD.<br>• Patients with Karnowsky or Lansky performance status < 50%.<br>• Patients and/or his/her legal representative having reviewed the patient information/informed consent form and have had their questions answered and have not given written informed consent.<br>• HIV-positive patients.<br>• HBV/HCV patients.<br>• Patients with prior solid organ transplantation.<br>• Patients treated with any other investigational product within the last 28 days or five half-lives (whichever is longer).<br>• Hypersensitivity to any component of the study drug<br>• Female patients of child-bearing potential not agreeing to use a highly effective method of birth control resulting in a low failure rate (i.e. < 1%) when used consistently and correctly.<br>• Male patients with female partners of childbearing potential not agreeing to use a highly effective method birth control resulting in a low failure rate (i.e. < 1%) when used consistently and correctly.<br>• Pregnancy/Breastfeeding.<br>• Patients with severe infections or signs/symptoms of infection within 2 weeks prior to study start.
**Duration of patient participation/ duration of the study**	Patient recruitment: 3 years<br>Study duration per patient: Includes study phase during CIK cell infusions and observational phase after the last CIK cell infusion<br>Study phase: Will last until the patient reaches complete or molecular remission = end of CIK cell

	infusions or received a maximum of 8 infusions = end of CIK cell infusions<br>(maximum duration of study phase = 1 year)<br>Observational phase (follow up): will last at least 1 year after the last CIK cell infusion<br>End of study: 5 years
**Definition of end of trial**	The end of trial will be one year after last patient receives last infusion of CIK cells.
**Investigational Medicinal Product:**	IL-15 activated CIK cells individually generated from PB mononuclear cells of the original stem cell donors. CIK cells represent a heterogeneous population of polyclonal T and natural killer (NK) cells. T cells in part share NK phenotype and functional properties of NK cells.<br>CIK cells in general can be expanded *in vitro* from PB, BM mononuclear cells and umbilical cord blood by addition of interferon (IFN)γ, activating antibody directed against CD3, and IL-2 followed by addition of IL-15 for further CIK cell activation and expansion. IL-15 expansion lasts 10 days.

**Activities/Examinations**	Screening for eligibility			**First CIK cell infusion**			**2nd to 8 or last[7] -CIK cell infusion** (interval between infusions: 4-6 weeks) Follow up after infusions see 1st CIK cell infusion					**Follow up after last CIK cell infusion** within 1 month and thereafter every 3 months
**Weeks**				**0**	**1**	**2-4**	**4-6**	**5-7**	**6-8**	**…**	**…**	
Written Informed Consent	x											
Medical history/ Transplantation history	x											
Concomitant medication	x											
Inclusion/Exclusion Crit.	x											
Physical examination	x			x		x						x
GvHD acc. to Glucksberg Seattle Criteria	x					x						
Lansky/Karnofsky index	x					x						x
Body weight	x					x						
Cell infusion				*x*[1]			x[1]					
ECG, pulse and O_2 oxygenation, while and 60 min after CIK cell infusion				x			x					
Blood pressure Every 10 minutes while and 60 minutes after infusion				x			x					
Infusion related toxicity				x[1]			x[1]					
Peripheral Blood												
Blood count	x			X[2]	x	x	x	x	x			x
Bilirubin (total)	x			x		x			x			x

Immune status	(x)			x^2	x^2	x^2	x^2	x^2	x^2			(x)

Cytokine profile				x^2	x^2	x^2	x^2	x^2	x^2			
NKG2D receptor				x^2	x^2	x^2	x^2	x^2	x^2			
Chimerism	x					x^3			x^3			x
BCR-ABL	(x)					(x)			(x)			
Bone marrow assessments												
Morphology/Histology	x					x^4						$(x)^5$
Chimerism	x					x^4						$(x)^5$
Chimerism Subpop	(x)					x^4						$(x)^5$
MRD or	(x)					x^4						$(x)^5$
BCR-ABL	(x)					x^4						$(x)^5$
Ophtalmologist control												x^6
Pulmonary function testing												x^6

Legend:
(), if applicable/optional
x^1, monitoring for acute transfusion related toxicity while and up to 12 hours after CIK cell infusion, patients should be observed overnight within the context of an inpatient hospital stay. The study drug must be administered where emergency resuscitative equipment and personnel trained in the management of anaphylaxis are immediately available to treat systemic reactions under the direct supervision of a physician
x^2, blood samples before, 1 hour, 1 and 2 weeks after CIK cell infusion for analyses of cytotoxic effects and tumor escape mechanisms
x^3, monthly obligatory
x^4, every 3 months
x^5, optional
x^6, once a year
[7], stop of CIK cell infusions: in case of molecular remission or if discontinuation criteria are reached,
If the patient does not reach molecular remission, administration of CIK cells will be stopped after the 8th CIK cell infusion.

Glossary of Abbreviations

aGvHD	-	Acute Graft-versus-Host Disease
AE	-	Adverse Event
ALL	-	Acute Lymphoblastic Leukemia
Allo	-	Allogeneic
AML	-	Acute Myelogenous Leukemia
BM	-	Bone Marrow
CC	-	Complete Chimerism
cGvHD	-	Chronic Graft-versus-Host Disease
CIK	-	Cytokine induced killer
DFS	-	Disease free survival
DLI	-	Donor Lymphocyte Infusion
ECG	-	Electrocardiography
GMP	-	Good Manufacturing Process
GSC	-	Glucksberg Seattle Score
GvHD	-	Graft versus Host Disease
HBs AG	-	Hepatitis s Antigen
HCV	-	Hepatitis C Virus
HIV	-	Human Immunodeficiency Virus
HLA	-	Human Leukocyte Antigen
IFN	-	Interferon
Ig	-	Immunoglobuin
IL	-	Interleukin
IMP	-	Investigational Medicinal Product
IZKS	-	Interdisziplinäres Zentrum Klinische Studien
KIR	-	Killer Cell Immunoglobulin Like Receptor
MC	-	Mixed Chimerism
MDS	-	Myelodysplastic syndrom
MHC	-	Major Histocompatibility Complex
MRD	-	Minimal Residual disease
NK	-	Natural killer
NKG2D	-	Activatory NK Cell Receptor
PB	-	Peripheral Blood
PBMC	-	Peripheral Blood Mononuclear Cells
PCR	-	Polymerase Chain Reaction

RMS	-	Rhabdomyosarcoma
RT	-	Reverse Transcriptase
SAE	-	Severe Adverse Event
SCT	-	Stem cell transplantation
STR	-	Short Tandem Repeats
SUSAR	-	Suspected Unexpected Serious Adverse Reactions
TCR	-	T cell receptor
TPHA	-	Treponema pallidum Haemagglutinantionstest
TRM	-	Treatment Related Mortality
TKI	-	tyrosine kinase inhibitor
WT	-	Wilms Tumor

Surface Antigenes

CD3	-	T Cell
CD4	-	Helper T Cell
CD8	-	Cytotoxic T Cell
CD14	-	Monocyte
CD34/ 33	-	Stem cell
CD56	-	NK Cell

1 Introduction

1.1 Allogeneic Stem Cell Transplantation and Cellular Therapy

In acute lymphoblastic (ALL) and myeloid (AML) leukemia patients as well as in adult myelodysplastic syndrome (MDS) patients with chemotherapy resistant diseases allogeneic hematopoietic stem cell transplantation (SCT) will be performed according to the respective study protocols. Moreover, allogeneic SCT includes upfront therapy for pediatric patients with MDS, and will also be offered for therapy-related MDS and AML, which might occur as complications of chemotherapy. This complication often presents after a median latency period of approximately four to five years after chemotherapy treatment.

Standard therapy of BCR-ABL positive leukemias above all includes treatment with tyrosine-kinase inhibitors (TKI).

Hence, allogeneic SCT has become an established method in the treatment of above-mentioned high-risk leukemia and myelodysplastic syndromes (MDS). In both groups, the therapeutic success is limited by patients' relapse. Once a stem cell recipient relapses, further therapeutic options are limited. For instance, re-induction chemotherapy may cause remission with acceptable toxicity and a possible delay in time to relapse but therapeutic effects are transient. Relapsed patients with good performance status and without severe comorbidity might be considered for a second allogeneic SCT, if relapse occurs after day 300 in adult and after day 200 in pediatric patients. In these patients, second allogeneic SCT may provide a small curative chance of 20% but this approach entails a high rate of morbidity and mortality.

Another treatment option may include taper of immunosuppressive medications followed by donor lymphocyte infusion (DLI), which can generate a graft versus leukemia effect (GvL), but is associated with an increased risk of graft versus host disease (GvHD). Moreover, the success rate of this approach is less than 15%.

To improve outcome of leukemia and MDS patients after allogeneic SCT, the detection of impending relapse or early stages of relapse is necessary, as this status is a precondition for further therapeutic options. Predictors of relapse after allogeneic SCT have been monitoring for minimal residual disease (MRD), and BCR-ABL as well as loss of donor cell chimerism.

Evidence was presented, that MRD or MC status in leukemia and MDS patients in principle could be treated by DLI. However, DLI is only efficacious for defined diseases and is of limited value when initiated during overt relapse. Moreover, the high T cell doses required for DLI raise the risk of GvHD.

Due to the risk of GvHD, strategies to prevent post-SCT relapse have been developed including vaccination and targeted immunologic interventions (i.e. small molecule inhibitors, mAbs and chimeric antigen receptor T cells). These treatments have mostly been evaluated in vitro or in early phase I/II studies and require the presence of leukemia specific antigens. In contrast, cytokine induced killer (CIK) cells with reduced alloreactive potential are broadly applicable against a variety of hematological malignancies including ALL, AML and MDS. Sequential infusions of interleukin (IL)-15 expanded CIK cells may therefore prevent relapse with reduced risk of causing GvHD in patients with leukemia or MDS being at high risk for relapse after allogeneic SCT. As immunotherapeutic intervention is most promising in the setting of low leukemic cell burden CIK cells will be administered in patients with impending relapse indicated by MRD, BCR-ABL or MC in this trial.

1.2 Differences between adult and pediatric patients with acute and chronic leukemia and with MDS

Treatment and prognosis of patients with leukemia and MDS differs significantly between children and adolescents and adult patients. As a consequence the prognosis in childhood is by far better than in adulthood. In the medical community it is generally accepted that children are not small adults.
The physiology of children differs from that of adult individuals fundamentally. This is true on all aspects of metabolism. Children are tolerating all kind of drugs better than adult patients. Half-life time of drug excretion is different between children and adults. This is also true for example in the antibody immunotherapy. It could be shown in this field that also the response rate to the drug is different between children and adults; in this time in favor for adult patients.

This proofs that leukemic blasts are frequently different between juvenile and adult leukemia cells. Regularly childhood leukemia and MDS is more susceptible for all kind of treatment. Therefore treatment schedules can be different and as a consequence for what is learned from treating adult patients only little is transferable to the treatment of children.

This is in particular true in the setting of allogeneic stem cell transplantation. In this field the differences in physiology of both patient groups is apparent. So is the rate of acute and chronic GvHD completely different. The rate of severe GvHD is markedly reduced in pediatric patients. This is important when developing optimal dosing or optimal timely scheduling of immunotherapy with the potential of inducing GvHD as this is the case in the current study using cytokine induced killer cells.

Consequently, knowledge obtained through the treatment of adult patients cannot be transferred to the treatment of pediatric patients. In addition, there is no gold standard treatment for patients, neither for children nor for adults with acute leukemia and MDS who are relapsing after allogeneic stem cell transplantation. So, there is a unmet need for developing strategies for rescuing these patients. The current treatment option with CIK cells is yet such an option for both, children and adults.

1.3 Cytokine Induced Killer (CIK) Cells

Activated CIK cells represent a heterogeneous population of polyclonal T and natural killer (NK) cells. T cells in part share both NK phenotype and functional properties of NK cells. CIK cells can be efficiently expanded *in vitro* from peripheral blood (PB), bone marrow (BM) mononuclear cells and umbilical cord blood by addition of interferon (IFN)γ, activating antibody directed against CD3, IL-2 and as a new strategy using IL-15 for further CIK cell activation and expansion.
CIK cells are known in principle to be capable of eradicating a variety of both hematological and solid malignancies in a non-major histocompatibility complex (MHC)-restricted manner, without possessing significant alloreactive potential. In recent years, the application of CIK cells has evolved from experimental observations into early clinical studies. Introna, Laport, Linn and colleges described application of

donor-derived CIK cells in adult patients with relapsed hematological malignancies after allogeneic SCT.

1.4 Pre-clinical Data

In previous work we generated CIK cells *in vitro* using published protocols. We demonstrated potent cytotoxic capacity of conventional IL-2 stimulated CIK cells against soft tissue sarcoma cells *in vitro*. We recently used IL-15 for further CIK cell activation and expansion. We could show that IL-15-activated CIK cells have an increased anti-leukemic, anti-lymphoma and anti-tumor potential *in vitro* compared to conventional IL-2 activated CIK cells. Furthermore, our modified protocol allowed us to shorten *ex vivo* expansion time of CIK cells.
In vitro analysis of IL-15 activated CIK cells demonstrated that activating NK cell receptor NKG2D is apparently involved in the recognition of target cells whereas killer-cell immunoglobulin-like receptor (KIR)-HLA mismatches contributed to a lesser extent to the CIK cell-mediated cytotoxicity. In addition,$CD56^-$ CIK cell subpopulations were more effective in the lysis of acute myeloid leukemia (AML) cells, in contrast to $CD56^+$ CIK cells, which showed the highest cytotoxic potential against acute lymphoblastic leukemia (ALL) cells. In contrast, no alloreactivity was observed against allogeneic peripheral blood mononuclear cells (PBMC) and fibroblasts.
To generate pre-clinical data, we furthermore developed a mouse model to study anti-leukemic and anti-tumor potential of CIK cells *in vivo*. Immunodeficient mice (NOD/SCID/$IL2R\gamma c^-$, NSG) were injected intravenously with human AML cell lines with primary AML cells and with human rhabdomyosarcoma (RMS) cell lines at minimal doses required for engraftment of malignant cells. Mice transplanted with malignant cells were randomly assigned for analysis of CIK cell treatment. CIK cell treated mice showed a significantly increased disease free survival (DFS) compared with untreated controls. Histological analysis of GvHD-targeted organs, i.e. gastrointestinal tract, spleen and lung showed minimal xenogenic GvHD after CIK cell treatment.

1.5 Clinical Data

Based on our pre-clinical data, we analyzed safety and feasibility of a sequential, dose escalating administration of *in vitro* expanded CIK cells in patients with hematological malignancies after allogeneic SCT.

Our experience of repetitive, dose-escalating CIK cell infusions include 14 leukemia patients with risk of relapse (n=11) or overt relapse (n=3) showing no signs of acute GvHD >grade I after allogeneic SCT.

Between August 11, 2011 and November 30, 2014 14 patients (<18 years of age, n=13, median age 9, range 1-17 years; >18 years of age, n=1, age 69 years) with hematological malignancies (AML, n=7; ALL, n=6; CML, n=1) received a total of 58 CIK cell infusions (median number 3, range 1-10 per patient) from matched unrelated (n=6) or haploidentical (n=8) stem cell donors at a minimum of 3 weeks after allogeneic SCT and an interval of 4-6 weeks between infusions (median follow up after 1st CIK cell infusion 213 days, range 56-802 days). 6 patients received CIK cell infusions based on minimal residual disease or chimerism status, 5 patients, most of whom were not in remission at the time of allogeneic SCT, were treated prophylactically, and 3 relapsed patients received CIK cell infusions with or without chemotherapy.

Clinical batches of CIK cells were generated from peripheral blood mononuclear cells of the original stem cell donors under good manufacturing practice-conditions in the presence of interferonγ, anti-CD3 antibody, interleukin (IL)-2 and IL-15. Cellular composition of CIK cell infusions showed that $CD19^+$ B cells, $CD14^+$ monocytes and $CD3^-CD56^+$ natural killer (NK) cells were quantitatively depleted while conventional $CD3^+CD56^-$ T cells and $CD3^+CD56^+$ T-NK cells made up the bulk of the CIK cell product. T cells, evenly distributed between the $CD4^+$ and $CD8^+$ compartment, were activated, which was shown by $CD25^+$ expression and were mostly TCRα/β type.

Starting T cell dose within CIK cell infusions was $1x10^6$ T cells/kg body weight. Regardless of donor type, dose escalation continued up to $1x10^8$ T cells/kg body weight if no signs of acute GvHD >grade I occurred. Median doses of infused T cells were $6x10^7$ in the matched unrelated and $1x10^7$/kg in the haploidentical transplantation setting. Mild signs of acute GvHD occurred in 2 patients (14%).

Another 3 patients (21%) developed acute GvHD grade III with evidence of cutaneous, intestinal and hepatic involvement. All of these high-risk patients had been treated with CIK cells from haploidentical donors prophylactically at a maximum dose of $5x10^6$, $5x10^6$ and $20x10^6$ $CD3^+CD56^-$ CIK cells/kg body weight. All patients experienced a timely coincident regeneration of $CD3^+$ T cells. Therefore it is not clear whether the occurrence of GvHD is solely attributable to CIK cell infusions.Treatment for acute GvHD included immunosuppressive medications in addition to steroid therapy and infusions of mesenchymal stroma cells. One patient additionally received extra corporal photopheresis. All patients finally recovered from GvHD and remained in complete remission until the last follow-up.

In 6 patients (43%), 3 with overt and 3 with impending relapse, CIK cell infusions temporary provided stable disease (median 218 days, range 56-563 days), followed by another allogeneic SCT in 2 cases. Deaths of 2 heavily pretreated AML-patients (14%) who remained in remission after CIK cell infusions were not CIK cell-related. Another 6 leukemia patients (43%), 3 of whom were not in remission at the time of SCT, are still in complete remission. All of them were offered CIK cell infusions from haploidentical donors.

Accordingly, Introna, Laport, Linn and colleges described application of donor-derived CIK cells in adult patients with relapsed hematological malignancies after allogeneic SCT. Altogether, serial CIK cell infusions seem to be a promising therapeutic approach for patients being at risk of relapse, provided that CIK cells are administered in high numbers and short-term intervals, early after allogeneic SCT.

2 Aims of the Study

During the last decade, substantial progress has been made in the field of stem cell transplantation. Nevertheless, especially in patients with acute leukemia and MDS, relapse remains the major cause for treatment failure. In case of hematologic relapse, additional treatment is limited to either second SCT or preemptive immunotherapy including donor lymphocyte infusion. Second allogeneic SCT may provide a small curative chance but this approach entails a high rate of morbidity and mortality. DLIs are of limited value when initiated during overt hematological relapse and the high cell doses needed increase the risk of developing severe GvHD. It has been shown that in addition to detectable levels of MRD, impending relapse can be predicted by mixed chimerism (MC). As immunotherapeutic intervention is most promising in the setting of low leukemic cell burden, administration of *ex vivo* activated CIK cells in patients with MRD or MC due to their low risk for GvHD may represent an improved treatment option compared to conventional DLIs.

This intervention trial will be performed to define the safety profile and to evaluate the efficacy of sequential, dose escalating CIK cell infusions for the treatment of molecular relapse after allogeneic SCT including progression free survival and overall survival.

2.1 Primary Objectives

- To assess the safety and feasibility of increasing cell doses of CIK cell transfusions in adult and pediatric leukemia and MDS patients with molecular relapse after allogeneic SCT. The occurrence of grade III or IV acute GvHD and extensive chronic GvHD will be judged being related to the CIK cell intervention. Grading and staging will be performed using the Glucksberg Seattle scale.

2.2 Secondary Objectives

To assess the efficacy of CIK cell treatment based on:

- Reduction or disappearance of MRD
- Achievement of complete donor chimerism,
- Rate of and time to hematologic relapse in patients enrolled based on MRD and/or mixed chimerism
- Progression-free and overall survival
  Molecular relapse will be assessed by STR and RT-PCR based chimerism analyses in all patients, by real time (RT)-PCR based BCR-ABL/ABL, or immunoglobulin (Ig)/T cell receptor (TCR) gene rearrangements in leukemia patients

2.3 Primary Endpoints

- The dose-limiting toxicity based on grade III or IV acute GvHD
- Chronic extensive GvHD

2.4 Secondary Endpoints

- Molecular response as efficacy end point.
- Progression free survival at 1 year after initiation and the end of CIK cell therapy.
- Overall survival will be assessed 1 year after initiation and the end of CIK cell therapy.

2.5 Further Analysis

- To assess improvement of hematologic and immune reconstitution after CIK cell infusions (leucocyte and platelet counts and numbers of CD3-, CD4-, CD8-, CD56-, CD14- positive cells).
- To assess the cytokine profile during CIK cell infusion and thereafter.
- To assess tumor escape mechanisms following CIK cell infusions, including detection of soluble ligands of NKG2D receptor.

3 Study Design

This is a phase I/II study to investigate the feasibility, safety and efficacy of IL-15 activated CIK cells in patients with acute leukemia or MDS showing evidence of molecular relapse after allogeneic SCT.

- CIK cell infusions will be given with an interval of 4-6 weeks according to a dose escalation schedule in patients with impending relapse after allogeneic SCT. In presence of aGvHD ≥ grade II, the next scheduled infusion will not be administered. CIK cell infusions will be administered until the patient reaches molecular remission, until a maximum of 8 infusions are reached or until the patient progresses, provided that patients develop no more than grade I aGvHD.

3.1 Study Procedure

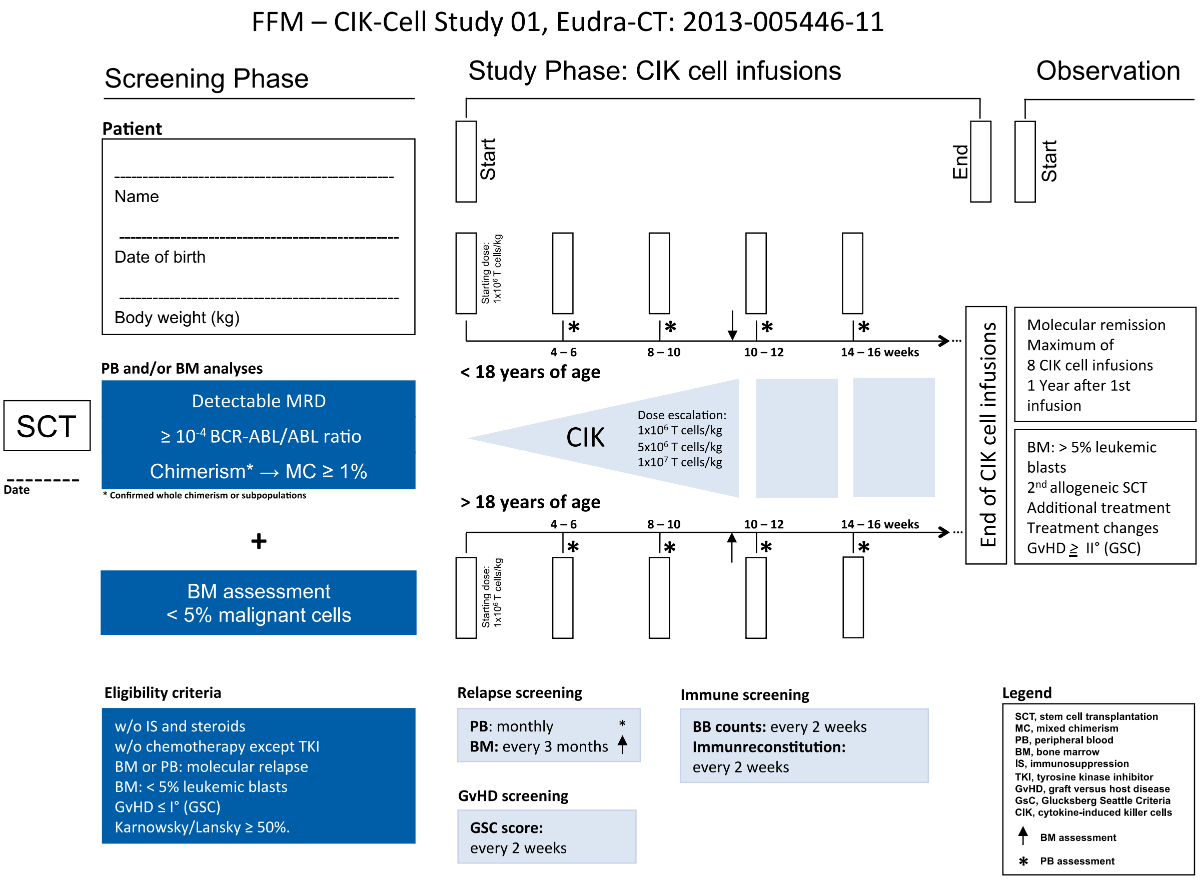

Figure 1: Trial scheme (new)

In this phase I/II study, adult and pediatric patients with acute leukemia or MDS after allogeneic SCT will be screened for relapse according to general practice including chimerism analyses.

- In patients with impending relapse indicated by MRD levels or MC ≥ 1% including chimerism subpopulations, leukemic blast must be below 5% of cells in bone marrow samples for eligibility.

- Having reached all required inclusion criteria, patients will receive first dose of CIK cell infusions.

- According to risk for GvHD, starting dose will be 1 x 10^6 $CD3^+CD56^-$ CIK cells/kg in pediatric as well as in adult patients.

- Monitoring for infusion-related reactions will include allergic reactions, cardiac arrhythmia, GvHD and transmission of infectious diseases.
  As anaphylaxis cannot be precluded, CIK cell infusions must be administered where emergency resuscitative equipment and personnel trained in the management of anaphylaxis are immediately available to treat systemic reactions under the direct supervision of a physician. Anaphylaxis and cardiac arrhythmia both may occur while or shortly after infusion (for treatment

recommendations please see 3.2). Therefore, patients should be monitored overnight within the context of an inpatient hospital stay.
GvHD is expected to occur 2-4 weeks after infusion and may be treated by steroids or immunosuppressive medications.

- Before CIK cell infusion, one hour as well as one and two weeks after CIK cell infusion blood will be taken for assessment of blood counts, numbers of CD3-, CD4-, CD8-, CD56-, CD14- positive cells, cytokine profiles and soluble ligands of NKG2D receptor.

- Following CIK cell infusions, patients will be screened for GvHD according to Glucksberg Seattle Criteria (GSC, Appendix 1) every two weeks and will undergo bone marrow assessment every 3 months for efficacy analysis.

- CIK cell infusions will be given within a minimum of 4 and a maximum of 6 weeks between infusions provided that signs of acute GvHD (aGvHD) remained below grade II aGvHD. Second CIK cell dose will 5 x 10^6 $CD3^+CD56^-$ CIK cells/kg in pediatric and adult patients. Third CIK cell infusion and all following infusions will include 1 x 10^7 $CD3^+CD56^-$ CIK cells/kg in pediatric and adult patients. CIK cell infusions will be administered until the patient reaches molecular remission, provided that patients develop no more than grade I aGvHD.

  If the patient does not reach molecular remission, administration of CIK cell infusions will be stopped after the 8th CIK cell infusion, maximal duration of the study phase is one year.

  (For “Criteria for Discontinuation of the Treatment or Premature Withdrawal of the Patient” please also see chapter 8.1).

3.2 Patient Evaluation - Assessments

<u>Eligibility requirements for study:</u>

- Written informed consent of patient or legal representative
- Patient with acute leukemia or MDS after allogeneic stem cell transplantation and impending relapse (bone marrow or peripheral blood samples):
  - Detectable MRD
  - ≥ 10^{-4} BCR-ABL/ABL ratio
  - Mixed chimerism ≥ 1% autologous signals in two consecutive peripheral blood samples or one bone marrow sample (whole chimerism or chimerism subpopulations)

- Patients with acute leukemia or MDS with molecular relapse ≥ day 120 after allogeneic stem cell transplantation and less than 5% malignant cells in bone marrow analyses.
- Good clinical condition, Karnowsky or Lansky performance status > 50%
- No immunosuppression or steroids
- No chemo- or immune therapy, except patients with thyrosine-

kinase inhibitors (TKI) for BCR-ABL positive leukemias.

Observations during CIK cell infusion:

Following infusion of the product, the patient will be monitored for infusion related toxicity

Infusion related evaluations:

- Clinical examination: while cell infusion
- O^2 Saturation, Pulse, ECG: while and up to 60 minutes after CIK cell infusion
- Blood pressure: Every 10 minutes while and up to 60 minutes after CIK cell infusion

Acute Toxic effects

Infusional toxicity is defined as any of the following while and within 60 minutes after CIK cell infusion:
Allergic/hypersensitivity reactions,
Anaphylaxis,
Cardiovascular reactions (hypotension, hypertension),
Respiratory reactions (hypoxia, bronchospasm),
Neurological reactions (ataxia, encephalopathy and seizures),
Acute kidney and
Hepatic toxicities

Toxicity will be graded according to the NCI Common Terminology Criteria for Adverse Events version 4.0 for infusion related reactions, allergic/hypersensitivity reactions, anaphylaxis, cardiovascular (hypotension), respiratory (hypoxia, bronchospasm), neurological (ataxia, encephalopathy and seizures), acute kidney and hepatic toxicities. The criteria is available online at:
http://evs.nci.nih.gov/ftp1/CTCAE/CTCAE_4.03_2010-06
14_QuickReference_8.5x11.pdf

As acute toxic effects including anaphylaxis cannot be precluded, CIK cell infusions must be administered where emergency resuscitative equipment and personnel trained in the management of anaphylaxis, cardiovascular, respiratory and neurological reactions are immediately available to treat systemic reactions under the direct supervision of a physician. Therefore, patients should be monitored overnight within the context of an inpatient hospital stay.

Potential risks identified during previous administration of CIK cells – specified in the SmPC of the product – are:

- Infusion-related reactions
- Allergic reactions
- GvHD
- Cardiac arrhythmias
- Transmission of infectious diseases

Infusion-related reactions

In case of allergic reactions including anaphylaxis treatment of anaphylactic reaction is carried out according to ERC guidlines:

• Remove the trigger if possible
• Stop intravenous infusion of CIK cells.

• Do not delay definitive treatment if removing the trigger is not feasible.

Cardiorespiratory arrest following an anaphylactic reaction
Start cardiopulmonary resuscitation (CPR) immediately and follow current guidelines. Rescuers should ensure that help is on its way as early advanced life support (ALS) is essential. Use doses of adrenaline recommended in the ALS guidelines. The intramuscular route for adrenaline is not recommended after cardiac arrest has occurred.
Intramuscular (IM) Adrenaline
Monitor the patient as soon as possible (pulse, blood pressure, ECG, pulse oximetry). This will help monitor the response to adrenaline. The best site for IM injection is the anterolateral aspect of the middle third of the thigh. The needle used for injection needs to be sufficiently long to ensure that the adrenaline is injected into muscle.
Repeat the IM adrenaline dose if there is no improvement in the patient's condition. Further doses can be given at about 5-minute intervals according to the patient's response.
Intravenous (IV) adrenaline
There is a much greater risk of causing harmful side effects by inappropriate dosage or misdiagnosis of anaphylaxis when using IV adrenaline. In patients with a spontaneous circulation, intravenous adrenaline can cause life-threatening hypertension, tachycardia, arrhythmias, and myocardial ischaemia.
Oxygen
Initially, give the highest concentration of oxygen possible using a mask with an oxygen reservoir. Ensure high flow oxygen (usually greater than 10 litres min-1) to prevent collapse of the reservoir during inspiration. If the patient's trachea is intubated, ventilate the lungs with high concentration oxygen using a self-inflating bag.
Fluids
Large volumes of fluid may leak from the patient's circulation during an anaphylactic reaction. There will also be vasodilation, a low blood pressure and signs of shock. If there is intravenous access, infuse intravenous fluids immediately. Give a rapid IV fluid challenge (20 mL/kg in a child or 500-1000 mL in an adult) and monitor the response; give further doses as necessary. There is no evidence to support the use of colloids over crystalloids in this setting. Consider colloid infusion as a cause in a patient receiving a colloid at the time of onset of an anaphylactic reaction and stop the infusion. Hartmann's solution or 0.9% saline are suitable fluids for initial resuscitation. A large volume of fluid may be needed. If intravenous access is delayed or impossible, the intra-osseous route can be used for fluids or drugs when resuscitating children or adults, but only by healthcare
workers who are accustomed to do so. Do not delay the administration of IM adrenaline attempting intra-osseous access.
Antihistamines
Antihistamines are a second line treatment for an anaphylactic reaction. The evidence to support their use is weak, but there are logical reasons for them. Antihistamines (H1-antihistamine) may help counter histamine-mediated vasodilation and bronchoconstriction. They may not help in reactions depending in part on other mediators but they have the virtue of safety. Used alone, they are unlikely to be lifesaving in a true anaphylactic reaction. Inject chlorphenamine slowly intravenously or intramuscularly.
Bronchodilators

The presenting symptoms and signs of a severe anaphylactic reaction and lifethreatening asthma can be the same. If the patient has asthma-like features alone, follow the British Thoracic Society – SIGN asthma guidelines (www.brit-thoracic.org.uk). As well as the drugs listed above, consider further bronchodilator therapy with salbutamol (inhaled or IV), ipratropium (inhaled), aminophylline (IV) or magnesium (IV). Remember that intravenous magnesium is a vasodilator and can cause hot flushes and make hypotension worse.

Cardiac drugs

Adrenaline remains the first line vasopressor for the treatment of anaphylactic reactions. There are animal studies and case reports describing the use of other vasopressors and inotropes (noradrenaline, vasopressin, metaraminol and glucagon) when initial resuscitation with adrenaline and fluids has not been successful. Only use these drugs in specialist settings (e.g., intensive care units) where there is experience in their use. Glucagon can be useful to treat an anaphylactic reaction in a patient taking a beta-blocker. Some patients develop severe bradycardia after an anaphylactic reaction. Consider IV atropine to treat this.

GvHD

In case of GvHD treatment depends on the individual pre-treatment, the individual drug compatibility, the individual dynamic and the individual severety considering the usual step-by-step therapy (steroids - cyclosporin A/macrocyclic antibiotics with immunosuppressive activity – mycophenolate mofetil - mesenchymal stromal cells – extracorporal photopheresis) according to generally recommended guidelines.

In case of cardiac arrythmias treatment of arrythmia is carried out according to ESC guidlines:

The initial assessment and treatment of a patient with an arrhythmia should follow the ABCDE approach. Key elements in this process include assessing for adverse signs; administration of high flow oxygen; obtaining intravenous access, and establishing monitoring (ECG, blood pressure, SpO2). Whenever possible, record a 12-lead ECG; this will help determine the precise rhythm, either before treatment or retrospectively. Correct any electrolyte abnormalities (e.g., K+, Mg2+, Ca2+). Consider the cause and context of arrhythmias when planning treatment.

The assessment and treatment of all arrhythmias addresses two factors: the condition of the patient (stable versus unstable), and the nature of the arrhythmia. Anti-arrhythmic drugs are slower in onset and less reliable than electrical cardioversion in converting a tachycardia to sinus rhythm; thus, drugs tend to be reserved for stable patients without adverse signs, and electrical cardioversion is usually the preferred treatment for the unstable patient displaying adverse signs. More comprehensive information on the management of arrhythmias can be found at www.escardio.org .

Transmission of infectious diseases

Standard procedures to prevent infections, which can be transmitted by drugs derived from blood or plasma, include the selection of the donor, testing of the single donation and the plasma pool for specific infectious disease markers.

Nevertheless, when using drugs derived from human blood or plasma, infectious diseases transmitted by agents - even from unknown nature so far - cannot completely be ruled out. In case of an occurrence of an infectious disease this has to be treated according to the underlying diagnosis.

Follow up 1-2 weeks after CIK cell infusions

Before, one hour, one and two weeks after CIK cell infusion the patients will be monitored for immune responses. Therefore 7.5 – 10 mL of EDTA blood will be taken at above mentioned time points for assessment of blood counts, numbers of CD3-, CD4-, CD8-, CD56-, CD14-positive cells, cytokine release and tumor escape mechanisms. Blood samples will be gathered by stem cell laboratory:

University Hospital Frankfurt, Goethe University
Department for Children and Adolescents
Division for Stem Cell Transplantation and Immunology
Stem Cell Laboratory (House 32D)
Theodor-Stern-Kai 7, 60590 Frankfurt am Main, Germany
Phone: +49-(0)69-6301-4918, Fax: +49-(0)69-6301-83168

Follow up 2-4 weeks after CIK cell infusions (CIK cell infusions will be continued until patient reaches molecular remission, until a maximum of 8 infusions are reached, until the patient progresses or discontinuation criteria are met):
- History taking*
- Physical examination*
- Karnowsky/Lansky performance index*

- Body weight*
- Peripheral blood
    - Blood count*
    - Bilirubin*
    - Chimerism (monthly*)
- Bone marrow (every 3 months*)
    - Morphology or histology
    - Chimerism, including subpopulations if appropriate
    - MRD if appropriate
    - BCR-ABL/ABL ratio if appropriate

* Obligatory

Eligibility requirements for next CIK cell infusion (4-6 weeks after previous CIK cell infusion):
- No immunosuppression or steroids
- No chemo- or immune therapy, except patients with thyrosine-kinase inhibitors (TKI) for BCR-ABL positive leukemias.

Follow up after last CIK cell infusion:

Within one month and thereafter every three months:
- History taking*
- Physical examination*
- Karnowsky/Lansky performance index*
- Peripheral blood
    - Blood count*
    - Bilirubin*
    - Immune status (optional)
    - Chimerism*
- Bone marrow (optional)
    - Morphology or histology

Chimerism, including subpopulations if appropriate
MRD if appropriate
BCR-ABL/ABL ratio if appropriate

Once a year
- Ophthalmologist control
- Pulmonary function testing
* Obligatory

Other laboratory investigations will be performed as per routine clinical care after allogeneic SCT according to institutional guidelines.
Acute and chronic GvHD will be graded according to the Glucksberg scale (see Appendix 1)

3.3 Study Duration

The estimated duration of the recruitment period will be 3 years and the total duration of the study is expected to be at least 5 years.

The study duration per patient will include study phase during CIK cell infusions and observational phase after the last CIK cell infusion.
Study phase will last until the patient reaches molecular remission (= end of CIK cell infusions), progresses or until the patient has received a maximum of 8 CIK cell infusions (maximum duration of study phase 1 year).
Observational phase will last 1 year after the last CIK cell infusion.

Additionally, CIK cell infusions will be terminated if the patient meets discontinuation criteria (see chapter 8.1). “Off-protocol” patients will be followed up for another 28 days. Patients where CIK cell infusions are discontinued, but who stay in the protocol will be followed up for 1 year (see chapter 8.1).

3.4 Number of Patients

40 Patients will be included into the study. The aim is to include 20 patients in each group that is 20 children and 20 adults.

4 Study Population

Acute leukemia and MDS patients aged >0 and <80 years of both gender experiencing molecular relapse after allogeneic stem cell transplantation.

It is known that the amount of leukemia cells is very critical for every type of immunotherapy. As a consequence, chemotherapy is needed to reduce the percentage of leukemia blast cells below 5%. And for this, patients need to be “eligible for chemotherapy” in terms of their general medical condition. Antineoplastic chemotherapy however, is necessary to bring patients, with frank hematological relapse after allogeneic stem cell transplantation, in complete hematological

remission (<5% of leukemia blasts in the bone marrow) before immunotherapy may be started.

All chemotherapy given for remission induction is not part of the study.
Chemotherapy has to be stopped 3 days before CIK cell infusions could be given.

4.1 Inclusion Criteria

- Acute leukemia and MDS patients with detectable MRD, ≥ 10^{-4} of BCR-ABL/ABL ratio, or MC in PB or BM samples obtained during monitoring for relapse after allogeneic stem cell transplantation.
- Detectable MRD, or ≥ 10^{-4} of BCR-ABL/ABL ratio .
- Acute leukemia and MDS patients with MC ≥ 1% of autologous signals in PB samples confirmed by another PB or BM sample within one week. Patients with MC ≥ 1% of autologous signals in $CD33^+$ and/or $CD34^+$ subpopulations in PB samples confirmed by BM analyses within one week. Acute leukemia and MDS patients with MC ≥ 1% of autologous signals including signals in $CD33^+$ and/or $CD34^+$ subpopulations in BM samples.
- Patients without immunosuppressive agents and steroids.
- Patients without chemo- or immune therapy, except patients with thyrosine-kinase inhibitors (TKI) for treatment of BCR-ABL positive leukemias.
- Patients with < grade II GvHD.
- Patients with Karnowsky or Lansky performance status ≥ 50%.
- Patients and/or his/her legal representative having reviewed the patient information/informed consent form and have had their questions answered and have given written informed consent.

4.2 Exclusion Criteria

- Acute leukemia and MDS patients with hematologic relapse < day 120 after allogeneic stem cell transplantation.
- Acute leukemia and MDS patients with more than 5% malignant cells in bone marrow analyses.
- Patients with immunosuppressive agents or steroids.
- Patients with chemo- or immune therapy, except patients with thyrosine-kinase inhibitors (TKI) for BCR-ABL positive leukemias.
- Patients with ≥ grade II GvHD
- Patients with Karnowsky or Lansky performance status < 50%.
- Patients and/or his/her legal representative having reviewed the patient Information/informed consent form and have had their questions answered and have not given written informed consent.
- HIV-positive patients
- HBV/HCV patients
- Patients with prior solid organ transplantation
- Patients treated with any other investigational product within the last 28 days or five half-lives (whichever is longer).
- Hypersensitivity to any component of the study drug
- Female patients of child-bearing potential not agreeing to use a highly effective method of birth control resulting in a low failure rate (i.e. < 1%) when

used consistently and correctly.

- Male patients with female partners of childbearing potential not agreeing to use a highly effective method birth control resulting in a low failure rate (i.e. < 1%) when used consistently and correctly.
- Pregnancy/Breastfeeding
- Patients with severe infections or signs/symptoms of infection within 2 weeks prior to study start.

4.3 Allowed/prohibited concomitant therapies

Except for patients with BCR-ABL positive leukemia, no concomitant anti-neoplastic treatment is allowed. In the BCR-ABL positive leukemia patients tyrosine kinase inhibitors (TKI) are the standard treatment to control the disease. However, there is no cure possible with TKI as single treatment. As a consequence, these patients may be included into the study while receiving TKI during the CIK cell treatment.
In all other patients' concomitant antineoplastic therapy is not allowed. Antineoplastic chemotherapy however, is necessary to bring patients, with frank hematological relapse after allogeneic stem cell transplantation, in complete hematological remission (<5% of leukemia blasts in the bone marrow). Patients with molecular relapse who are still in hematological remission are eligible for this study.
All chemotherapy given for remission induction is not part of the study. Chemotherapy has to be stopped 3 days before CIK cell infusions could be given.

5 Investigational Medicinal Poduct - CIK cells

5.1 CIK Cell Donors

CIK cells are generated from PBMCs of original stem cell donors after written informed consent.

5.2 Donor Exclusion Criteria

- Positive for HIV, HBsAG, HBc, HCV, TPHA
- Contraindication for blood withdrawal
- Pregnancy or breast feeding
- Heart disease
- Myocardial infarction < 3 months
- Seizure disorder
- mental health problem
- Malignant diseases

5.3 CIK Cell Generation

CIK cells are expanded according to GMP-terms from PBMCs of original stem cell donors by addition of interferon IFNγ, activating antibody directed against CD3, IL-2 and IL-15.

5.4 CIK Cell Recipient

Patients with molecular relapse without aGvHD ≥ grade II can be included and will receive CIK cell infusions in increasing doses.

Starting dose of $1x10^6$ $CD3^+CD56^-$ CIK cells/kg recipient body weight will be increased in intervals of 4-6 weeks to $5x10^6$ $CD3^+CD56^-$, and $1x10^7$ $CD3^+CD56^-$ CIK cells/kg. Patients will be screened for relapse and signs of GvHD according to GSC. Relapsed patients will be censored. Patients who experience disease progression or < grade II aGvHD may receive modified CIK cell doses. No further CIK cell infusions will be administered in the presence of aGvHD > grade II.

5.5 Benefit-Risk-Assessment

Patients at risk of relapse after allogeneic stem cell transplantation will relapse in 100% without further treatment. In these cases the only chance of cure is a pre-emptive immunotherapy or a second allogeneic stem cell transplantation. The pre-emptive immunotherapy involves discontinuation of the immunosuppressive therapy and of donor lymphocyte infusions. The conventional donor lymphocytes doses are associated with a high risk of GvHD and therefore are limited in terms of the T cell dose. Thus the success of this treatment is less than 15%. Even the success of allogeneic stem cell transplantation is only at a maximum of 20%. Moreover, this approach is also associated with a high risk of morbidity and mortality.
The currently existing lack of sufficient data makes it difficult for us to identify all possible risks for the patients.
At present we estimate the GvHD risk of CIK cell infusion lower than the GvHD risk of DLIs. Due to the higher T-cell dose administered during CIK cell infusions, we expect a higher effectiveness than the above mentioned success rates of DLIs.

6 Safety Issues

Monitoring and documentation of adverse events and adverse reactions.

Monitoring and documentation of serious adverse events and serious adverse reactions.

6.1 Definitions

Adverse Event (AE)

An adverse event is any untoward medical occurrence in a patient or clinical trial subject administered a medicinal product and which does not necessarily have a causal relationship with this treatment. An adverse event can therefore be any unfavourable and unintended sign (including an abnormal laboratory finding, for example), symptom, or disease temporally associated with the use of a medicinal

product, whether or not considered related to the medicinal product. Pre-existing conditions, which worsen during a study, are to be reported as Adverse Events. They can become Serious Adverse Events if they fulfil one of the seriousness criteria described below.

All clinical adverse events (AEs) encountered during the clinical study will be reported on the AE page of the CRF.

Laboratory Test Abnormalities

Laboratory test value abnormalities as such should not be reported on the AE page of the CRF as adverse events unless in the investigator’s opinion this abnormality is considered to be clinically significant. All clinically significant laboratory test value abnormalities will be reported on the AE page of the CRF as adverse events.

Within the scope of this study, considering the underlying disease and the post-transplantat status, all laboratory test value abnormalities which regularly occur and which are expected will not be considered to be clinically relevant.

Adverse Reaction

Adverse reactions are all untoward and unintended responses to an investigational medicinal product related to any dose administered.

Serious Adverse Event (SAE) or Serious Adverse Reaction (SAR)

Any untoward medical occurrence or effect that at any dose- results in death
- is life-threatening
- requires hospitalisation or prolongation of existing hospitalisation
- results in persistent or significant disability or incapacity or
- is a congenital anomaly or birth defect

The definition and reporting requirements according to German Drug Law, GCP-V and ICH Guideline for Clinical Safety Data Management,

Definitions and Standards for Expedited Reporting, Topic E2 will be adhered to.

Suspected Unexpected Serious Adverse Reaction (SUSAR)

Any adverse reaction that is classified as serious and is suspected to be caused by the investigational medicinal product where the nature or severity of which is not consistent with the applicable product information (e.g. investigator's brochure for an unauthorised investigational product or summary of product characteristics for an authorised product).

To specify SUSARS within the scope of allogeneic stem cell transplantation:
Within the scope of allogeneic stem cell transplantation following cases of death have to be anticipated:
- Cases of death due to infections in the context of allogeneic stem cell transplantation
- Cases of death due to toxicity of the conditioning regime
- Cases of death as a result of recurrence of the underlying disease

6.2 Documentation and Reporting of serious adverse events

Serious adverse events must be documented on the paper SAE-form. Documentation of SAE must be as complete and detailed as possible.
Toxicities are to be graded and documented on the respective reporting sheets, according to the Common Terminology Criteria for Adverse Events v4.0 (CTCAE; http://ctep.cancer.gov). All serious adverse events (SAEs) which occur in the concerned trial are subject of reporting using the SAE-form. This is independent of the fact whether the investigator acknowledges any causal relation between the event and the investigational medicinal product (IMP) (=SAR, serious adverse reaction). This includes type of event, start of the event, duration, seriousness and cause (if evident). Any SAE must be reported within 24 hours to the Sponsor.

The investigator has to report all SAEs (see exceptions below) by faxing the SAE form immediately (within 24 hours) to the authorized representative of the sponsor:

Prof. Dr. med. Peter Bader
University Hospital Frankfurt, Goethe University
Department for Children and Adolescents
Division for Stem Cell Transplantation and Immunology
Theodor-Stern-Kai 7, 60590 Frankfurt am Main,
Study Office -FAX: +49 (69) 6301-85122

In case the required information is not available within the requested reporting window, follow up reports must be sent.

6.3 Documentation and reporting of suspected unexpected serious adverse reactions (SUSARS)

According to GCP-V it is the responsibility of the sponsor to report all SUSARs that occur in this trail to the principal ethics committee, the principal governmental authority, all governmental authorities concerned
with the clinical trial and all involved investigators. Local regulations may require additional reporting e.g. to local ethics committee. According to German regulations the sponsor needs to send this report as soon as possible, but at its latest:
1. Within 7 days in case of a life threatening event or a lethal event. Within the following 8 days all further relevant information needs to be made available.
2. For all other events within 15 days.

According to the national German GCP-V §13(4) any event that will alter the current risk/benefit evaluation must be reported within 15 days to the principal ethics committee and to the competent authority.

6.4 Annual safety reports

In addition to the expedited reporting, the sponsor or his authorized representative will submit, once a year throughout the clinical trial or on request a safety report to the competent authority and the Ethics Committee.

7 Ethics and General Study Administration

This study will be conducted in compliance with the AMG, the German GCP-V, ICH GCP guidelines and/or other applicable ethical and regulatory requirements.
Investigators must have sufficient time to conduct the clinical study in compliance with the study protocol. Furthermore, they have to accurately and completely enter study data in the CRF. Investigators are responsible for obtaining informed consent of the patients as well as for the preparation and maintenance of adequate case files in order to record observations and other data relevant for this clinical study. Besides, they have to file the study-related records in the investigator site file (ISF) and have to maintain its actuality. They will permit study-related monitoring visits, audits by the sponsor or its representatives as well as inspections by regulatory authorities. The investigator must provide direct access to the study site's facilities, to source documents, and to all other study documents.

Sponsor of the study is the Goethe-University Frankfurt, represented by the President.

7.1 Ethical Aspects and Independent Ethics Committee (IEC)

The Declaration of Helsinki (revision of 1996) is the accepted basis for clinical study ethics, and must be fully followed and respected by all engaged in research on human beings. Any exceptions must be justified and stated in the protocol.

The sponsor of the clinical trial has to submit all relevant study documents to the independent ethics committee (IEC) in charge, which for this study is the Ethics Committee of the Faculty of Medicine of the Goethe University Frankfurt *(central coordinator).* The study cannot be started unless a positive evaluation given by the ethics committee has been conveyed. Simultaneously to the central coordinator the local ethics committees (participating ethics committee) of the study centers will be informed about the planned study. The inclusion of new study centers has to be arranged by sending all relevant documents to the central coordinator and to the local ethics committee in charge at the same time. The study can be started in the new study center after the participating ethics committee in charge has validated the study center and the qualification of the investigator.

7.2 National Competent Authority (NCA)

According to §§ 40-42 of the German drug law (AMG) it is the responsibility of the sponsor to obtain and maintain independent approval from the federal regulatory authority (BfArM/PEI).

The national German competent authority (NCA) for this clinical trial is the Paul-Ehrlich-Institute (PEI). An approval by the NCA must be available prior to study start.

7.3 Notification of the Study

According to § 67 German drug law (AMG) the Sponsor is responsible to notify competent regional authority and the federal regulatory authority (BfArM/PEI) about the study and all principal investigators of the participating investigational sites. If no other agreements are made, the Sponsor will take over responsibility for investigator's obligation to report (§ 12 (3) GCP-V).

7.4 Insurance

All patients of the clinical trial are covered by an insurance at the HDI–Gerling Versicherung AG. The patient / patient’s parents will be explained the insurance coverage and conditions precedent and subsequent to the policy prior to the clinical trial. The patient/patient’s parents will receive a copy of the insurance policy.

7.5 Financing

Participating subjects of this study will not receive financial benefits.

7.6 Consent procedure

Patient Information and Informed Consent
It is the responsibility of the investigator to obtain written informed consent from each patient participating in this study, after adequate explanation of aim, importance, anticipated benefits, and potential hazards and consequences of the study according to § 40 Abs 2 and § 40 Abs. 2a AMG. Written informed consent must be obtained before any study specific procedures are performed. For patients not qualified or incapable of giving legal consent, written consent must be obtained from the respective legal representative/ his/her guardians. It must be also explained to the patients that they are completely free to refuse to enter the study or to withdraw from it at any time for any reason without incurring any penalty or withholding of treatment on the part of the investigator.

The patient’s guardians must give written consent prior to study participation. In case the patient is capable to understand her/his disease and the treatment concept of this study, she/he must be consented appropriately as an individual and give her/his consent separately.

By signing the consent form, the patient agrees with the "unwiderrufliche datenschutzrechtliche Einwilligung" according to § 40 Abs. 2a AMG. The patient also agrees to allow the monitor / auditor / health authorities to verify the collected patient data against the patient's original medical records for the purpose of source data verification.

The informed consent form personally signed and dated by the patient/ his/her guardians must be kept on file by the investigator(s), and documented in the case report form and the subject's medical records.

The investigator confirms obtaining the written informed consent to the sponsor.

7.7 Amendments

Each amendment of essential study documents has to be approved and generated by the sponsor and LKP. Favorable opinion of IEC and approval of the NCA is required for substantial amendments prior to implementation.

7.8 Data Protection

All study staff has to give due consideration to data protection and medical confidentiality. The collection, transfer, storage and analysis of personal study-related data are performed pseudonymized according to national regulations. The declaration of data protection is contained within the patient information/informed consent form.

8 Discontinuation or Early Termination of the Study

8.1 Criteria for Discontinuation of the Treatment or Premature Withdrawal of the Patient

Patients have the right to withdraw from the study at any time for any reason. The investigator also has the right to withdraw patients from the study in the event of intercurrent illness, adverse events, and treatment failure after a prescribed procedure, protocol violations, cure, administrative reasons or other reasons. Should a subject decide to withdraw, all efforts will be made to complete and report the observations as thoroughly as possible.

A complete final evaluation at the time of the patient’s withdrawal should be made with an explanation of why the subject is withdrawing from the study. If the reason for removal of a subject from the study is an adverse event or an abnormal laboratory test result, the principal specific event or test will be recorded on the CRF.

The following criteria lead to stop of additional CIK cell infusions; patients are remaining in the study:

- Development of GVHD > Grade II
- Response to CIK cell therapy: If mixed chimerism was the reason for study eligibility, than the achievement of complete donor chimerism leads to the stop of further CIK cell infusions. If the inclusion into the study was favored by minimal residual disease detection, the achievement of MRD negativity leads to a stop of additional CIK cell infusions.

- Severe infections

The following criteria lead to withdrawal from study; patients will be followed up for one month:

- Pregnancy of female patients
- Relapse of the underlying disease during CIK cell treatment requiring other treatment (e.g. systemic chemotherapy, second stem cell transplantation)

8.2 Criteria for Discontinuation of Termination of the Study

Both the sponsor and the investigator reserve the right to terminate the study at any time. Should this be necessary, both parties will arrange the procedures on an individual study basis after review and consultation. In terminating the study, the Sponsor and the investigator will assure that adequate consideration is given to the protection of the patient’s interests.

Criteria, which could lead to a discontinuation or termination of the study, are:
- Safety reason regarding patients' safety
- Negative benefit/risk assessment due to new information

In case of premature termination of the study all collected data have to be analyzed and a report has to be written. The sponsor has to inform the federal regulatory authority, the ethics committees and other authorities of member states of the European Union where the study is conducted within 15 days, giving detailed reason for the premature termination.

9 Publications Rules and Results

The sponsor is responsible for the timely reporting of study data – latest 2 years after last patient last visit. An integrated clinical study report (CSR) has to be completed one year after end of the study (whether completed or prematurely terminated).

The results of this study may be published or presented at scientific meetings as soon as after completion of the study. If this is foreseen, the investigator agrees to submit all manuscripts or abstracts to the sponsor prior to submission.

In a multicenter study, it must be ensured that the data from one center is not published before the publication of the whole study. The sponsor reserves the right to review the manuscript(s) before their submission for publication or presentation. This is not intended to restrict or hinder publication or presentation, but is to allow the sponsor to protect proprietary information and to provide comments based on information from other studies that may not yet be available to the investigator(s).

This study will be registered at http://www.clintrials.gov.

10 Monitoring

To ensure compliance with the protocol and with the ethical principles of Declaration of Helsinki and GCP, selected monitoring visits for source data verification by a professional representative of the sponsor will be scheduled to take place early in the study, during the study at predefined intervals and after the last subject has completed the study. In addition it
may be necessary for the sponsor or a Drug Regulatory Agency to conduct a site audit. This may occur at any time from start to after conclusion of the study.

All documents of this clinical trial (e.g. trial investigator file and documentary sheets) are to be kept in a secure place at the study center for at least 15 years after the final trial report has been published.

11 Statistics

Study Aims

This study is a prospective multisite, non-randomized open single-arm Phase I/II study in children and adults with leukemia and MDS with molecular relapse after allogeneic SCT.

The main purpose of this study is to assess the feasibility and the efficacy of CIK cells infusions to acquire essential information about the practicality of a posterior larger study. In this early stage about CIK cells infusions there are no sufficient information for an initial hypothesis therefore this study is explorative.

Exploratory Endpoints

Efficacy:

- Proportion of patients that achieve complete and partial response after 1 year of first CIK cells infusion.
- Progression-free survival and overall survival
- Response duration

Safety Endpoint:

- Proportion of patients who become aGvHD > grade II or extensive chronic GvHD.

CIK cells infusions:

- The dose-limiting toxicity based on grade III or IV acute GvHD

Sample Size

To estimate the efficacy of the CIK cells therapy in a single group, a sample in the range of 20-25 will probably be adequate due to the expected population effect sizes moderate.

Consequently, we choose a sample size equal to 20 children and 20 adults. We can analyze the efficacy considering the entire group as well as also adult and children separately. Furthermore, a sample size equal to 40 is large enough to provide useful information concerning assessment of feasibility.

Below we evaluate the adequacy of a sample size equal to 40 and 20. The margin of error is also called the confidence interval and is used to describe the degree of uncertainty we have in the sample estimate.

Confidence intervals for efficacy

To assess the efficacy of CIK cell treatment we consider the rate of patients who achieve a complete/partial molecular response following CIK cell infusion.

The two sided 95% confidence intervals for complete/partial molecular response rate from 5% - 50% for a sample size = 40 are

Rate	Lower Limit	Upper limit	CI Width
0.05	0.006	0.163	0.163
0.1	0.028	0.237	0.209
0.2	0.091	0.356	0.266
0.3	0.166	0.465	0.300
0.4	0.249	0.567	0.318
0.5	0.338	0.662	0.324

The two sided 95% confidence intervals for the rate (5% - 50%) for patients that achieved complete/partial molecular response for a sample size = 20 are

Rate	Lower Limit	Upper limit	CI Width
0.05	0.001	0.249	0.247
0.1	0.012	0.317	0.305
0.2	0.057	0.437	0.379
0.3	0.119	0.543	0.424
0.4	0.191	0.639	0.448
0.5	0.271	0.728	0.456

The following figure illustrates the CI width for different complete/partial molecular response rate for all patients (sample size = 40) and for each group (sample size = 20) with a two-sided confidence level equal to 95%.

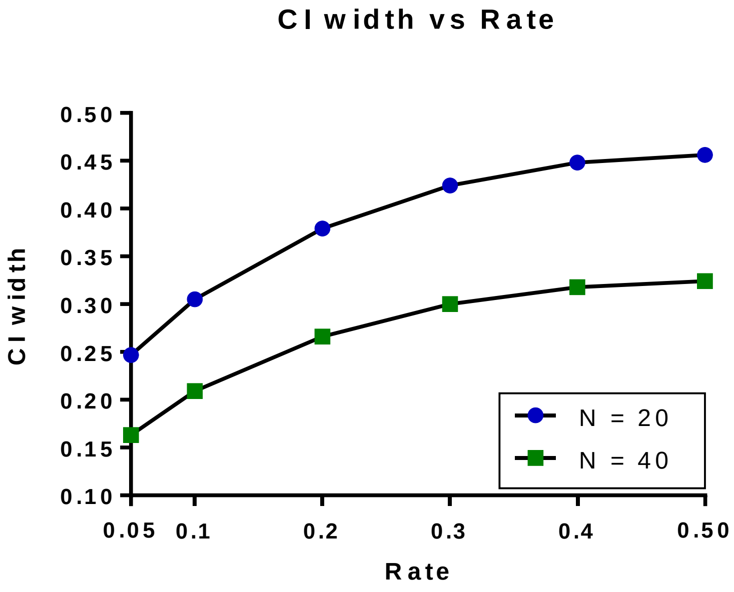

A sample size of 40 produces a two-sided 95% CI with a width equal to 0.318 when the complete/partial molecular response rate in the sample is 0.4. For the same response rate, a sample size of 20 patients produces a two-sided 95% confidence interval with a width equal to 0.448.

Confidence intervals for safety

The occurrence of grade III or IV acute GvHD and extensive chronic GvHD will be considered to evaluate the safety in this study. According to published results the aGVHD > Grad II incidence for a patient whit DLI treatment can be assumed 40%. We expected a lower incidence rate of aGVHD. We investigated given an incidence rate of having an aGVHD > Grad II equal to 5%, 10%, 20% and 30% then confidence interval width for different two-sided confidence levels in a sample size of 20 patients.

N	Rate	CI $width_{95}$	CI $width_{90}$	CI $width_{68}$
20	0.05	0.247	0.214	0.147
20	0.10	0.305	0.265	0.181
20	0.20	0.379	0.330	0.222
20	0.30	0.424	0.368	0.247
20	0.40	0.448	0.389	0.26
20	0.50	0.456	0.396	0.264

The next figure represents the CI width vs different aGVHD incidence rate for one group (sample size = 20) for different confidence levels.

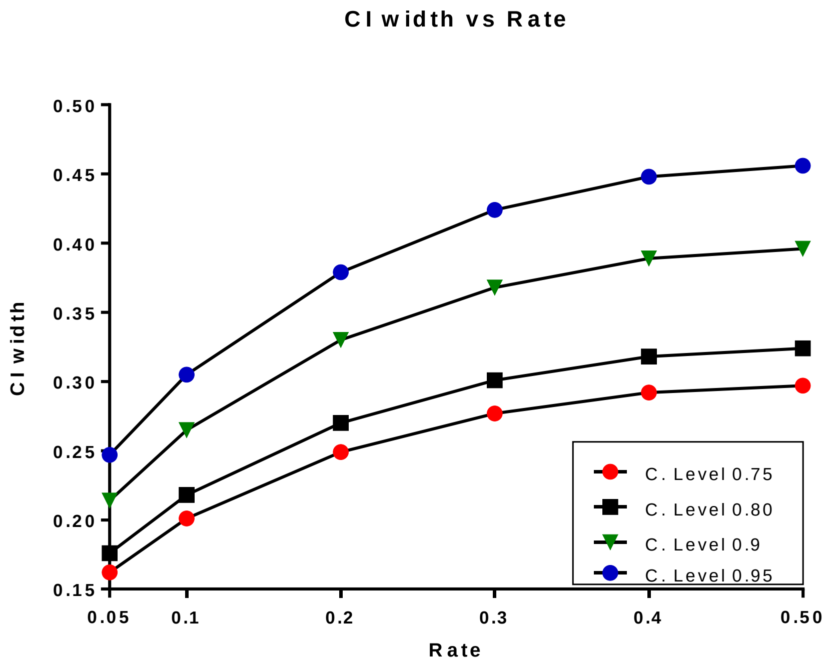

The estimated duration of the recruitment period is equal to 3 years according to our evaluated data in the last 10 years. Each center has treated ca. 6-7 patients per year who fulfilled the inclusion criteria defined in the protocol.

Analysis

A descriptive statistics will be performed at the end of the study. An intent-to-treat principle (ITT) or per-protocol analysis will be done.

To analyze the efficacy of CIK cell treatment following factors will be considered:

- Complete and partial molecular response
- rate and duration of complete/partial molecular response following CIK cell administration at the end of CIK cell infusions and after 1 year of first CIK cell infusion
- rate of and time to non response
- rate of and time to relapse with previous partial/complete response
- Progression-Free survival (PFS) and overall survival (OS)
- Cumulative incidences of: haematological relapse/progression, deaths without relapse/progression
- Cumulative incidence of aGVHD > grade II and extensive chronic GvHD
- Median and range for the number of CIK cell dose and for cumulative dose CIK cells/kg for all patients, children and adults

- Median and range for cumulative T cells/kg according donor type (MSD, MUD,MMFD) that patient requires in the study

Complete molecular response is defined as the molecular remission: the Minimal Residual Disease not detectable (MRD < 10-6) and Mixed Chimerism MC < 1%.
Partial response is defined as no molecular remission but not haematological relapse. Non response is defined as haematological relapse without previous or complete response.
The median of PFS and OS time will be estimated. Kaplan-Meier estimates will be used to estimate PFS probability and OS probability. Log-rank test will be used as appropriate. For PFS will be considered as events: disease progression or relapse, death without relapse/progression and secondary neoplasm. For OS death from any cause will be considered an event.
Gray-Model will be used for cumulative incidences. Patients lost to follow-up without event will be censored at the date of their last follow up evaluation.
PFS, OS and cumulative incidences will be estimated after 1 year initiation of CIK cell therapy and after 1 year until the last CIK cell infusion.

If the data meet the necessary requirements, longitudinal analysis will be conducted to explore the improvement of hematologic and immune reconstitution of leucocyte, CD3-, CD4- , CD8-, CD56-, and CD14-positive cells until the first year after begin of the CIK cells therapy.
The dynamic of cytokine profile during cell infusion and tumor escape mechanisms following CIK cell infusion will exhibit in curves over the time.

We will use a significance level of $\alpha = 5\%$.

12 Literature

Bader, P., et al. (1999). Prevention of relapse in pediatric patients with acute leukemias and MDS after allogeneic SCT by early immunotherapy initiated on the basis of increasing mixed chimerism: a single center experience of 12 children. **Leukemia** 13:2079-86.

Bader, P., et al. (2004). Increasing mixed chimerism is an important prognostic factor for unfavorable outcome in children with acute lymphoblastic leukemia after allogeneic stem-cell transplantation: possible role for pre-emptive immunotherapy? **J.Clin.Oncol.** 22:1696-1705.

Rettinger, E., et al. (2011). Preemptive immunotherapy in childhood acute myeloid leukemia for patients showing evidence of mixed chimerism after allogeneic stem cell transplantation. **Blood** 118:5681-5688**.**

Rössig, C., et al. (2011). T-Zell-Therapien bei Leukämie. **Monatsschrift Kinderheilkunde** 158:232-9.

Kuci, S., et al. (2010). Efficient lysis of rhabdomyosarcoma cells by cytokine-induced killer cells: implications for adoptive immunotherapy after allogeneic stem cell transplantation. **Haematologica** 95, 1579-86

Rettinger, E., et al. (2012). The cytotoxic potential of interleukin-15 stimulated cytokine-induced killer cells against leukemia cells. **Cytotherapy** 14:91-103**.**

Rettinger, E., et al. (2012). Cytotoxic capacity of IL-15-stimulated cytokine-induced killer cells against human acute myeloid leukemia and rhabdomyosarcoma in humanized preclinical mouse models. **Frontiers in Oncology** 32:1-10**.**

Schmidt-Wolf, I. G., et al. (1991). Use of a SCID mouse/human lymphoma model to evaluate cytokine-induced killer cells with potent antitumor cell activity. **J.Exp.Med.** 174:139-49.

Linn, P. H., et al. (1994). A noval population of expanded human CD3+CD56+ cells derived from T-cells with potent in-vivo antitumor-activity in mice with severe combined immunodeficiency. **J.Immunol.** 153:1687-1696

Linn, Y. C., et al. (2002). Generation of cytokine-induced killer cells from leukaemic samples with in vitro cytotoxicity against autologous and allogeneic leukaemic blasts. **Br.J.Haematol.** 116:78-86.

Sangiolo, D., et al. (2008). Alloreactivity and anti-tumor activity segregate within two distinct subsets of cytokine induced killer (CIK) cells: implications for their infusion across major HLA barriers. **Int.Immunol.** 20:841-8.

Sangiolo, D., et al. (2009). Cytokine induced killer cells as adoptive immunotherapy strategy to augment graft versus tumor after hematopoietic cell transplantation. **Expert.Opin.Biol.Ther.** 9:831-40.

Linn, Y. C., et al. (2009). Characterization of the recognition and functional heterogeneity exhibited by cytokine-nduced killer cell subsets against acute myeloid leukaemia target cell. **Immunology** 126:423-35.

Introna, M., et al. (2007). Repeated infusions of donor-derived cytokine-induced killer cells in patients relapsing after allogeneic stem cell transplantation: a phase I study. **Haematologica** 92:952-9.

Laport, G.G., et al. (2011). Adoptive immunotherapy with cytokine-induced killer cells for patients with relapsed hematologic malignancies after allogeneic hematopoietic cell transplantation. Adoptive immunotherapy with cytokine-induced killer cells for patients with relapsed hematologic malignancies after allogeneic hematopoietic cell transplantation. **Biol Blood Marrow Transplant.** 17:1679-87.

Rettinger, E., Bonig, H., Wehner, S., Lucchini, G., Willasch, A., Jarisch, A., ... & Bader, P. (2013). Feasibility of IL-15-activated cytokine-induced killer cell infusions after haploidentical stem cell transplantation. Bone marrow transplantation, 48(8), 1141-1143.

Kedmi, M., Resnick, I. B., Dray, L., Aker, M., Samuel, S., Gesundheit, B., ... & Shapira, M. Y. (2009). A retrospective review of the outcome after second or subsequent allogeneic transplantation. Biology of Blood and Marrow Transplantation, 15(4), 483-489.

Shaw, B. E., Mufti, G. J., Mackinnon, S., Cavenagh, J. D., Pearce, R. M., Towlson, K. E., ... & Russell, N. H. (2008). Outcome of second allogeneic transplants using reduced-intensity conditioning following relapse of haematological malignancy after an initial allogeneic transplant. Bone marrow transplantation, 42(12), 783-789.

Bosi, A., Laszlo, D., Labopin, M., Reffeirs, J., Michallet, M., Gluckman, E., ... & Frassoni, F. (2001). Second allogeneic bone marrow transplantation in acute leukemia: results of a survey by the European Cooperative Group for Blood and Marrow Transplantation. Journal of clinical oncology, 19(16), 3675-3684.

Hosing, C., Saliba, R. M., Shahjahan, M., Estey, E. H., Couriel, D., Giralt, S., ... & De Lima, M. (2005). Disease burden may identify patients more likely to benefit from second allogeneic hematopoietic stem cell transplantation to treat relapsed acute myelogenous leukemia. Bone marrow transplantation, 36(2), 157-162.

Bader, P., Kreyenberg, H., Henze, G. H., Eckert, C., Reising, M., Willasch, A., ... & von Stackelberg, A. (2009). Prognostic value of minimal residual disease quantification before allogeneic stem-cell transplantation in relapsed childhood acute lymphoblastic leukemia: the ALL-REZ BFM Study Group. Journal of Clinical Oncology, 27(3), 377-384.

Thiede, C., Bornhäuser, M., Oelschlägel, U., Brendel, C., Leo, R., Daxberger, H., ... & Ehninger, G. (2001). Sequential monitoring of chimerism and detection of minimal residual disease after allogeneic blood stem cell transplantation (BSCT) using multiplex PCR amplification of short tandem repeat-markers. Leukemia, 15(2), 293-302.

Bornhäuser, M., Oelschlaegel, U., Platzbecker, U., Bug, G., Lutterbeck, K., Kiehl, M. G., ... & Thiede, C. (2009). Monitoring of donor chimerism in sorted CD34+ peripheral blood cells allows the sensitive detection of imminent relapse after allogeneic stem cell transplantation. haematologica, 94(11), 1613-1617.

Linn, Y. C., Niam, M., Chu, S., Choong, A., Yong, H. X., Heng, K. K., ... & Koh, M. (2012). The anti-tumour activity of allogeneic cytokine-induced killer cells in patients who relapse after allogeneic transplant for haematological malignancies. Bone marrow transplantation, 47(7), 957-966.

Savani, B. N., Mielke, S., Reddy, N., Goodman, S., Jagasia, M., & Rezvani, K. (2009). Management of relapse after allo-SCT for AML and the role of second transplantation. Bone marrow transplantation, 44(12), 769-777.

Herzog M: Considerations in Determining Sample Size for Pilot Studies, Research in Nursing & Health, 2008; 31:180-191

13 Appendices

APPENDIX 1

Glucksberg acute GvHD scores

Skin

- Stage 0: No rash
- Stage 1: Maculopapular rash <25% of body surface area
- Stage 2: Maculopapular rash on 25-50% of body surface area
- Stage 3: Generalized erythroderma
- Stage 4: Generalized erythroderma with bullous formation and desquamation

Liver

- Stage 0: Bilirubin <2 mg/dL
- Stage 1: Bilirubin 2-3 mg/dL
- Stage 2: Bilirubin 3.01-6 mg/dL
- Stage 3: Bilirubin 6.01-15.0 mg/dL
- Stage 4: Bilirubin >15 mg/dL

GI

- Stage 0: No diarrhea, or diarrhea <500 mL/day
- Stage 1: Diarrhea 500-999 mL/day
- Stage 2: Diarrhea 1000-1499 mL/day
- Stage 3: Diarrhea >1500 mL/day
- Stage 4: Severe abdominal pain, with or without ileus

Glucksberg grade

**Overall grade**	**I**	**II**	**III**	**IV**
Skin	1-2	1-3	2-3	2-4
GI	0	1	2-3	2-4
Liver	0	1	2-4	2-4
Karnofsky/Lansky performance scale	90-100%	70-80%	50-60%	30-40%

IBMTR severity index

The severity is the highest level which the patient reaches based on separate skin, liver, and GI staging.

Overall grade	A	B	C	D
Skin	1	2	3	4
GI	0	1-2	3	4
Liver	0	1-2	3	4

APPENDIX 2

Karnofsky/Lansky performance status scale

Table 1. Karnofsky/Lansky Scale

Karnofsky Scale (recipient age ≥ 16 years)		Lansky Scale (recipient age <16 years)	
**Able to carry on normal activity; no special care is needed**		**Able to carry on normal activity; no special care is needed**	
**100**	Normal, no complaints, no evidence of disease	**100**	Fully active
**90**	Able to carry on normal activity	**90**	Minor restriction in physically strenuous play
**80**	Normal activity with effort	**80**	Restricted in strenuous play, tires more easily, otherwise active
**Unable to work, able to live at home cares for most personal needs, a varying amount of assistance is needed**		**Mild to moderate restriction**	
**70**	Cares for self, unable to carry on normal activity or to do active work	**70**	Both greater restrictions of, and less time spent in active play
**60**	Requires occasional assistance but is able to care for most needs	**60**	Ambulatory up to 50% of time, limited active play with assistance/supervision
**50**	Requires considerable assistance and frequent medical care	**50**	Considerable assistance required for any active play, fully able to engage in quiet play
**Unable to care for self, requires equivalent of institutional or hospital care, disease may be progressing rapidly**		**Moderate to severe restriction**	
**40**	Disabled, requires special care and assistance	**40**	Able to initiate quite activities
**30**	Severely disabled, hospitalization indicated, although death not imminent	**30**	Needs considerable assistance for quiet activity
**20**	Very sick, hospitalization necessary	**20**	Limited to very passive activity initiated by others (e.g., TV)
**10**	Moribund, fatal process progressing rapidly	**10**	Completely disabled, not even passive play

APPENDIX 3

CHRONIC GRAFT-VERSUS-HOST DISEASE (cGVHD)

Classification at the Time of Initial Presentation

The purpose of this classification is to identify patients with cGvHD who need long-term systemic immunosuppression according to clinical and laboratory findings and risk factors at the time of initial diagnosis. Long-term treatment with immunosuppression is indicated for patients with clinical extensive cGvHD and for patients with limited cGvHD and high-risk features (i.e., platelets count < 100,000 or ongoing steroids).

CLINICAL LIMITED cGVHD

1. Oral abnormalities consistent with cGvHD, a positive skin or lip biopsy, and no other manifestation of cGvHD.
2. Mild liver test abnormalities (alkaline phosphatase ≤2 x upper limit of normal, AST or ALT ≤3 x upper limit of normal and total bilirubin ≤1.6) with positive skin or lip biopsy, and no other manifestation of cGvHD
3. Less than 6 papulosquamous plaques or limited skin rash or dyspigmentation (<20% of the body surface), positive skin biopsy, and no other manifestation of cGvHD
4. Ocular sicca syndrome (Schirmer's test ≤5mm), positive skin or lip biopsy, and no other manifestation of cGvHD
5. Vaginal or vulvar abnormalities with positive biopsy, and no other manifestation of cGvHD

CLINICAL EXTENSIVE cGvHD

1. Involvement of two or more organs with symptoms or signs of cGvHD, with biopsy documentation of cGvHD in any organ >15% base line body weight loss not due to other causes, with biopsy documentation of cGvHD in any organ
2. Skin involvement more extensive than defined for clinical limited cGvHD, confirmed by biopsy
3. Scleroderma or morphea
4. Onycholysis or onychodystrophy thought to represent cGvHD, with documentation of cGvHD in any organ
5. Decreased range of motion in wrist or ankle extension due to fasciitis caused by cGvHD
6. Contractures thought to represent cGvHD
7. Bronchiolitis obliterans not due to other causes
8. Positive liver biopsy; abnormal liver function tests not due to other causes with alkaline phosphatase >2 x upper limit of normal, AST or ALT >3 x upper limit of normal, or total bilirubin >1.6, and documentation of cGvHD in any organ
9. Positive upper or lower GI biopsy

MANIFESTATIONS OF CHRONIC GvHD (DEFINITIONS OF SIGNS AND SYMPTOMS OF CHRONIC GvHD)

In all cases, concomitant processes (i.e., infections or drug reaction) must be ruled out. Karnofsky Clinical Performance score <60%, >15% weight loss, and recurrent infections are usually signs of clinical extensive cGvHD. Abnormalities that could indicate chronic GvHD are categorized by organ system as listed below.

Skin	Erythema, dryness, pruritus, pigmentary changes (i.e., hyperpigmentation, vitiligo), mottling, papulosquamous plaques, nodules, exfoliation, macular-papular or urticarial rash, scleroderma, morphea (one or several circumscribed, indurated and shiny lesions)
Nails	Ridging, onychodystrophy, onycholysis
Hair	Premature graying (scalp hair, eyelashes, eyebrows), thinning scalp hair, alopecia, decreased body hair
Mouth	Dryness, burning, gingivitis, mucositis, striae, atrophy, erythema, lichenoid changes, ulcers, labial atrophy or pigmentary changes, tooth decay, tightness around the mouth
Eyes	Dryness, burning, blurring, gritty eyes, photophobia, pain
Vagina/vulva	Dryness, dyspareunia, stricture or stenosis, erythema, atrophy or lichenoid changes not induced by ovarian failure
Liver	Elevated liver function tests not due to other causes (alkaline phosphatase ≥3 x upper limit, of normal, AST or ALT >4 x upper limit of normal or total serum bilirubin ≥2.5; in the absence of cGvHD involving other organs, liver biopsy is required to confirm the diagnosis)
Lung	Bronchiolitis obliterans (see diagnostic indicators), cough, wheezing, dyspnea on exertion, history of recurrent bronchitis or sinusitis
GI	Anorexia, nausea, vomiting, weight loss, diarrhea, dysphagia, odynophagia, malabsorption
Fasciitis	Stiffness and tightness with restriction of movement, occasionally with swelling, pain, cramping, erythema and induration, most commonly affecting the forearms, wrists and hands, ankles, legs and feet, inability to extend the wrists without flexing the fingers or the elbows, contractures
Muscle	Proximal muscle weakness, cramping
Skeletal	Arthralgia of large proximal girdle joints and sometimes smaller joints

LABORATORY TESTING AND DIAGNOSTIC INDICATORS OF CHRONIC GvHD

Eye	Schirmer's test with a mean value $\leq$ 5 mm at 5 minutes, or symptomatic with values of 6-10 mm or keratitis detected by slit lamp examination
Liver	Elevated liver function tests not due to other causes (see definition of clinical limited and clinical extensive cGvHD)
Lung	New obstructive lung defect defined as an FEV_1 <80% of predicted with either an FEF $_{25-75}$ <65% of predicted or RV >120% of predicted, or a decrease of FEV_1/FVC by >12% within a period of less than 1 year. A diagnosis of bronchiolitis obliterans requires negative microbiological tests from bronchoalveloar lavage and evidence of air trapping by high resolution end-expiratory and end-inspiratory CT scans of the chest. A thoracoscopic lung biopsy may be necessary in order to confirm the diagnosis of bronchiolitis obliterans in patients who have obstructive lung disease without air trapping when cGvHD involving other organs is absent.
Esophagus	Esophageal web formation, stricture or dysmotility demonstrated by barium swallow, endoscopy or manometry
Muscle	Elevated CPK or aldolase, EMG findings consistent with myositis
Blood	Thrombocytopenia (usually 20,000-100,000/μl), eosinophilia, hypogamma-globulinemia. Hyper-gammaglobulinemia and autoantibodies occur in some cases.
